# Spirit of the Foreign Periodicals

**Published:** 1832-07-01

**Authors:** 


### Spirit of the Foreign Periodicals, No. 1.

### VII.

### Secret Remedies.

It appears that in France, as in our own country, swarms of quacks and impostors have sprung up since the alarm of the cholera morbus first began, and have been puffing forth their infallible cures for this direful scourge. Our only motive for alluding to this subject at present is, to draw the attention of our readers to the much better legislation towards medical men that exists in the Continental governments than at home. There, a commission is appointed to examine into the merits of all nostrums, pretended specifics, secret cures, &c. and the Academy of Medicine, if not satisfied of their propriety, have full power and authority to interdict all such nuisances. If such privileges were enjoyed and exercised by the profession in this country, there would speedily be an end to all St. John Longs, Eadys, even when countenanced and supported by "regulars," and to those infamous disclosures which so frequently occur in this city.—*Journal Univers. et Heb.*

---

### VIII.

### CURIOUS FŒTAL MONSTROSITY.

THE abdominal muscles were entirely awanting, so that all the viscera were exposed and floating loose. They had been contained in a very delicate membrane, which was ruptured during parturition.

The ribs on the right side were almost of their normal development; those on the left were merely rudimentary.

The left upper extremity was very imperfect, presenting only a trace of the arm and of two fingers.—*Journal Univ. et Hebdom.*

---

### IX.

### GONORRHŒA IN FEMALES.

RICORD, surgeon of the Lock Hospital at Paris, has communicated an interesting memoir, wherein he proves that, in all contagious and obstinate vaginal discharges, small ulcers, in every respect like true chancres, are to be observed on the mucous surface of the vagina and of the os tincæ. The speculum uteri must be used to examine the parts. His treatment consists in introducing plugs of lint, wet with astringent and mercurial washes, and in exhibiting internal antisyphilitic medicines.

No such appearances are to be seen in leucorrhœa, and other innocuous discharges.—*Journal Univ. et Hebdom.*

---

### X.

### POISONING BY ARSENIC.

WE request the attention of our readers to the following case for several reasons; viz. the importance and difficulty of the subject—the errors and ignorance so frequently manifested by medical men on toxicological subjects, and the possibility of crimes of assassination being overlooked and mistaken, during the prevalence of any epidemic disease. Such is possible at the present time, as the following short statement will prove.

M. and Mme Caillette having eaten some *bouilli* and other meats at dinner, were seized, two hours after, with sickness and vomiting, which, however, by degrees ceased, and did not again return till next morning. Purging now supervened, and the stools were inodorous, and unhealthy, On the following day, the vomiting was attended with much anxiety and great prostration of strength, and a sensation of tightness at the throat.

The day after the above patients were seized, a domestic, who had also eat of the bouilli, became dangerously ill, with extreme exhaustion—feeble, whispering voice—pulse scarcely to be felt—involuntary twitchings of the muscles—vomiting and painful purging. She died 36 hours after seizure. Also a beggar, who had applied to the first patients for charity, had received some of the bouilli, which he voraciously devoured. Soon afterwards, violent vomiting and purging, extreme thirst, and universal tremors came on, and were succeeded by a state of coma. He, however, slowly recovered; but not so M. and Mme Caillette, who lingered, the former for 13 days, and the latter for 4 weeks. Before death, both suffered much, from a sense of burning in the throat, dysphagia, fever, aphthous ulcerations on the mouth and tongue, and a remarkable insensibility of the hands and feet; in short, the symptoms of chronic gastro-enteritis. Dissection revealed nearly the same appearances in all three.—Marks of vivid inflammation in the stomach and duodenum, and a morbid development of the glandulæ Peyeri and Brunneri in the ileum.

There was discrepancy of opinion among the two medical attendants as to the cause of the deaths—one suspecting that poison had been swallowed, the other referring the symptoms to a *choleroid* disease. The ignorance of chemical manipulation prevented the former from satisfying himself.

Some of the ejected matters, and also the stomachs of the deceased, were sent to Orfila for examination; and the presence of arsenic was speedily detected by him in the vomitings of the domestic, but not in those of the master and mistress. This is not surprising, if we consider the lapse of time between the seizure and death. It is to be remarked that a packet of arsenic was afterwards found in the house of Caillette, and it is supposed that it had been used for salting meat.—*Journal Univ. et Heb.*

---

### XI.

### Electricity and Cholera Morbus.

M. Converchel read a memoir to the Academy of Medicine, wherein he endeavours to prove, that electricity diffused through the atmosphere is the "source of life," and that, therefore, it must act a very important part in the production of all diseases, but especially of cholera; which he attributes to the deficiency or withdrawal of the electrical fluid from the organs of the body. He recommends warm bath, frictions, and galvanism.—*Archives Gener.*

When will some of our mercurial Continental brethren be satisfied with patient attention to facts, and a sober deduction of theory from these, and from these alone? If electricity be really, as M. Converchel alleges, the "fons et origo vitæ," every chemist, with his machine, might become a Frankenstein!—Ed.

---

### XII.

### Application of Auscultation to Midwifery.

In the majority of females, whose pregnancy is advanced beyond four months and a half, and in all during labour, under certain favourable circumstances, the pulsations of the heart of the fœtus may be distinctly made out, at least by those who are at all conversant with the use of the stethoscope. We must not be discouraged if, upon our first trial, we fail to recognize the sound of the fœtal heart, and the murmur of the uterine circulation, especially in the early months of gestation; we must repeat our experiments with assiduity and zeal, and our labours will surely be rewarded with results both interesting and important. We must expect to meet with difficulties of various kinds; we have already adverted to the faint obscurity of the sounds in early pregnancy; besides this, an inexperienced auscultator is apt to be deceived by the transmission of murmurs which are not seated in the uterus or its contents. The following is an interesting specimen.

A young woman, whose catamenia had ceased for five months, the abdomen enlarged, the cervix uteri soft, spongy, and flattened, and who stated that she had distinctly felt some motion, as if of the child, was admitted into the "Hospice de la Maternité." The stethoscope, applied to the lower and left side of the abdomen, transmitted a distinct noise of double pulsations, the number of which in the minute was 128. This is a slow pulse for a fœtus of the 6th month, and the physician, therefore, made a memorandum of the case, as rather singular:—Fortunately he examined the pulse of the young woman at this time, and found it to correspond in frequency with the pulsations heard. The stethoscope was re-applied to different parts of the abdomen, and the double beats were every where discovered, and becoming more and more distinct the nearer to the epigastric region that the instrument was put, proving that they arose from the accelerated action of the heart of the mother. On careful examination, no other sounds could be recognized, and the woman was declared not pregnant. The result shewed the accuracy of the diagnosis.

The above case shews that the sound of the mother's heart may occasionally be heard over the abdomen, and may easily lead the auscultator into error.

The usual number of the pulsations of the fœtal heart is from 140 to 150 in the minute; and this appears to be nearly the same at the different stages of pregnancy. Authors have disagreed as to the accuracy of the last conclusion; but we feel quite satisfied from the very numerous investigations which we have made, that it is perfectly correct; indeed we have repeatedly observed that the pulse was quicker at the full time than during the 6th and 7th months. It is very remarkable that when the pulsations of the fœtal heart are very distinct, we may frequently recognise a bellows-sound, very similar to what is heard in some diseases of the heart and large vessels—probably it is owing to the meeting of the two columns of blood in the aorta and pulmonary artery.

During a protracted labour the pulsations of the fœtal heart have been observed to become weak and to intermit, and these phenomena have been deemed by some as indicative of the suffering and danger of the child; but this conclusion is not very tenable, and it would be highly dangerous to assume it as a guide of our conduct; for often during gestation we find the pulsations to be strong and vigorous at one minute, and perhaps the next they are feeble and faint; and besides, we are well aware that the circulation of the child while still attached to and connected with the placenta may be active and undisturbed, and yet no sooner is the child born, but it dies, in consequence of the lungs refusing to act. Now we conceive that the most frequent cause of this *palsy* of the respiratory organs is an injury done to the brain, and thereby to the whole nervous system, during a tedious and severe labour, from the compression of the umbilical cord, and the consequent congestion of blood in the head. A case will best illustrate this view. A woman was delivered by the forceps after a severe labour; the stethoscope indicated the life of the child immediately before it was born, and when the cord was divided, two distinct jets of blood were thrown out; the heart was felt to beat for a few minutes, but no attempt at breathing was made, and the child therefore died.

Dissection showed the cerebral vessels enormously distended with blood, and the substance of the brain, lungs and liver to be much congested. In similar cases effusions have frequently been detected in the ventricles.—*Arch. Gener.*, *Janv.* 1832.

---

XIII.

UTILITY OF ACTUAL CAUTERY.

IN this country, we believe that the excellent effects of this remedy are overlooked; it is very much more alarming in anticipation, than in endurance:—some patients, Frenchmen we may guess, have even assured M. Jobert, that during the application of the hot iron they have experienced only a tickling and agreeable sensation!! [We recommend the Harley-street quack to visit Paris immediately, in search of such a delectable discovery.]

M. Jobert has employed the actual cautery, and also moxas in numerous cases of scrofulous swellings of the knee and other joints, with much, and very decided success. Many limbs condemned to amputation have been thus saved; and when the bones had not become carious, the motion of the articulation has been restored. Local palsies, and neuralgias, have been speedily cured; but our chief object in this notice is to direct the attention of our readers to the employment of the cautery in the treatment of that very intractable disease of the eye-lids, trichiasis. A man who had suffered from this complaint for ten years was speedily cured by one application of the cautery. The thin edge of a red-hot spatula was drawn along the eye-lid from one commissure to the other; the eschar formed was about 1½ lines in breadth; and was sufficiently deep to destroy the skin, cellular tissue, and palpebral aponeurosis, without affecting the tarsus and ciliary bulbs. Delpech, who first proposed the cautery in trichiasis, has treated many cases successfully by its use.

M. Jobert narrates a case of ulcer of the cervix uteri which he speedily cured by one application of the actual cautery.

It is proper to mention that our author distinguishes three different methods of employing the heated iron, either by applying it to the part with such a pressure as to destroy the texture deeply; or by drawing rapidly and very lightly the edge of the instrument over the part; and thus not disorganising all the thickness of the skin; or lastly by applying the point of a conical cautery, so as to make several small eschars. The pain felt after the application is best assuaged by cold water.—*Journ. Univ. et Hebdom.*

---

### XIV.

### Fracture of the Neck of the Femur.

[Extracts from Dupuytren's Clinical Lecture.]

The head of the femur has in some rare cases been driven through the bottom of the cotyloid cavity and fairly lodged within the pelvis;—and so firmly has it been impacted in this situation that even after death, it required much force to displace it. Falls upon the trochanter, and upon the soles of the feet have not unfrequently caused the head of the femur to be splintered, while the cervix was unhurt: but this accident is generally the result of contusions from shot; upwards of a dozen of such cases were seen by the Baron in July, 1830.

The most common situation of the fracture of the cervix is not at its middle, where it is weakest and most slender; but at its base, where it is united with the trochanter, which is not unfrequently broken at the same time, and is sometimes completely detached from the body of the bone. Dupuytren advocates the opinion that bony union may and does take place in intra-capsular fractures. Many specimens preserved in the museums of the Faculty of Medicine, and of the Hôpital de la Pitie, he assures us, distinctly prove this. The fracture is sometimes not complete, or the rough projections of the broken surfaces fit so closely to each other, like mortised work, that no displacement takes place for several days or even weeks. Sabatier reports a case, where twenty-two days elapsed, before the symptoms of fracture were apparent. The length of time necessary to the solid knitting of fractured cervix femoris is from 100 to 160 days, during the whole of which period, the extending apparatus must be constantly applied. Dupuytren has more than once seen cases where the provisional callus has given way when the patients were permitted to rise after two or three months; and thus shortening and deformity of the limb were occasioned. We must not always expect to find the foot turned outwards in this accident; the very reverse has frequently been noticed, and Bichat goes so far as to state that it occurs in the proportion of two cases in 10; a proportion however considerably greater than is warranted by Dupuytren's practice. The eversion of the foot is, in the opinion of the Baron, referrible to the action of the adductor muscles.

In the treatment of this accident, the patient ought to be laid on his back, the thigh bent on the pelvis, and the leg on the thigh; moderate extension may now be used with much advantage, as the muscles, which chiefly resist the adjustment of the two pieces are relaxed by the semiflexed position of the limb; the upper, or outer, or displaced portion is thus easily reduced, and brought in contact with the lower, or inner fragment; in which situation the object of the surgeon is to retain it. All apparatuses of continued and forcible extension are bad, and the following very simple contrivance, on the principle of permanent relaxation, is recommended as more simple and efficacious:—we must form a double-inclined plane with several pillars rolled firmly upon themselves into the form of bolsters, placed one above the other, and secured together by stitching; and along this double plane, we are to place another pillow lengthwise, extending from the hip to the heel—the leg and thigh are retained in their places by sheets folded round the limb after the manner of a cravat, and secured by their ends to the posts of the bed. During the first month, the surgeon ought to raise the thigh a little almost every day, and gently to draw the limb downwards. When he has reason to believe that the consolidation is effected, the double plane should be gradually lowered, by removing the pillows, one by one.

By this simple contrivance Dupuytren has succeeded in obtaining numerous cures with little or no shortening of the limb.—*Journ. Univ. et Hebdom.*

---

### XV.

### OBSTINATE CHRONIC OPHTHALMY WITH ULCERATION AND OPACITY OF THE CORNEA.

M. LE PELLETIER strongly recommends the use of the argenti nitras as a caustic, and calomel to be blown upon the eye every second or third day. One example will suffice to shew his practice.

A man aged 30 years had for many years suffered from repeated attacks of chronic ophthalmy, which had left puffy swellings of the eyelids, opacities of the cornea. The vision of the right eye was lost; and that of the left one nearly so, as the inferior half of the cornea was quite opaque, and the upper half nebulous and dim; there were also two large ulcers on it. Constant pain, weeping, and intolerance of light distressed the patient.

Calomel was ordered to be blown into the eye every second day, without any previous treatment; a calomel collyrium was also ordered. In five days the extreme sensibility had ceased entirely; the eyes could bear the light; the redness and swelling of the conjunctivæ were gone; and the opacities of the cornea considerably diminished. In a fortnight more he was so much improved, as to be able to leave the hospital.—*Ibid.*

---

### XVI.

### A SAGACIOUS AND IMPORTANT REFLECTION.

WHEN we assemble a number of facts and endeavour to draw legitimate inferences from them, we must *weigh*, not *count* them. "Non numerandæ solum, sed etiam perpendendæ sunt observationes."

---

### XVII.

### LETTER ON CHOLERA. By BARON DUPUYTREN.

*February*, 1832.

THE history of this fatal malady is deficient chiefly in very exact information of the true seat of the evil, and of the nature of the organic lesions produced in the intimate texture of our bodies. Some authors have supposed that the brain is the original seat of the disease; others have referred it to the spinal marrow; or to the semilunar ganglia and sola plexus; or to the heart. It appears to me that these opinions are little probable; but it would be well to verify them by very careful dissection, and by a minute examination of any changes found in these viscera.

The seat and character of the pains, and the nature and profusion of the discharges, lead the mind to suppose that the stomach and bowels must be the organs which primarily and principally suffer. Hitherto, however, it must be confessed, no very constant, or uniform morbid appearances have been detected: but to account for this, it is well to keep in mind that most of the dissections have been only of such patients as have very rapidly been carried off: to obtain satisfactory information, we must direct our examinations especially to the bodies of those who have resisted the disease for some time, and in which, therefore, it is reasonable to suppose that more distinct and obvious effects have been produced. Moreover it appears to me that the dissections have seldom been performed with a sufficiently inquisitive minuteness and accuracy.

As one of the most striking symptoms of cholera, is the immense quantity of a thin, watery, and almost insipid fluid discharged by vomiting and purging, is it not reasonable to direct our researches in an especial manner to those viscera which furnish this fluid? Now these viscera must be either the pancreas, the liver, or the alimentary canal; and if this last, then it must be that part of the tissue which supplies its peculiar secretions, viz. the small follicles (or glands of Peyer and Brunner) which are situated in the substance of the mucous membrane, and which are found clustered in certain parts of the bowels, known to all anatomists. The quality of the evacuations assures us that it cannot be the liver, and we are therefore led to believe that the offending organ must be either the pancreas or the secretory apparatus of the intestines. I am persuaded that a very attentive inspection of these follicles by the microscope will reveal in their cavity, walls, or surrounding substance, in their development, in the morbid changes of their texture and of their secretions, the seat and perhaps even the nature of cholera.

This is not mere conjecture: I have frequently examined the bodies of persons who have died of sporadic cholera, and I have uniformly found the glands of Peyer and Brunner exceedingly enlarged, and yet without any very distinct traces of inflammation. I do not believe that the epidemic differs so materially from the occasional cholera as to be attended with any marked difference in the seat and nature of the disease.

I would say therefore that the cholera has its seat in the alimentary canal in general; and more particularly in the follicles of the stomach and small intestines; that it essentially consists in a "*secretory irritation*" of these organs; that this gives rise to the dreadful pains of the bowels, and to the profuse watery evacuations; that the symptoms of disturbance in the functions of the brain, spinal marrow, particular nerves and the muscles to which they are distributed; of the heart and lungs, &c. are to be considered only as the sympathetic effects of the original disease; effects quite analogous to what we witness in malignant dysenteries which are attended with severe pains and excessive purging.

If these ideas be confirmed by dissection, it would follow, that the preventive treatment of cholera should consist, in guarding the surface of the body most strictly from the impression of cold and of moisture, by the use of flannel worn next to the skin; and in avoiding whatever tends to excite, or irritate the stomach and bowels, such as acrid, heating food and drink. The curative measures, when the disease once manifests itself, which are likely to be most useful, are leeches applied to the pained parts of the belly; composing drinks, (such as a decoction of poppy heads sweetened with syrup of gum, and administered frequently) and the exhibition of plumbi acetas, either in the form of pills, or dissolved in the decoction. I may remark that the preparations of opium are not so efficacious as the decoction of the poppy heads. The former have, in my practice, repeatedly failed, where the latter succeeded to my wish; and the acetate of lead, a sedative "par excellence" in all cases of inflammation attended with copious discharges, has been productive of better effects in sporadic cholera, when exhibited in a state of solution, than formed into pills.

Besides these means, the patient ought to be enveloped in warm blankets; frictions most perseveringly employed, and every now and then the steam of water introduced under the bed-clothes, so as to form a hot atmosphere round the body. We must carefully avoid, at least in the commencement, all purgatives, emetics, and irritants, which have been so freely and perniciously ordered by many, for they cannot fail to aggravate the irritation which is the essential character of the malady, and thus to hasten its fatal termination.

P. S. The dose of the acetate of lead may vary from 10 to 25 grains in the course of a day; it will be best given by ordering 3, 4, or 5 grains in a cup of the decoction, every hour or so, until the patient experiences some relief of suffering, and the evacuations are diminished. This mode of treatment has succeeded admirably in several cases of most severe sporadic cholera, which have occurred in my practice.

Appended to the preceding letter, are some critical remarks by the Editor of the Revue Medicale. He finds much fault with the Baron for presumiug to say that the dissections have not hitherto been conducted with sufficient minuteness and skill, and reminds him of the swarm of young medical men who have left the anatomical schools of France to visit those countries in which the epidemic was raging.

He tells him that he is mistaken in asserting that profuse evacuations are an essential symptom of the cholera; death may take place, even where there has been trifling vomiting and purging. Again, his opinion as to the identity of the nature and seat of ordinary and of epidemic cholera, cannot be strictly correct, even upon his own premises; for surely the Professor will not deny that the liver, which may be put "hors de question" in the consideration of the latter, is intimately and immediately involved in the former, as is proved by the bilious dejection?

It appears very strange that the Professor has omitted any allusion to the exhalant vessels of the intestines, and directed his suspicions solely to the mucous follicles on their surface. Are not they much more capable of producing the liquid stools than the small glands, which are irregularly scattered, and in some places appear almost altogether deficient.

The circumstance of M. Dupuytren having found the glandulæ Peyeri et Brunneri enlarged in several cases of sporadic cholera, cannot be considered as of much avail; for so common is this morbid appearance, in some continued fevers, that a distinguished physician has proposed to designate these by the name of "dothinenterites," from the Greek words "δοθιην," furunculus, and "εντερα," intestina.

For these reasons, we think that the distinguished author has been led into error, by assuming the *effects* of the disease for the *causes*; as well might we say that the enlargement of the spleen was the cause of intermittent fevers, because such a morbid state is almost invariably found in patients who *have died of these.—Revue Médicale.*

---

### XVIII.

### DISEASE OF PYLORUS, CURED BY NARCOTICS.

A MAN, ætatis 57, addicted to excesses in wine and women, had been reduced from a state of comfort to much bodily and mental distress. His earliest symptom was the feeling of a dull oppression in the region of the cardia; to this succeeded a pungent pain, always aggravated by wine or other stimulants. Dyspepsia, pyrosis, and increased flow of a sharp acid saliva, which set his teeth on edge, reduced his strength and flesh amazingly, and so imperfect was the digestion of his food, that it was generally passed in part unchanged; the abdomen was swollen, hard, and elastic, and the bowels were obstinately constipated. He had been in this condition for a year and a half, when he consulted M. Virey in March, 1831:—his appearance then was deplorable—his sufferings, especially at night, very severe, for no sooner did he lie down in bed, than his food was immediately vomited, accompanied with sour belchings, hiccough, and great anxiety; his stomach now could not digest the lightest farinaceous food. Various physicians had been consulted, and various modes of treatment tried, but all to no avail; leeches, blisters, tonics, stomachics, had only aggravated his distress. Every thing indicated the approach, or the actual presence, of organic disease of the pylorus, and the suspicion was strengthened by the lancinating pains felt when the ensiform cartilage was pressed. M. Virey had a most unfavourable opinion of the case.

*Treatment.* Warm clothing, with an occasional warm bath to soften the skin, which was harsh and dry; his diet to consist of milk, rice with sugar, white, gelatinous meats, and the lightest kinds of fish without any spices. No food to be taken in the evening, in order that the stomach might not be loaded during sleep. By attention to these rules he improved a little; but still, every now and then, his sufferings returned in all their intensity, from the slightest mental or physical cause. To lull this excessive susceptibility, resort was had to the syrupus papaveris, in doses at first of ʒij. and gradually increased to ℥j. and with much success, for the sickness, belchings, and vomiting ceased, and calm sleep was procured; the medicine, however, lost its efficacy in a few weeks. Opium, in the form of pills, was administered, and a Burgundy-pitch plaster, sprinkled with crude opium, applied to the epigastrium. The patient was better for the next two months; a diarrhœa supervened, which, on the whole, appeared to relieve the symptoms, as the digestion was less painful and imperfect during its continuance. By a steady perseverance in the mild and regulated diet, and in the use of the opium, a complete cure was effected in nine months, and he was restored to strength, cheerfulness, and plumpness. It was uniformly observed, that all tonics and stimulants on the one hand, and, on the other, whatever was enfeebling, invariably aggravated the symptoms and threatened a relapse. —*Revue Medicale.*

---

### XIX.

### Treatment of Scrofula by Iodine.

M. Baudelocque, physician "de l'Hôpital des Enfans," communicates an interesting memoir on the results of his practice in strumous disease. Sixty-seven girls, from the age of four to fifteen years, were treated by iodine in its different preparations. In all, the disease had existed for a considerable time, varying from nine years to ten months. Fifteen of these children were completely cured; fourteen so much improved, as to indicate a speedy cure; thirteen experienced less benefit than the preceding, but still sufficient to warrant hopes, though distant, of their recovery; five exhibited little, and twenty no amendment at all. All these patients with scarcely an exception, were much worse when they commenced the use of the iodine, than at the period of their admission into the hospital. Far from having derived any advantage from their residence there for several months, or even years, their condition was manifestly less favorable. This remark is well worthy our study, as it disproves the assertion of those medical men, who state that scrofula has a definite duration in the system, and that, after a certain lapse of time, it is easily cured, whatever remedies are employed. M. Baudelocque is satisfied, that the length of time during which the disease has existed in any case, had no influence in assisting or in quickening the cure of the numerous patients under his care.

Of the 67 cases, 17 had enlargement of the glands of the neck, jaw, and armpits; they took iodine inwardly, and employed likewise ioduretted baths, and friction with ointments of the potassæ hydriodas, plumbi ioduretum, et hydrargyri proto-ioduretum.

The mode in which the discussion of these enlargements is effected, makes us well acquainted with the nature of their formation; they consist of an aggregation of numerous small tumours, united together by cellular texture, which, in course of time, becomes firm, and increased in extent. In proportion as this cellular substance is dispersed, and returns to its normal state, the small glands, which are less quickly absorbed, become moveable and disjoined, and these, again, are resolved into several still smaller, until they quite disappear. To prevent the deformity of the scars so frequently left after a scrofulous abscess, the loose skin must be destroyed, either by the knife or by caustic, during the cicatrization of the sore, and a new healthy skin will be formed, so as to cause little disfigurement.—*Revue Médicale.*

---

### XX.

### NATURAL HISTORY OF MAN.

M. GEOFFROY SAINT HILAIRE concludes an interesting memoir by stating that females are generally much shorter than the males, in those races of mankind whose stature is great; the difference being much less among people of low stature;—that the races which are most remarkable for tallness, for the most part inhabit the southern hemisphere, while the most diminutive are found in the northern one;—that among the very tall races some live in South America, others in the South Pacific Archipelago, between the 10th and 50th degree of South latitude;—that some very diminutive races live in the southern hemisphere; and that some tall races are found in the northern hemisphere ; and if we compare the geographical position of these various divisions of mankind, we arrive at the curious result, that the diminutive races are almost always found near the very tall, and the very tall races near to those of low stature; that the variation in the size of different races may be explained in part by the influence of climate, diet, and mode of life—that it is extremely probable that the stature of the human race has not sensibly diminished, since the earliest age of history, or even from long anterior to that epoch.—*Revue Médicale.*

### XXI.

### ABSCESS OF THE HEART.

THE patient, a young soldier, died on the 55th day of confluent small-pox. A month before his death, an unhealthy abscess appeared on his left elbow; the fore-arm and hand of the same side were much swelled, and were cold to the touch; he lay on his right side, almost motionless from extreme weakness, moaning, but he did not complain of any local pain. Dissection shewed a heart somewhat larger than usual; the left side alone was hypertrophied. At the base of the left ventricle, posterior to the mitral valve, and imbedded in its fleshy substance, was found an abscess of the size of a hazel-nut, containing a white, homogeneous and fluid matter—it did not communicate either outwardly or inwardly, and was completely enclosed in a cyst.—*Revue Médicale.*

### XXII.

### MEDICAL JURISPRUDENCE. — ON THE DECOMPOSITION OF THE ANIMAL STRUCTURE.

M. OLIVIER and M. Denis were desired to examine the body of a female, who had been interred three months before, as suspicions had been excited that she had been murdered by her husband, and had died from the effects of blows upon her head, and fracture of the skull. The husband alleged that he had obtained the authority for interment from the physician appointed to ascertain the cause of his wife's decease; but it appeared that the physician had never examined the corpse, but had trusted to the husband's declaration that she had died from a rupture of a blood-vessel. The deceased was 55 years of age, and had been affected with hemiplegia for nine years. The body was buried the day after her death, in a dry chalky soil.

When exhumed, it did not present the appearance of much decay; the external surface, and the pectoral and abdominal viscera were little altered; but on examining the head, the right hemisphere was found softer and more decomposed than the left one, the anterior lobe was converted into a fatty matter, semi-consistent, and quite similar to what covered all the surface of the brain and spinal marrow, in the place of the pia mater, which had altogether disappeared. A similar greasy substance was discovered in both pleuræ, posterior to the lungs, which were quite shrivelled and dry. M. M. Olivier and Denis were led to the conclusion, that adipocire was formed at those parts where blood, or a bloody fluid had been deposited, and that therefore this female had died of a cerebral hæmorrhage. Such was their opinion, founded partly on the greater softness of the right hemisphere, and partly on the fatty degeneration of the anterior lobe, which indicated that it had been the seat of the hæmorrhage; else how was the fatty deposit not found in other portions of the cerebral substance? The occurrence of a similar matter in the two pleuræ is easily accounted for on the same view of the subject; for we know that in rapidly fatal apoplexies, there is generally an excessive congestion of blood in one or in both lungs; and it is but probable to suppose that after death there may be a gradual sudation of the same into the cavities of the pleuræ.

The husband was acquitted on the strength of the medical evidence.—*Revue Médicale.*

---

XXIII.

LETTER ON CHOLERA, BY DR. OCHEL OF PETERSBURG.

BY far the greater number of medical men in St. Petersburg are of opinion that this epidemic is *not contagious*, but that it is propagated by aerial miasmata and exhalations from the earth. The sudden appearance of the pestilence, and the rapidity with which it diffuses itself whenever it appears, strongly corroborate this idea. Moreover, many who escape the disease in its violent and concentrated form, suffer from *choleric* or *choleroid* attack, which are to be viewed only as diminutives of the great original; thereby distinctly proving that its severity and fatality are in proportion as the patients are predisposed to be affected by the influence of the atmosphere.

Dr. Ochel contends that the proximate cause of the disease is a paralysis of the organ of circulation. His remarks apply chiefly to the third stage, or what has been called the "stadium reactionis," which varies exceedingly in its character in different individuals. In many it commenced with delirium, which was speedily followed by coma, and numerous patients died in this state; in others there were neither delirium, nor coma, but inflammatory attacks of different viscera, sometimes of the liver, and not unfrequently of the parotid gland; in a third class he observed, fevers of various types, gastric, bilious, inflammatory, typhoid, or even intermittent. The treatment which he found by far the most effectual, consisted in giving repeated doses of common sea-salt in tepid water, till it produced bilious vomitings and stools. Out of a great number of cases, he selected 15 of the most aggravated; in each of these he gave the solution of salt, and in every case bile was copiously evacuated in less than an hour: 13 were saved and two died. When the bile passed downwards, either at the time, or on the morrow, he observed that the patients were generally cured in three or four days; when it did not, they were subject to relapse of particular symptoms, as watery vomitings, cold extremities, &c. &c.; but these threatenings vanished as soon as the bile was evacuated by a few spoonsful of tincture of rhubarb. Some of the medical men tried emetic doses of ipecacuan and tartrate of antimony, and found that nearly the same success was obtained, provided a sufficient quantity of bile was evacuated. Even the lower orders in Russia, who refused to apply for medical assistance, resorted to large quantities of oil and tepid milk to excite vomiting, and frequently with good effects.

From these facts, Dr. Ochel infers that the correct mode of treating cholera is to evacuate the bile as quickly as possible; and that whatever tends to check the vomiting and purging, before this effect is produced, is decidedly pernicious; for though death may be thus prevented at the onset of the disease, the foundation is almost always laid for the secondary disease, or "stadium reactionis," which never takes place when the bile has been freely discharged in the first stage. Dissection of rapidly fatal cases adds confirmation to this view of the subject; the gall-bladder is found distended with bile, and the ducts closely contracted. We must carefully distinguish the morbid appearances in such as have died in the early stage, from those which we observe after the secondary disease, under any of its different modifications, has commenced.

Many of Dr. Ochel's colleagues observed that the best preservative against an attack of the epidemic was a powerful emetic. As the disease began to subside in Russia it was noticed that numerous cases of bilious vomitings and purgings occurred.

Many who had been cured of cholera by the sedative and astringent treatment, recovered exceedingly slowly, and for several weeks after they had been pronounced well, suffered from vertigo, spasms, &c. &c.; but these symptoms quickly disappeared, when the discharge of bile was promoted.—*Revue Médicale.*

---

### XXIV.

### RHINOPLASTIC OPERATION.

THE patient had been long affected with an enormous cancer of the nose, which had resisted every treatment. M. Blandin, having completely excised the diseased part, detached from the forehead a flap of integument, and shaped it to the stump of the nose; but he did not divide the pedicle of the flap as has been usually done; instead of doing this, he separated the skin from it, and then cut away the integuments from the root of the nose; the opposite raw surfaces were brought together, and quickly united. By this manœuvre, the new nose continued to retain its communication with the blood-vessels and nerves, which imparted life to it at first, and was thereby much stronger and less likely to be affected by cold, and other accidents.—*Journal Hebdomad.*

---

### XXV.

### CURIOUS CASE, IN WHICH THE REAL SEX OF A CHILD WAS NOT DISCOVERED TILL HE WAS 18 YEARS OLD.

E. D——c. was born on the 13th Aug. 1812, and appeared to be a female, was registered as such, and educated as a girl till 1831. In June of this year she consulted M. Gendrin on account of ophthalmia in both eyes. He was struck with the beard on her face, the bass tone of her voice, and the development of the pomum Adami. The catamenia had not appeared. Subsequently she mentioned that there was an irregularity in the genital organs, and that the irregularity had been increasing ever since the age of puberty, which with her commenced about six years before, as indicated by the change of voice, and the black, stiff hair which appeared in different parts of the body. The mother stated that her child when young had the activity, and had always preferred the sports of boys, to the quieter amusements of girls.

She has not yet shewn any attachment for either sex. On examining the genital organs, a longitudinal fissure, like a valve, extended from the inferior edge of the symphisis pubis to near the anus. Rather higher up than the usual situation of the clitoris, was observed a loose cylindrical organ, 15 lines long and about five or six thick, covered by a very fine and extensible membrane, and ending in a regularly formed glans, having a corona round its base. The vena dorsalis penis was seen on its upper surface. A prepuce covered one half of its surface; the skin, of which this prepuce was formed, was continuous with the margins of the vulva-like fissure in the perineum. Along the under surface of this organ was a deep channel, or groove, which terminated posteriorly in the vulva, and at this point of junction were observed 5 open, irregularly arranged orifices, not unlike those of the vasa efferentia round the verumontanum. A smooth mucous membrane covered the surface of this gutter, which was in lieu of a urethra, and it presented faint traces of the longitudinal furrows observed in the natural passage. With much difficulty the finger could be introduced, to the depth of three inches, into the vulva, but no opening was detected in it. A straight instrument might be passed in the direction, from the symphysis pubis to the middle of the sacrum, for three inches and a half; and when the finger was carried up the rectum, the instrument might be felt, and it was ascertained that there was not more than two or three lines thickness of substance interposed. Two elastic roundish bodies were felt by the finger round the supposed neck of the bladder; these were probably the lobes of the prostate. No meatus urinarius could be perceived; but, as the water was discharged downwards, the orifice must have been situated in the perineal canal. At first, this canal was considered to be merely a dilated urethra, but the circumstance of the straight instrument being stopped at the depth of three inches and a half, and not appearing to be embraced by a tight canal, contradicted this idea. By perseverance, however, a curved instrument, directed upwards, was passed into the bladder, and the finger in the rectum did not discover any intervening substance, but the mere walls of the gut and bladder—a thickness of four lines at the most. There was no trace of scrotum, testicle, or of spermatic cord. The mammæ were not at all developed. The young person declared that the cylindrical organ, or imperfect penis, was sometimes enlarged and erected, and the erections had occasionally been followed by a discharge of a whitish, thick, viscid fluid. The rami of the pubes and the tubera ischii were more approximated than usual; the hips projecting; the buttocks flattened, and the thighs not rounded, and verging inwards as in females. The mons veneris, the linea alba, the chest, chin and cheeks, and the extremities were covered with hair, and the general configuration of the body was decidedly masculine.

The medical men, after a most careful examination, concluded that the sex of the person had been mistaken, and the civil tribunal, therefore, ordered that the registry should be altered, and the surname changed to that of a male. —*Revue Médicale.*

---

### XXVI.

### Delirium Tremens, simulating Drunkenness.

A man, 25 years of age, of very dissipated habits, who had been long affected with syphilitic symptoms, was suddenly seized with a strange inability to walk with steadiness. When he tries to advance, he reels sometimes to the right side, sometimes to the left, and cannot, by any effort, prevent it—the street, he says, is not wide enough for him. His pulse intermits at every seventh beat; all the other functions healthy. Opium, in substance, was administered, the dose being gradually increased from one to twelve grains a-day, in the course of three weeks. He was completely cured.—*Archives Génerales.*

---

### XXVII.

### Probable Cause of Extra-Uterine Pregnancy.

Madame E. on the 26th Feb. 1817, was suddenly much terrified, *while receiving the embraces of her husband,* by the fall of a stone which had been thrown against the window of her bed-room. She had no family hitherto, though she had been married for 12 years. The catamenia had regularly appeared till after this date, when the other symptoms of pregnancy presented themselves. On the 13th April, she had a violent attack of bearing down, and of excruciating pain in the left side of the hypogastrium, which very closely simulated the threatening of an abortion. The symptoms gradually passed by, but again returned in two months. On the 6th Dec. all the signs of child-birth came on; but the physician could not, by examining, reach the os uteri with his finger. After a few hours of suffering, all pains left her, and from this moment the motions of the child, which had hitherto been very obvious, altogether ceased. For three months after this date, she had repeated attacks of inflammation in the abdomen, and was reduced to a state of marasmus, when, very unexpectedly, the catamenia reappeared and health was quickly re-established. The belly, however, remained very large. After two years of widowhood she again married, and enjoyed good health till April, 1824, when she died from an attack of peritonitis, followed by diarrhœa. On opening the abdomen, the fœtor was insupportable, and M. Beclard, therefore, contented himself by removing the tumor along with the uterus, to which it adhered. The fœtus was of the female sex and of the full size; it was enclosed in a bag, situated to the left of the womb, and was in a great measure converted into a fatty matter, like adipocire.—*Archives Générales.*

---

### XXVIII.

### TORSION OF THE ARTERIES AFTER SURGICAL OPERATIONS.

*Case* 1. A young man's leg was amputated by the circular incision. The anterior and posterior tibial, and the peroneal arteries, were twisted in the simple manner recommended by M. Amussat, without any previous laceration of the internal and middle tunics. The time occupied was much shorter than required for applying three ligatures; one of the tibial veins, which was bleeding profusely, was likewise twisted. There was an oozing, more serous than bloody, from the wound for several days. The wound did not heal favorably, as the patient's health was not good; in six weeks, however, the stump was quite cicatrized.

*Case* 2. A woman, aged 48, had her thigh amputated; the femoral artery, well detached from the accompanying nerve, was twisted—only one other vessel required torsion. On the fourth day the bandages were removed; they were dry and very little stained: the wound was quite healed in four weeks. This patient having subsequently died of phthisis, an opportunity was afforded of examining the vessels; the femoral artery was closed exteriorly by condensed cellular membrane, and plugged inwardly by a fibrinous white clot. Its walls and calibre were quite normal.

*Case* 3. A man, aged 52, was admitted into the hospital with an enormous diffused, aneurismatic swelling of the lower third of the thigh: amputation was performed at the upper third. The femoral artery was attempted to be twisted, but it gave way at the third turn of the hand, leaving a gaping orifice; a ligature was, therefore, applied—the muscular branches were, however, secured by torsion. For several days after the operation, there was an inconsiderable serous oozing from the wound, which, however, was completely cicatrized in about six weeks.

*Case* 4. A man, aged 64, had cancer of the penis, which required removal by the knife; five arteries were twisted. It was found much more difficult to detach these vessels from their connexions than in the previous operations. The wound healed within a month.

In addition to the above cases, several others are alluded to of amputations, in which complete union of the wound took place in 6, 8, 12, 15, and 18 days. —*Journ. Hebdom.*

---

### XXIX.

### LETTER ON CHOLERA, BY PROFESSOR DELPECH.

AN attentive examination of the bodies of those who have died of cholera, clearly demonstrates that its essential or proximate cause is an active and disorganising inflammation of the solar plexus, semilunar ganglia, and renal plexuses, which constitute the great central point of the ganglionic system; and this inflammation is sometimes propagated to the pneumo-gastric nerves, to the pneumo-cardiac plexus, and even to the medulla oblongata. Numerous other pathological appearances have been remarked; but none of these is uniform; and many are evidently the result of pernicious treatment. The chain of symptoms most beautifully corroborates this hypothesis; for all the organs which are most involved, are under the influence and control of the ganglionic nerves; namely, the liver, the circulating and respiratory apparatuses, the abdominal viscera, including the kidneys and bladder. A curious fact is, that the vomitings and purgings consist of the serum of the blood, which being thus deprived of its watery portion, becomes thicker and thicker;—now this change cannot be conceived to take place without an important lesion of the tri-splanchnic nerve. The nature of this lesion we have explained to be an inflammation, and we should therefore infer that an antiphlogistic treatment must be most successful; practice has confirmed the truth of the conjecture: bleeding is the sheet-anchor of safety, when the severe symptoms have set in; the earlier stages may be combated with opium, warm baths, and strict abstinence. When collapse has taken place, we must employ stimulants, by giving them both inwardly and by rubbing them on the skin, or, what is much better, by injecting a diffusible one, such as camphor, into a vein, with the view of rousing the circulation, to enable us to draw more blood.—*Journ. Hebdom.*

---

### XXX.

### Permanent Contraction of the Fingers, cured by dividing the Palmar Aponeurosis.

Dupuytren has now performed this operation with complete success in upwards of twelve cases; and very lately he had a most satisfactory opportunity of confirming his opinion of the cause of this malady by dissection. An old woman died in the hospital; and it was found that three of the fingers were contracted. The skin of the hand and of the fore-arm having been very carefully dissected off, the aponeurosis was observed very distinctly to be drawn together. Wishing to shew that the distortion of the fingers was not at all dependent on any lesion of the tendons, Dupuytren divided them across, one by one; but yet the fingers could not be straightened: a direction was now carefully introduced beneath the palmar fascia, and this divided; and immediately the fingers could be bent back to their natural position.—*Journ. Hebdom.*

---

### XXXI.

### Disease of the Heart.

A man, aged 70, was admitted into the Hôpital de la Pitié, with the following symptoms—great emaciation, somewhat masked by a universal puffiness of the skin; complexion of a purplish hue; œdema of the ancles; dyspnœa; mucous râle; expectoration mucous and copious. Chest sounds well on percussion, except at the posterior and superior half on the left side, and at the præcordial region;—in these parts no respiratory noise to be heard; contractions of the heart feeble; a bruit de soufflet heard, extending to nearly the right clavicle. The pulse is rather small, 90 in the minute, and is slightly intermittent. The bellows sound is not perceptible in the arterial trunk, and is much more distinct in the points corresponding to the left, than to the right cavities of the heart. For five months the sleep has been much disturbed; the patient frequently awaking from frightful dreams—considerable orthopnœa.

*Diagnosis.* Hypertrophy of the heart, with contraction of the aortic and left auriculo-ventricular orifices, produced by induration of the valves.

Bleeding, laxatives, and digitalis were ordered. For a fortnight he experienced considerable relief; but soon after died, having been suddenly seized with a severe convulsive cough and bloody expectoration.

*Autopsy.* A large quantity of water was found in the cavity of the chest;—inferior lobe of the right lung completely hepatized; when cut into a sanious matter flowed out. The left lung is partly atrophied, being compressed by the heart, which lies transversely, and occupies a great part of the left side of the thorax. The pericardium contains a bloody serum; its inner surface is villous and unequal; where it is reflected over the heart, it is mamillated, red, and presents several small depositions of coagulated blood, and of false membrane, which form a thin easily separable coating, or layer.

The left ventricle, towards its base, is nearly an inch thick; columnæ carneæ much enlarged; its cavity of the size of a goose's egg; aortic orifice is contracted by cartilaginous incrustations; and the contraction is more obvious, in consequence of the increased capacity of the ventricle. The right ventricle is equally hypertrophied, and its walls are at least five lines in thickness, towards the base. Both auricles are dilated proportionally to the augmented size of the ventricles. All the cavities of the heart are filled with blood, partly liquid, partly in clots. The auriculo-ventricular orifices, and likewise the orifice of the pulmonary artery, are quite pervious and free;—only a few trifling incrustations are to be observed on their valves, and also on different points of the aorta. The weight of the heart, when all the large vessels are detached from it, is nearly one pound and three quarters, (800 grammes); its size is double that of a man's fist.

*Reflections.* This case is interesting on several accounts. The walls of the right ventricle are five lines thick;—Laennec never saw them to exceed four lines. The frequent inflammatory attacks on the lungs are probably referable to the blood having been driven into the pulmonary vessels with greater force; the bloody sputa, and copious expectoration, owed their origin to the same cause. The present case also proves that in a progressive enlargement of the heart this organ, by pressing on the inner surface of the pericardium, may induce a chronic inflammation, the pericarditis being thus probably consecutive to the hypertrophy of the heart; since there is reason to suppose that this latter affection commenced during the patient's youth. In other cases, we know that pericarditis will ultimately induce enlargements of the heart, just in the same way that chronic cystitis and gastritis will in course of time cause the muscular coats of the bladder and stomach to be amazingly thickened.—*Journ. Hebdom.*

---

### XXXII.

### Caution to the Public, or Hints on Scarlet Fever.

The author of this brochure appears to be gifted with qualities somewhat akin to those of the late Lord Castlereagh, of whom it was said, that his chief merit consisted in maintaining an immoveable equanimity of temper, and in being able to discourse on any topic for an hour or two at a time, and when he had finished, none of his hearers could say what he had been speaking about. So it is with our unknown Durham friend; he has been anxious to make a book, and in this he has succeeded, for we have got no fewer than upwards of 150 pages of well-printed letter-press. Why, then, should he have concealed his name? and why should he voluntarily deprive himself of the "summi honores" of authorship—of seeing his name in print?

The sum and substance of the book is, that scarlet fever is infectious (who doubted it?)—that its virus is peculiar (who thought otherwise?)—and absolute (pray explain what is the meaning of an absolute virus?)—and that it is specifically infectious in its mildest, as well as in its most malignant form; an assertion which will not be contradicted. The author does not deem it worth his while to establish the truth of the above positions by any facts or observations drawn from experience; but vehemently commands conviction, by very copious snatches from multitudinous books. The readers, who, by the bye, are assumed to be chiefly the non-professional public, are referred to Heberden, Willan, Binns, Rosenstein, Maclean, Paley, Bacon, Herschel, Salt, Adams, Jameson, and a host of others: they are favoured with a few poetical extracts on metaphysical subjects, and their attention is particularly directed to the analogy and close affinity between the cholera morbus (that prolific parent of octavos and duodecimos) and those swellings about the anus vulgarly denominated "piles," or "emerods" in the language of the Old Testament.

We hope that none who peruse this sage Durham work may be, as we confess that we are, sitting in the shadow of darkness: we have strained our minds to cracking, in order that we might be able to comprehend the said analogy; but we have hitherto woefully failed; yet how can we refuse to yield our credence when such mighty men as Bishops Hall, Warburton, and Wilson; Ostervald, Pyle, Bryant, Lucian, and that most surprising author Gyraldus "de Miscellaneis Deis" are adduced to vouch for the correctness of the opinion!! Our motive for alluding to this very curious disquisition is that we are afraid that very few, if any, may ever chance to see, and if they see, to have the taste to read, our Durham friend's book. Moreover cholera is too interesting a subject to be passed over; our author's opinion is at least quite new, viz. that this scourge is probably the same as is described in the 1st Book of Samuel, wherein it is written, "And he destroyed them, and smote them with emerods, even Ashdod and the coasts thereof," "and ye shall make images of your emerods that mar the land." It may be observed that the affinity of the two diseases is made more striking by the very curious fulfilment of the command; as may be witnessed in the devices of the caricaturists of the present day, representing cholera, at one time as a "blue devil," and at another as an "emblem of starvation," for no other purpose, than to fright the land from its propriety, and to seize upon and maltreat the doctors.

But this allusion to the cholera is only incidental; it is one of the many subjects which the author, in the plenitude of his riches, touches upon and embellishes with his great learning. The trunk and main branches of the treatise bear the fruit of his opinions on scarlet fever, which we are informed was "one of those judicial visitations which were imposed upon the Egyptians in the time of Pharoah," and which has devastated the different countries of the world ever since. The section on the treatment informs us that at one time the lancet has been recommended, at another time, cinchona bark; and that the author highly approves of the cold affusion. But we have not leisure, or patience to extract any more common-place statements. We are verily of opinion that the marrow of the book might be put into a couple, or three pages. The author is no doubt a young man, at least in composition: his style, as well as the meagreness of novel matter, betrays him;—strained, verbose and grandiloquent, on a subject that requires only a homely and concise narrative.

He must in future guard against this error, and not introduce any similes about inundations and torrents to illustrate the course of an epidemic!! "Res, non verba," are wanted in the present day.

---

### XXXIII.

### Deficiency of the Cerebellum.

M. Combette has communicated a very extraordinary case, in which the cerebellum, the crura cerebelli, and pons Varolii were altogether wanting. The patient, a young girl, was 11 years old when she died.—*Archives Géner.*

---

### XXXIV.

### Organization of Paganini!!

At a late "seance" of the Royal Academy of Sciences at Paris, the sapient auditory were entertained by a memoir on the above subject.

Dr. Bennati thinks that the modern Orpheus owes his excellency, not so much to practice, as to an original peculiarity in the organization of his outer man; he tells us that all the machinery of his arms is so beautifully pliant and moveable, that Nature evidently intended him for a great fiddler! Moreover, the trumpets of his ears are marvellously adapted for the reception of sound!!

His cerebellum is unusually large; (indicating his love for music we suppose!!) In short, ends Dr. Bennati, Paganini is an inimitable violiniste] by the necessity of his corporeal structure!!!—*Archiv. Géner.*

XXXV.

Pathology of Epilepsy.

Mary L. aged 61, had been subject to epileptic fits, ever since her childhood. At six years of age, she lost the perfect use of the left arm and leg. Menstruation commenced at the usual period, but the frequency of the fits did not abate.

On dissection, the parietal portion of the dura mater on the right side, was converted into hard bone. The right hemisphere of the brain was nearly one half smaller than the left; the right lateral ventricle so much dilated, and full of water, that the cerebral substance forming the walls, was only a few lines thick; the thalamus opticus wasted on this side; the left hemisphere was nearly quite normal; the corresponding ventricle of its natural smallness, and the thalamus and corpus striatum healthy. The frontal and parietal bones of the right side were a great deal thicker than those of the left.—*Archives Géner.*

XXXVI.

Curious anomaly in the distribution of the Bloodvessels.

The infant was born with imperforate anus, and lived for seven days. Dissection shewed that the rectum terminated in a cul de sac, about an inch above the os coccygis, which was but imperfectly developed. From the arch of the aorta, was given off, first, a trunk common to the two carotids; next the left subclavian; and lastly the right subclavian arising from the extremity of the arch, and then crossing to the right side, between the œsophagus and the vertebræ; the abdominal aorta having supplied the cœliac, superior mesenteric, right renal (the left was wanting, and likewise the kidney of this side) and spermatic vessels, divided into two branches on the second lumbar vertebræ. One of these, by far the largest, and appearing to be the continuation of the trunk, gave rise to the inferior mesenteric, and then crossed to the posterior surface of the bladder, along the median line of which, it ran to the umbilicus, where it divided into two. The other branch situated behind the former, first followed the course of the sacrum, then was reflected upwards to the right sacro-iliac synchondrosis, having first supplied branches to the left lower extremity, and left side of the pelvis. The continuation of this vessel, formed the right femoral. The system of the sympathetic nerves appeared to follow the irregularities of the arterial distribution—there being only one sacral ganglion, and no coccygeal.

M. Jodin, who has reported this case, is of opinion that the imperfect developement of the rectum was dependent on and caused by the imperfect development of the pelvic bloodvessels, and very properly directs the attention of medical men to examine whether the connexion always exists.—*Archives Génerales.*

XXXVII.

Retention of Urine caused by a Foreign Body in the Rectum.

A man aged 67, had suffered for several months from tenesmus and strangury, the urine dribbling away very slowly, and the desire to void it being very frequent. Many surgeons had attempted, but in vain, to sound him, with the view of curing a supposed stricture. On examining the rectum with the finger, a hard substance was discovered, pressing by one of its ends on the urethra; by the other on the coccyx; when removed, it was found to be the bone of a partridge, which, from the patient's account, must have lodged there for two months at least. He was immediately restored to health.—*Archives Génerales.*

---

### XXXVIII.

### Inflammation of the Medullary Substance in Long Bones.

This accident is by no means unfrequent after amputations of the extremities; and is of a most serious, and too often of a fatal result. If we examine the stump, we observe fungous granulations of a dirty grey colour from the divided surface of the bone; these are tender, if touched by the probe, for the membrane which lines the medullary canal is always inflamed in such a case. The morbid changes going on speedily effect the health of the bone itself; the vitality of which depends much more on its supply of blood from within, than from the periosteum outwardly. This is well illustrated by the difference in the results of injuries of the periosteum, from those induced by any lesion of the marrow and its membranes. In the former case, the depth of a thin shell, or exfoliation of bone is the extent of subsequent mischief; but in the latter, complete necrosis of the entire thickness of the bone is caused. Boyer relates an illustrative case. During the tedious healing of a stump, a cylindrical portion of the femur was thrown off spontaneously: the cause of this had been the ignorance of the dresser, who had every day been poking his probe into the medullary canal of the bone, mistaking it for a sinus. The patient ultimately recovered.

The dura mater appears to supply the place of the medulla to the cranial bones; for any serious injury, which causes its separation from the internal lamella, is speedily followed by the death of the bone at that part.—*Archives Génerales.*

---

### XXXIX.

### Memoir on Chlorotic Diseases.

Dr. Bland thinks that medical men have always taken far too circumscribed a view of these diseases, by considering them simply as symptoms, or as the signal and result of amenorrhœa. "Do we not," says he, "obseive them at all periods of life, in the male, as well as in the female sex, occurring too, even although the catamenia are regular; disappearing by the use of proper remedies, although this discharge remains obstructed? The real and specific cause of chlorosis, under all its Protean forms, is a vicious and imperfect sanguification; the blood being defective in crassamentum and colouring matter, and in consequence becoming less capable of imparting functional energy to the body. Four weighty reasons are adduced in proof of this doctrine. 1. Chlorotic maladies are almost always brought on either by whatever interferes with, or deranges the assimilation of the food and its conversion into the "pabulum sanguinis," as by living on unwholesome, and innutritious food, or by breathing a corrupted atmosphere, &c.; or secondly, by whatever enfeebles the system of the ganglionic nerves, which, we know, regulate, and keep in health the organ destined to form and to circulate the blood; such as all depressing emotions of the mind, masturbation, excess of venery, sedentery employments, &c. 2. The doughy, waxy whiteness of the skin, the pale lips and gums, the scanty and serous discharges from the vagina, nose, &c. and the watery state of the blood when drawn, all indicate the real nature of the disease, whose progress, 3d, is denoted by an utter want of power and activity in the organic functions of the body, arising no doubt from a deterioration of the fluid, wherein, it is said, that life resides. 4th. The efficacy of steel medicines, which have the power of restoring to the blood the "excitative" properties which it has lost, and which chiefly depend on its colouring matter.

So varied and so unsteady is the occurrence of symptoms in chlorosis, that it is almost impossible to define its characters within a single description. Sometimes the "anæmial" state of the skin, with slight general languor, are its only obvious characters; in some cases is added a lingering and wasting fever, which is not unfrequently attributed to visceral disease; in other examples, are intractable gastrodynia, not to be relieved by ordinary remedies; an asthma which defies all antispasmodics; a general tumefaction of the abdomen, and anasarcous state of the lower limbs; a restlessness and want of sleep, with, or without excruciating headaches, and murmuring noises in the head, against which depletion and counter-irritants are so commonly and so perniciously prescribed; or lastly, symptoms of diseased heart, which equally defy, what Hahnemann designates "antipathic" treatment, are a few out of the many ills and grievances, which have their time, seat, and origin in defective arterialization of the blood.

Dr. Bland, in very strongly recommending the different preparations of iron in chlorosis, does not assume any merit of discovery; he is well aware that it has been long the medicine in highest repute; but he very justly alludes to its not unfrequent want of efficacy; and is inclined to attribute this to the timidity with which it has been administered, and the improper forms, ill adapted to be received into the system, which have been employed. His favorite formula is thus—

℞. Ferri sulphatis,

Potassæ subcarbon. āā, ℥ss

Misce; in pilulas 48 dividend:

The dose at first is a pill night and morning, to be encreased gradually in a fortnight to 4 pills every morning, noon and evening.

Among the earliest marks of amendment is the return of colour to the cheeks and lips, and of animation to the eyes; the gastrodynia, want of appetite, sleeplessness, headaches, &c. are quickly much mitigated, or quite disappear. The breathing becomes easier, the pulse less weak and frequent, the strength increases, the anasarca of the limbs abates, and to cheerfulness of mind is added the feeling of bodily comfort and "bien-être."

From the long catalogue of cases enumerated in support of the author's treatment we shall select the following, one in a female, and two in male patients.

1. A. M. aged 21, had been remarkably pale ever since her birth; but the dirty waxen hue of the skin had increased for the last three years. The catamenia were regular, but very scanty and exceedingly light coloured. The health, however, was tolerably good; and neither the appetite nor the plumpness had decayed. By taking the steel pills in augmented doses for a month, she obtained bloom on her cheeks, lustre in her eyes, and vermilion in her menses!!

2. A. S. aged 57, had laboured under diarrhœa for eighteen months. He was excessively weak, and had a constant pain at the epigastrium. The skin, lips, and inside of the mouth were pale and exsanguine; pulse slightly febrile; no organic lesion of the abdominal viscera to be detected: the diarrhœa was checked by opiates; and the steel pills were afterwards continued for six weeks; the patient was restored to strength and health.

3. A. L., 27 years, had suffered from dysentery and ague during the late expedition to Algiers. His skin was blanched, his strength was utterly gone, his feet swelled at night; he suffered from oppressed breathing, and palpitations of the heart, and his sleep was uncertain and disturbed with dreams. No organic mischief was suspected, and therefore the symptoms were deemed chlorotic; the diagnosis was proved correct by the speedy cure under the use of the steel medicine.

It will be observed that the author very properly mentions in all the cases, that there was no organic disease; at least search was made, and none found; for it would be altogether a most dangerous and improper practice that steel should be administered in every case of disease which was attended with pallor of the surface, and of the mucous membranes, with muscular weakness, and bodily and mental depression, without reference to any other malady which might be co-existent. Every experienced physician knows that these symptoms are every day witnessed in uterine cancers, in chronic gastro-enteritic affections, in indurated liver and spleen, &c.; and neither steel, nor any other medicine can minister to such diseased systems, the bloom, and strength, and activity of health! But, in uncomplicated chlorosis, long experience has taught him to regard almost as a specific, the combination of the sulphate of iron and subcarbonate of potass.—*Revue Médicale.*

---

### XL.

### Historical and Critical Remarks on the Means employed to stop Hæmorrhage after Amputations.

M. Velpeau prefers the forceps to the tenaculum for laying hold of and drawing out the artery; and it appears that the latter instrument is very seldom used in France.

He states that the main artery of a limb is sometimes so hard and calcareous, that it will crack, like glass, if a single small ligature is applied, as recommended by English surgeons; the better plan, in such cases, is to previously introduce a cylinder of linen, cork, or elastic gum into the vessel, or to employ the "rouleau de Scarpa," by interposing something between the artery and the thread.

The medullary arteries of the bone will sometimes continue to ooze out for a considerable time; pressure with lint for one or two minutes will generally arrest it. M. Velpeau does not approve of the practice of delaying to close the wound for some hours after the operation, in the view of guarding against hemorrhage from the small vessels, a practice which has been adopted by Dupuytren, Klein, and others. It appears that M. Koch, surgeon of the hospital at Munich, has never used a ligature after any amputation for the last 20 years:—he trusts to graduated compresses on the artery, and to a bandage rolled firmly from the trunk to the wound, which is then to be immediately closed. From 13 experiments performed on dogs, the inference drawn was, that if any foreign body is introduced into the divided end of an artery, the vessel speedily takes on a morbid condition, which renders it incapable of receiving blood, although it is not mechanically obliterated. Another hæmostatic method, and one which is less objectionable, is to pull the bleeding vessel out, and then twist it a little back, or in a direction contrary to its course; in excision of the mamma, the arteries may be easily secured in this manner.

M. Velpeau has tried torsion of the vessels after operations, and has found it to succeed very well. He states that, whenever the artery could be easily laid hold of and drawn out, so that it might be separated from the surrounding tissues, and retained either by the thumb and finger, or by a second forceps, while, with the other forceps, it could be twisted from three to eight times round on its axis, the chance of secondary hæmorrhage was not greater than if a ligature had been applied; and that he has not met, in his practice, with the inflammations and suppurations which MM. Delpech and Lallemand have urged in objection. He thus compares the two methods of torsion and of ligature:—The former is not always quite so secure; it is not applicable in all cases; it requires very considerable practice to execute it well and effectively; and, lastly, it occupies more time in its performance than the application of ligatures requires. On the other hand, no foreign body is left in the wound, and it should, therefore, favor primary union; but we must acknowledge that experience has not yet confirmed and sanctioned the correctness of this presumption. The patients operated on by M. Amussat himself have not recovered more quickly than after the usual method; and a result more favourable has not yet been obtained by any of the numerous surgeons who have given torsion a fair trial. On the whole, M. Velpeau gives the preference to the ligature; but admits that, occasionally, especially in operations in soft yielding structures, the hæmorrhage may be securely checked by simply twisting the vessels.—*Revue Médicale.*

---

### XLI.

### Medical Journal of Brazil, published at Rio de Janeiro.

The first Number of the "Semanario de Sante Publica," or Weekly Journal of Public Health, has recently issued from the press at Rio, and augurs well of the zeal and observation of the medical men there. 'Tis, indeed, refreshing to the mind, to mark the benign influence which liberty diffuses over the intellectual, as well as over the moral energies of mankind; for, while the parent countries of Spain and Portugal are oppressed with despotism and sunk in apathy and indolence, their children of the New World are animated with a generous rivalry of scientific distinction. France, before the first revolution, had only one medical journal of any importance, and now she is the most prolific mother of periodicals. We shall extract one or two short articles.

#### I. Hepatitis, ending in an Abscess, which was discharged outwardly.

A. M. aged 20, had a smart attack of acute inflammation of the left lobe of the liver, which gave way to antiphlogistic treatment; but, although the febrile symptoms vanished, some tenderness of the epigastrium and right hypochondrium remained, which, in a few days, was followed by a soft, fluctuating swelling. In 15 days, the abscess was ripe for discharge, and it was, therefore, opened by the lancet. (N.B. The better method is that recommended by Dr. Recamier; viz. to employ caustic instead of a cutting instrument, for thus the adhesion of the cyst with the abdominal parietes is rendered more secure, and all danger of effusion prevented.) The suppuration was encouraged for a few days, and soon after, under simple dressings, the cure was completed.

#### II. Purulent Cysts in the Cavities of the Heart.

A. C. aged 21, had long suffered from indolent ulcerations of the legs, and presented a most cachectic appearance. He had symptoms of hydrothorax, and of diseased liver.

On dissection, the left lung was found completely wasted, and the left cavity of the pleura filled with a flocculent, serous fluid. The heart was enlarged, and when its cavities were laid open, numerous purulent cysts, varying in size from that of a millet-seed to a hazel-nut, were observed to line the surface of the left ventricle and auricle.—*Revue Médicale.*

---

### XLII.

### Statistical Researches on the Increase of Population in Europe.

M. Moreau de Jonnés states that the prolific powers of the human race are such, that from every marriage we may estimate, on an average, the birth of six children, of which two die usually in infancy, and the remaining four survive, marry in their turns, and become thus multiplying nuclei of population. To exhibit the ratio of increase in figures—the offspring of one couple is supposed to be 6 children in 33 years, 12 in 66 years, 24 in a century, 192 in 200 years, more than 98,000 in 500 years, and upwards of three thousand millions in 1000 years. According to this estimate, provided there was no impediment to the natural order and course of events, and no obstacle to propagation, a single family, living at the time of Charlemagne, might have peopled the whole of the inhabited world.

The experience of nations, however, gives a very different account of the increase of man in any given country:

thus Gaul, at the time of the Romans, contained about four millions of inhabitants, and it has required no fewer than 1860 years to raise the population to its present census of 32 millions; instead therefore of a couple doubling its numbers in 33 years, as above stated, 615 years, or about 18 times as long a period has elapsed, before such was effected; a calculation which supposes that the annual excess of births over deaths, is only one in a thousand.

If, as we have reason to believe, the population of the world does not exceed a thousand millions, it follows that it has doubled itself only 28 times since the period of the deluge; and that the average interval between each duplication has been 150 years.

The following is supposed to exhibit an accurate view of the progressive increase of population in the different countries of Europe.

| | Years. |
|---|---|
| In Prussia the population has been doubled in | 39 |
| In Austria | 44 |
| In European Russia | 48 |
| In Poland and Denmark | 50 |
| In Britain | 52 |
| In Sweden, Norway, Switzerland and Portugal | 56 |
| In Spain | 62 |
| In Italy | 68 |
| In Greece and Turkey | 70 |
| In the Netherlands | 84 |
| In Germany | 120 |
| In France | 125 |

From this table it appears, that if we group together the Northern countries of Europe, the average period taken to double their population is scarcely half a century; whereas it requires about 80 years for this purpose in the southern countries; the mean of both is nearly 57 years. We must observe that the term indicated in the table which we have just given is to be viewed only as an analytical census of the fecundity of mankind in different countries; and not as a correct announcement of the number of inhabitants which will be found at any particular date occupying such and such a country.—*Revue Médicale.*

### XLIII.

### Nutritious Soup prepared from Bones.

M. Cosmeny states that, in consequence of the events of July, 1830, the working classes in Rheims were reduced to great want, and means had to be taken to afford relief to upwards of 4,200 families in extreme indigence. An assessment of 23,000 francs was therefore laid on the inhabitants. M. Cosmeny had the direction of the whole. He established an immense apparatus for preparing soup from the boiling of bones; and in 133 days he distributed 301,910 rations, as follows. There were 212,800 rations of broth (potage) and bread; 35,000 rations of ragôuts made of potatoes with the fat of bones; and 26,600 rations of boiled meat; and lastly, 9,310 rations for the support of the cooks who were employed in the kitchen. The expense of each ration, including the charge for the apparatus, he estimates at 7¼ cents; the quantity of bones used every day was 480 pounds, which yielded 600 rations of soup, 18 ounces to each ration.—*Revue Médicale.*

### XLIV.

### Contagion.

Dr. Esquirol gives it as his opinion that all contagious diseases near the period of their decline, lose the power of propagating themselves; and he illustrates the truth of the remark, by the curious fact, that towards the termination of an epidemic variola, the matter of the pustules may very frequently be inoculated without effect; and that, as a general rule, all pestilences are more destructive and fatal when the miasmata are yet in small quantity, than when they afterwards become more diffused, and consequently more diluted.—*Revue Médicale.*

### XLV.

### DILATATION OF THE CAVITIES OF THE HEART.

CORVISART and all preceding physicians supposed that the invariable, and the only cause of dilatation of any of the cavities of the heart, was some mechanical impediment to the exit of the blood from them. MM. Louis, Bouillaud, Andral, and Pigeaux have, however, clearly shewn that this explanation will not always hold good. The cavity most frequently dilated is the right ventricle, and yet of all the openings communicating with the heart, that of the pulmonary artery is the least often found diseased; hence we may safely conclude, that there is no direct connexion or dependence of these two morbid states, the one upon the other. We must therefore search for a cause more general; and we shall probably be led to discover it in some change of the muscular and moving powers of the walls of the affected cavity. M. Pigeaux has observed that the right side of the heart is generally dilated in those who have died of asphyxia; and that the left ventricle when dilated, is usually hypertrophied at the same time; and that these morbid states most frequently co-exist, especially in old persons, with a diminished firmness and consistence of the substance of the heart, and with an ossification of the arteries of the trunk, or extremities. M. Piorry, who was appointed by the Academy of Medicine to report on M. Pigeaux' Memoir, thinks that an increase in the size of the right side of the heart is very often dependent on the circulation through the lungs being obstructed by tubercles, by hepatization, or by simple asthma, by fits of coughing, or by the efforts of vomiting. It is of great importance, therefore, in reflecting upon the causes which may give rise to dilatations of the right ventricle and auricle, to consider well, whether the pulmonary circulation has probably been exposed to an occasional or to a permanent interruption, or impediment of any sort; but M. Pigeaux deserves well of medicine in having established his opinion that dilatations of the heart are much less frequently dependent on some contraction of its orifices than has been generally believed. —*Revue Médicale.*

---

### XLVI.

### VARICOSE TUMOUR IN A VENOUS BRANCH COMMUNICATING BETWEEN THE TWO JUGULARS.

A MAN, aged 23, entered the Hôpital de la Charité under the care of M. Roux. There was a tumour situated immediately above the clavicle, of the size of a pigeon's egg, which was at first supposed to be an encysted tumour;—it was firm, elastic, and did not pulsate. The patient had noticed it for about two years, but it was only within the last two months that it had given him any annoyance. Its true nature was not ascertained before the operation; when cut into, a quantity of coagulated black blood flowed out, and the swelling was now discovered to be formed by the dilated vein. M. Roux put a ligature round it, and also around several thoracic branches. Fortunately the wound healed perfectly in a fortnight. In his clinical lecture upon this case, the surgeon remarked that varicose tumours of the veins of the superior part of the body are very rare; he had seen one case, under the care of Sir A. Cooper, in which the superficial veins of the fore-arm were immensely distended; and another had been mentioned to him very analogous to the present example; the walls of the internal jugular vein were found perforated with numerous small holes, and the swelling had been formed by the effusion of blood into the surrounding cellular tissue. These varicose tumours have a great analogy with the aneurism which Mr. Pott once observed in the neighbourhood of the popliteal artery, and which had been produced by the blood escaping from small perforations in the sides of the artery. Morgagni has seen the vena azygos equal the size of the superior cava in a case, wherein it had been ruptured and had caused the death of the patient by the effusion of blood into the right cavity of the pleura. Mr. Cline once met with a case of varix of the internal jugular, which burst and occasioned death by hæmorrhage. — *Journ. Hebdom.*

---

XLVII.

Tumor—the remains of an old Hernial Sac in the Sheath of the Spermatic Cord.

A man, aged 29, had a tumor in the spermatic cord, situated between the inguinal ring and the testicle, of the size of a walnut. It has been there for 15 years and given little annoyance till three months ago, when it began to increase in size and to become more painful. When carefully examined, it conveys to the finger the sensation of an enclosed fluid; the ring, cord, and testicle are not involved with it. The patient states that at first the swelling might be pushed up to, but never within, the original canal. It was considered to be either an encysted tumor of the cord, or the remains of an omental hernia, or a fleshy or scirrhous tumour. M. Roux extirpated it. On puncturing it, a quantity of pale yellow serum flowed out. Death, from fever, took place a fortnight after the operation, and on dissecting the inguinal canal, the omentum was found adhering all round to its circumference. M. Roux gave it as his opinion that the tumour which was removed, had been an old hernial sac, the open neck of which had been in course of time obliterated, and stated, that what tended to confirm this was, that during the operation it was found to be very much more coherent at its upper than at its lower part.—*Journ. Hebdom.*

---

XLVIII.

Dissection of a Person who died from Inanition.

A prisoner at Toulouse died after having refused to take food for 63 days. Universal emaciation; weight of the body about 58 pounds. Brain of a paler colour than natural; no water in the ventricles; cortical substance of healthy consistence; medullary substance of the cerebrum, cerebellum, and the medulla oblongata considerably firmer and denser than is usually observed. Heart of the ordinary size, but very pale in colour; soft and easily torn; lungs nearly normal; œsophagus contracted; stomach of its ordinary size, and containing an ounce or two of greenish fluid; mucous membrane adhering strongly; the thickness of the walls of the stomach and intestines sensibly diminished; valvulæ conniventes very distinct; hardened fæcal matter in the right portion of the colon; the rest of the intestines empty. The omentum reduced to a mere serous web, which was traversed by blood-vessels. Mesentery containing no fat. Liver of a healthy colour and size; its substance denser than usual. Gall-bladder distended with a black, thick bile, which was very like a strong solution of extract of liquorice. Spleen exceedingly small, almost round, with a diameter of about two inches; texture very dense and resisting pressure. Kidneys reduced in size, and of very firm consistence. Muscular system almost "annihilated," so shrivelled were the fleshy "ropes of life." Though no trace of fat was to be observed, the marrow was not deficient in the long bones.—*Archives Géner.*

---

XLIX.

Eruption of Roseola caused by the Use of Copaiba and Cubebs.

A young man was ordered by M. Velpeau a mixture containing the balsam of copaiba, powder of cubebs and magnesia: he took it for six days, when he began to experience troublesome itching and smarting on the neck, chest, &c. &c. and next morning there was a full eruption of roseolous patches over his body. These patches very much resembled the rash of measles. By discontinuing the medicine, and keeping the body cool, the young man was well in a day or two.—*Archives Générales.*

---

### L.

### CASE OF LITHOTRITY.

BERENGER, aged 16, had laboured for many months under all the symptoms of calculus in the bladder, the presence of which was recognised by the sound. The instrument recommended by M. Civiale was used. It was introduced without difficulty, and only very slight pain was felt by the attempts to lay hold of the stone, whose diameter was two centimetres (nine lines.) When it was properly secured, the operator began to work the borer, by which the calculus was soon crushed to small pieces, and on withdrawing the instrument, it was found filled with the debris. The operation lasted only seven minutes. The patient was ordered a warm-bath, and while he was in it, he passed per urethram many calculous fragments, some of them being of the size of a pea, and causing considerable pain and smarting in the passage. After a few days quiet, a sound was introduced but no vestige of any calculus could be found; and since the operation the patient continued well. This is one of the very few cases in which lithotrity has succeeded in curing a person at one sitting.—*Archives Générales.*

---

### LI.

### OBSERVATIONS ON A SPECIES OF APOPLEXY, IN WHICH THE BLOOD IS EFFUSED IN NUMEROUS POINTS ON THE SURFACE OF THE BRAIN. By M. DANCE.

A YOUNG woman, aged 21, complained at first of a shivering, and of feeling as if she had been well bruised and beaten. In a day or two the symptoms of fever shewed themselves, but yet there was no fixed pain in the head. She was bled and kept on low diet. On the following day there was greater prostration; the pulse was weaker; she complained of pain all over her body, and did not refer it to any particular inward part; her arms, however, if moved gave her much distress and even sharp pain, like that of rheumatism; and when she was provoked by questions, she began to acknowledge that she felt an uneasiness in her head. On the following day slight delirium and general restlessness, with great prostration, drowsiness, inability to protrude the tongue; a purplish red hue of the face; heat and slight moisture of the skin; frequent, full, but feeble pulse; an occasional cough; an almost complete paralysis of the upper extremities; when they were raised, they fell down if not supported, and no effort was made by the patient; the head if elevated rolled down again on the pillow when the hand was withdrawn; the urine was passed involuntarily; she became quite comatose, and died next day.

*Dissection.* No serous effusion under any part of the arachnoid. At the posterior third of the left hemisphere near to the course of the longitudinal sinus was observed a patch of deep red colour, quite circumscribed, and of about the size of a shilling; the cerebral substance to the depth of two or three lines was involved in this patch, which appeared to be produced by a softening and breaking-down of the substance of the brain, and by being intimately blended with effused blood. When a small stream of water was allowed to play on this patch, an irregular excavation was left, the cerebral matter being washed away; the surrounding substance appeared quite healthy. A few lines distant from this spot, another not larger than the head of a large pin was noticed; and when examined attentively, it was discovered to be a minute clot of blood. On other places of the surface of this hemisphere were many more of these black points, in every respect like the former, and all presenting the mixed characters of ramollissement of the brain, and of hæmorrhage, as if small clots of blood had been infiltrated into the softened cerebral matter. This appearance was most conspicuous at the lower and back part of the hemisphere where it rested on the tentorium. On the right hemisphere, and also on the cerebellum, one or two similar dark-coloured spots were to be seen. The medullary substance of the brain, the ventricles, thalami, &c. were healthy; vessels not gorged. No other morbid appearances in the head, thorax, or abdomen.

*Case* 2. A woman, aged 25, entered the Hospice Cochin on the 9th of May. For six weeks previously she had laboured under dropsical swellings of the limbs; and latterly there was general anasarca. After shivering, followed by heat, slight delirium, and drowsiness, symptoms of more decided cerebral oppression supervened; the patient would not or was unable to answer questions; she did not appear to suffer from pain; the heat of skin was somewhat increased, and the pulse was more frequent than in health; tongue natural; abdomen soft and yielding.

Next day the stupor was more complete; but yet she could be roused to open her eyelids and answer questions, though indistinctly; the limbs have lost all spontaneous motion. On the 11th, symptoms worse; dilatation of the pupil; breathing laborious, as if the muscles of respiration had lost their power. The patient died, soon after, comatose.

*Dissection.* The surface of the brain and its investing membranes appeared quite healthy; but, on separating the hemispheres from each other, the corpus callosum was seen to be studded with minute red points; it looked as if any white surface had been sprinkled with red paint. On examining these attentively, it was speedily found that they were not produced by simple rupture and effusion, for pressure on the surrounding substance had no effect in squeezing out any oozing of blood; and there was no vestige of clots; their true nature was an intimate blending of the medullary substance of the corpus callosum with the colouring matter of the blood. From their minuteness, it was not very easy to ascertain whether ramollissement, or any alteration of the cerebral matter, was existent or not. With the exception of a few scattered red spots on the left thalamus opticus, similar to those just described, no other morbid appearance was detected any where.

*Remarks.* The attack in both cases has been sudden, and the progress rapidly fatal, unlike the instantaneous shock of apoplexy, arising from simple effusion. The embarrassment of speech, the lethargy and coma, increased from day to day, and death soon following to a state of general exhaustion and insensibility, are peculiar. The pathological appearances are to be considered as a sort of diffused capillary hæmorrhage, or circumscribed effusions of blood, which irritate and disturb the cerebral functions, instead of overpowering and oppressing them, as in ordinary apoplexy. Each of the bloody points described above is to be considered as a miniature of the extravasations which we observe so frequently contained in fissures or irregular cavities of the cerebral substance, after apoplectic and paralytic seizures. It is worthy of remark, that both of the narrated cases occurred in young persons, whose bloodvessels are more resisting than in the later periods of life. M. Cruveilhier has alluded to this form of disease, in the article "Apoplexia," in the Dict. Pratiq. de Medicine.—*Archives Génerales.*

---

### LII.

### Chronic Hydrocephalus cured by puncturing.

A child, aged four months, presented a well-marked case of this disease; the largest circumference of the cranium was 18¼ inches; the fluctuation was very perceptible at the fontanelles; for when pressure was made upon one, the other was distended and forced out. M. Graefe, compressing the larger fontanelle, plunged a large cataract-needle into the smaller one to the depth of the third of an inch. A little viscid fluid escaped drop by drop; he, therefore, introduced a very fine trocar, and immediately a transparent yellowish-brown liquid flowed out freely—uniform pressure was made on the head as the water escaped. When an ounce and a half had been evacuated, the infant began to faint and became extremely languid; the canula, was, therefore, removed, and a firm bandage put round the head. Stimulants were given, but the child was restless and ill for two days. A similar train of symptoms followed each operation, which was repeated generally at an interval of 12 or 14 days, and sometimes between two and three ounces were discharged at a time; the size of the head sensibly diminished, and the general health improved. The puncturing was repeated eleven times in the year 1829, on the following days—8th, 15th, and 23d of January, 19th February, 5th and 19th of March, 19th and 27th of April, 5th and 17th of May, 23d of June. The evacuated fluid was thicker and more coagulable towards the end of the cure. The smaller fontanelle and the sutures in course of time closed; the child became healthy and strong. When 10 months old it began to walk; and on the 26th of November, 1830, the child, aged 2½ years, was presented well and active to the Society of Medicine at Berlin.—*Graefe and Walther's Journ. der Chir.*

---

### LIII.

### THE ADVANTAGES OF REVULSIVE BLEEDING IN AFFECTIONS OF THE HEAD.

FORMERLY, in the schools of medicine, it was much disputed whether bleeding, local and general, had or had not a revulsive power; that is, of drawing the blood from other parts to that part whence the blood was allowed to flow. This doctrine includes the idea of derivation as well as of revulsion; and when the terms are applied to bloodletting, we mean by the former the abstraction of blood from the diseased part itself, and, by the latter, from a part situated at a distance. M. Chauffard, the author of these observations, states that he has often seen an inflammatory affection of the head aggravated by leeches to the temples or behind the ears, and relieved at once by drawing blood from the feet or ankles; he mentions several cases of acute ophthalmia, unchecked and uncured by local bleedings from the neighbourhood of the eyes, speedily disappear by the application of leeches to the lower extremities. The good effects are much enhanced by warm hip or foot baths; cold applications at the same time kept constantly applied to the head, and large doses of calomel. The following is an instructive example.

A young child had a very severe ophthalmia, which was treated by leeches to the temples, blisters to the nape of the neck, &c. The symptoms, however, did not abate, and the formation of pus in the chambers of the aqueous humour was the consequence. M. Chauffard ordered leeches to the ankles, followed by hot poultices, fomentations to the abdomen, a general warm-bath, while cold was applied to the head, and a dose of 15 grains of calomel twice a week. The pus was gradually absorbed, and vision restored.

As a general rule, our author states that, when a local bleeding near the seat of the disease does not at once relieve the symptoms by the evacuation of blood, and the depression thereby induced, the symptoms are always made worse. Otitis is uniformly most speedily cured by leeches to the anus, vulva, &c. or by opening the saphæna vein; cynanche will yield to one general bleeding, and will frequently resist a dozen applications of leeches to the throat. A hemicrania is often more severe after leeching the temples, and yet vanishes by a hot pediluvium; and every one knows that a neuralgia of the face is generally exasperated by local bleeding, for if the pain does not yield soon after the blood is drawn, the "engorgement fluxionnaire" of the cellular substance which surrounds the nervous twigs is increased, and the pain in consequence is agonizing. The excruciating headaches which succeed to a stoppage of the catamenia, are best relieved by the application of leeches to the vulva; an attack of coup-de-soleil, by opening the saphæna vein; the acute hydrocephalus of children by the same means.

We shall illustrate this by narrating a case.

A child, a year and a half old, was labouring under the symptoms of hydrocephalic disease. Pulse small, irregular, and threadlike; dilatation of the pupil; occasional flushes of the face; confirmed coma and insensibility to pain, occurring after an attack of hot fever, with severe headache. Three leeches were ordered to each ankle, and the leech-bites to be covered with hot poultices for several hours. The little patient revived somewhat, and, on the following day, the symptoms were more favourable, and the infant gradually recovered.—*Archives Générales.*

*Remark.* We direct our readers' attention to the above remarks, as illustrative of the good effects of revulsive bleeding in many diseases, but especially in those of the head. We are aware that many may and will object to M. Chauffard's opinion of its superior efficacy, and allege that local bleeding near the affected part would have probably been as successful, in the cases whose cures he ascribes to the revulsion of the blood to a distant part. It is a practice not common in this country, though much followed abroad, and, we are of opinion, with exceeding good effects, especially in those cases of cerebral disease dependant on an obstruction of any accustomed discharge.

---

### LIV.

### Account of Cholera in Paris.

The disease in Paris, as in all other places, has committed far greater proportional ravages among the poor and ill-fed, than among those in more comfortable circumstances. Males have suffered much more from this scourge than females, and the aged and intemperate than the active and abstemious. Most of the victims have passed 30 years; still, however, many children and young pregnant women have fallen sacrifices. It has been remarked that in most, but certainly not in all, there were the premonitory symptoms of diarrhœa or some irregularity of the bowels, before the violence of the choleric attack. The seizure, in almost every case, occurred between midnight and four or five o'clock in the morning. The usual course of the symptoms has been as follows:—Borborygmi, uneasiness and pain of the bowels, followed by watery or whey-like stools, ejected by frequent squirts, without any effort, colic, or tenesmus; then succeed the watery vomitings and cramps; but these last have by no means been constantly present. Most patients have complained of a distressing pain at the epigastrium, increased by pressure, and frequently extending over all the belly; urine very scanty; restlessness; extraordinary prostration, almost to the annihilation of vitality, even although the evacuations were not very profuse; feebleness of the voice; cold of the whole surface and of the tongue; pulse feebly or not at all perceptible; face shrivelled and blue. Most of the patients who were brought to the hospitals, after having been 10 or 12 hours ill, were cold, pulseless, and cadaverised. An instance has scarcely been known of a recovery, after the pulse had ceased at the wrist.

The pathognomonic phenomena have been generally, great internal congestion; the stomach and intestines have been uniformly found abnormal in some respects, exhibiting either numerous red injected patches, or large ecchymoses; the secretory follicles were always enlarged. No feculent matter was ever found in the intestines; but, in its stead, the rice or barley-water-like liquid, which was sometimes tinged of a brown or reddish colour in those cases in which the intestines were ecchymosed. The blood had probably exuded and mingled itself with the watery secretion. In one or two, not the slightest trace of disease could be found on dissection. The urinary bladder was always contracted. No lesion has hitherto been remarked of the nervous plexuses and ganglia in the abdomen.

*Treatment.* The employment of stimulants has generally been followed in the stage of prostration and cold, but not with much success. M. Recamier and some other physicians have used the cold affusion as a stimulant; and it is reported that this method has had better effects in restoring warmth and circulation, than frictions and external heat. The above short sketch is taken from the Archives Génerales de Medecine for March; and we shall now lay before our readers, a more particular and circumstantial report of the disease, as it has been observed in the different leading hospitals of the French metropolis; and first we select one, or two cases from the Clinique of M. Broussais.

*Case* 1. F. Belleval a soldier, was brought to the Hôpital du Val de Grace at 9 P. M. on the 31st March. Extremities cold and livid, &c.; pulse thread-like; tongue red; intense thirst. He had been seized five hours before with cold shiverings; severe pains in the head, borborygmi, cramps, vomiting, but no purging—there was great prostration and stupor; but intellect unclouded. Treatment—dry frictions; 20 leeches to the mastoid processes, and 40 to the epigastrium; iced water to drink. The patient expressed himself relieved, in proportion as the leeches fastened. On the following day all the dangerous symptoms had vanished, and he gradually recovered.

*Remarks.* Though the above case may not be deemed one of concentrated cholera, it is worthy of being studied in connexion with the treatment pursued. If not the disease itself, it is only one degree of kinship removed from it.

*Case* 2 *of cholera, or choleroid disease occurring at the same hospital, a month before it invaded Paris as an epidemic.*

A man, aged 29 years, presented most of the symptoms of cholera: his extremities cold; his aspect cadaverous; racking and unintermitting pains of the bowels; borborygmi, profuse diarrhœa, and severe vomiting of a yellowish green matter—great tenderness of the epigastrium much encreased by pressure—pulse small, frequent and intermitting: skin cold; great thirst; very considerable exhaustion. Leeches were ordered to the epigastrium, and blood was drawn from the arm—the vomitings and diarrhœa soon ceased; and the pain was much mitigated; and the other symptoms gradually gave way.—*Hôpital de la Pitie.*

---

### LV.

### OBSERVATIONS AND EXPERIMENTS ON RESPIRATION.

THERE is a condition of the parts composing the organ of respiration, a knowledge of the influence of which on the function, must very materially modify the prevailing theory concerning it. This condition is the permanent patency of the air-passages and cells. It pervades every portion of the passages. The mouth nose and larynx having bony walls it cannot be denied to them; the trachea and main trunks of the bronchi being occupied by cartilaginous rings, must also have it awarded to them; but as the cartilaginous rings occur less and less frequently, the farther the tubes dip into the lungs, and, as at their terminations, none are to be found, so that the extremities of all the tubes are entirely membranous, their claim may be doubted. Yet it must be borne in mind that these tubes are strictly attached to the air-cells, and therefore must remain open as long as the cells continue so. It is well known that the cells are open after death; for they are found to contain a large quantity of air, at that period when the lungs are under the greatest degree of compression of which they are susceptible, whilst the thorax is entire and atmospheric pressure is excluded. They must be more expanded during life, to which state attention is at present solicited. In further proof of this point, it may be observed that, after death, when the utmost degree of compression which the ribs can permit exists, the tubes are not closed; for if the thorax be opened, the lungs shrink considerably, and air is expelled from the cells through the tubes, which it is clear could not be effected, were they closed.

This fact (namely, the ever-pervious state of the air-passages) established, the influence it holds on the function is next to be traced. It is identical with that of the tubes of an hydraulic engine, the common lifting pump for instance; in that, the tubes are ever full of water, and the water thrown up by the action of the piston, comes not immediately from the well, but was previously resident in the tube. In like manner, in the action of the organ before us, the air expelled by expiration comes not immediately from the cells, but is the contents of the passages. And, in inspiration, the atmospheric air drawn in descends not to the cells, but the air previously contained in the passages enters the cells, and the supply from the atmosphere takes its place in the passages. It is evident that all the air of an inspiration does not reach the cells, the seat of the function, and that if the capacity of the passages be equal to the bulk of an inspiration, none does. It is a question, then whether any, and if any, how much, is employed in the function?

To answer this question with full satisfaction, it is necessary to be in possession of the exact measure of the capacity of the passages, and of the bulk of an inspiration. Who shall presume to determine the amount of an inspiration, when men of so great physiological knowledge, are so discordant in their statements as they have been? In this dilemma, there is no alternative left, but to take the highest estimate which any one of them has given, and that is 56 cubic inches. It must then be enquired whether the capacity of the passages be equal to that?

The state of the lungs is ever varying; the tubes are always contracting and expanding; the time when the measurement is to be taken, is when an inspiration has just been completed, before expiration begins. Then the diaphragm is contracted, and the abdominal viscera pushed downwards; the ribs raised; and the thorax enlarged, the cartilaginous bronchi, incapable of diametral enlargement, are stretched longitudinally; the cartilaginous rings receding from each other, the membranous portions are both distended laterally, and drawn out lengthwise. The cavity of the thorax, thus expanded, is filled by the lungs, and throughout their substance the tubes ramify, from their root, at the termination of the trachea, to countless points on their whole circumference touching the boundary of the cavity. Their size, too, is not inconsiderable; dissection shows them several lines in diameter; and it must be admitted that, before they can be examined in this way, they have much contracted, and lost, at least, one fourth of their bulk. It will be seen to be impossible, contemplating the figure of the lungs so out of all order, and the distribution of the tubes so perplexed, to come at a precise valuation of their capacity. A probable one, is the utmost to be obtained. If it be granted that the bronchi, cartilaginous, are capable of containing 40 cubic inches (it is contended that this is a low computation) then the second position, viz. that none of the air of inspiration reaches the cells, is established; for no person will refuse 12 cubic inches of contents to the mouth and nose; and four to the larynx and trachea; making together 56, equivalent to the bulk of an inspiration, none of which, therefore, can pass beyond their limits.

Taking it for granted, because physiologists agree on this point, more than on any other in connexion with the subject, that inspiration and expiration are equal in bulk—that atmospheric air is an agent in the function—and, seeing that, in the functional process, some of the air taken in must necessarily be dissipated, an anomaly seems to present itself. It will be solved hereafter. At present, another difficulty claims attention, which is raised by the presence of carbonic acid gas, in mixture with the expired air, which it is said to receive from the blood; but whence it cannot receive it, if it enters not the cells. The following experiment will remove the difficulty.

Two long narrow glass jars, each containing 120 cubic inches were procured; a hole was drilled in the centre of the bottom of each; the bottoms ground plane. They were attached bottom to bottom, by means of gas lute, with the holes in apposition. A piece of wood, having one end tapered, as a plug for the holes, and long enough to extend without the mouth of the jar, was fixed in the holes. Two stopcocks were affixed to a bladder, one at the neck and the other at the fundus, so as to inclose a space capable of containing ten cubic inches. The bladder was filled with expired air expelled immediately after having made an ordinary inspiration; it was passed from thence into one of the jars; the operation was repeated till the jar was full; and its mouth was then stopped. Here was collected a quantity of air which had been inspired, and expired, each portion was the last taken in, and none of it had passed beyond the mouth and trachea, if it therefore contained carbonic acid, from some substances within these parts it must have obtained it. The empty jar was filled with transparent lime-water, the plug withdrawn, and the mouth stopper applied, the lime-water dropped slowly through the air, and the air rose in small bubbles through the lime-water, the jars were turned several times. At length the bottom holes were again stopped, fresh air and lime-water introduced into the jars in like manner as before, and the whole process renewed, taking care to retain all the chalk deposited. These operations were continued till a considerable quantity of chalk was collected.

This experiment affords proof that the carbon of expiration is not all received from the blood in the air-cells, but that a portion at least is derived from substances within the passages. So far it is conclusive, but it is desirable to find the substance which produces it, and the method by which it is generated; the following hypothesis is offered in explanation.

The only matter to be found in the passages is the mucus, poured into them by proper secretory organs. Physiologists say that all the fluids of the body contain salts in the alkaline state, except the mucus of these passages, in which they are in the neutral state. It is assumed for proof is not attainable, that this particular fluid is also sent from secretory organs having alkalies in solution, that having all favourable circumstances in attendance for the purpose, the oxygen of the atmospheric air is attracted by the alkali, that carbonic acid gas is disengaged by the new combination, mixes with the air contained in the passages, and is expired together with it. Whether this explanation be received or not, still it is no less certain that carbonic acid is generated in the passages, and that it can emanate from no other substance than the mucus thereof. A very large proportion of the bulk of the lungs consists of cells, their dimensions are enlarged by inspiration, and lessened by expiration, and the variation of dimension is in exact proportion to the amount of air taken in and expelled by these two acts; still they are in every state full of air.

It is in the cells that the function is exclusively performed; air is an agent in the function: the air which inhabits them must be that agent. The method by which this air is supplied, and its use in the function must be explained.

It has been supposed that the quantity of air expelled by expiration is the same as that taken in by inspiration, and taking it for granted that air is an agent in the function, and seeing that no air is dissipated in the operation thereof, an anomaly was suspected, for the universal order of nature, especially throughout the animal system is, that all matters in action suffer waste, but in the instance before us it seems to be interrupted, for if the air taken in is returned without diminution of quantity it loses nothing. If it be shown that the expelled air differs from the inspired only in quality though not in quantity, or that its bulk is increased by addition during its stay in the lungs the difficulty will be removed.

It is vulgarly known that the air or breath comes from the lungs loaded with a large quantity of vapour, it is true vapour is condensed as soon as it comes in contact with the external air, vapour which is only in simple mixture with the air of respiration, but vapour holds place in warm air in two ways, first by simple mixture, and secondly by chemical combination ; in the latter case the water is not separable from the air except by chemical decomposition. No circumstances can be conceived more favourable for this combination than those existing in the lungs, contact of steam and warm air, and pressure.

The thermometral temperature of breath was ascertained to be 76°. Temperature of room 62°. Temperature of water in the pneumatic trough was raised to 76°. A bladder capable of containing 30 cubic inches, having a stopcock at the neck and another at the fundus, was filled with atmospheric air, the nozzle of one stopcock closed was fixed in the mouth of the double jar, and the whole of the other taken into the operator's mouth. The air was inhaled into the lungs, immediately expelled into the bladder again, and thence passed into the jar; the jar was filled by repetitions of this process, and left with the under mouth open in the water. At the expiration of twenty-four hours 11 cubic inches of water had risen into the jar. Four drachms of caustic potash were put into the jar, and five inches more of water had entered in twelve hours. A change of weather afforded an opportunity of repeating the experiment with advantage. The temperature of the room was 72°, —of breath 82°,—in nine hours 12 cubic inches were condensed—caustic potash absorbed six more.

These experiments demonstrate what was clear enough before, that atmospheric air taken into the lungs is there increased in temperature, and therefore in space, and that it is further increased in quantity by addition of water in the state of vapour ; the necessary result of which must be, that as the same bulk of air is returned as was inhaled, some of that which had been inspired must be left behind, expiration being finished, and the quantity left must be exactly equal to the increase of bulk attained through increase of temperature, and through addition of vapour. Caloric expands, and vapour takes the place of atmospheric air so expanded, detaching a quantity proportionate to its own bulk, so that this amount of displaced air remains in the lungs after expiration is completed.

But it was conjectured that the foregoing experiments did not declare the full amount of air left behind, and the following confirmed the conjecture.

Three large bladders were procured, and two circular pieces of wood, grooved at the edge like the wheel of a ship's block, and perforated in the centre by a hole half an inch in diameter ; two bladders were attached to each piece, so as to maintain communication with each other by means of the holes. To the opposite extremity of one of the last bladders in the line a stop-cock was fastened. This apparatus was filled with atmospheric air amounting to 350 cubic inches. This air was drawn into and expelled from the lungs by 70 inspirations and as many expirations, one of the last succeeding each of the former, the whole was left in a cold cellar 24 hours, at the expiration of which time upwards of 100 cubic inches were found to have been lost, being about $1\frac{1}{2}$ cubic inch from each inspiration, and this under the influence of simple condensation alone ; the air still held water in chemical combination, and it will not be overlooked that if this had been removed by decomposition, the result would have been much more favourable.

The residual air then is supplied by the respiration, and a quantity equal to that lost is consumed by the function during the time occupied by every act of respiration.

Concerning the use of the residual air, it is concluded from the foregoing inquiry, that air having once entered the cells, never returns into the atmosphere, but on the contrary is constantly receiving accession from it ; indeed, were there no waste, and the same quantity were returned as had been received by the different acts of the organ, the residual air would ever be the same, unvarying in quantity, a portion of it would only change place, at expiration a portion sufficient to fill the space left by the air just expelled would rise from the cells into the passages, and at inspiration it would return to the cells, and make room for the atmospheric air that enters. This would be the mode of procedure if the capacity of the tubes and bulk of inspired air were equal, or if the latter exceeded the former, but if the capacity be inferior to the bulk, then a portion of air equal to the excess of bulk over capacity rises from the cells, and is thrown out of the mouth at expiration, and a like quantity of fresh air descends into the cells at inspiration, but the greater part of the contents of the cells moves to and fro as in the former case, the result would be scarcely different. Whichever then may be the state of the case, we cannot hesitate to admit that the residual air is the only inhabitant of the cells, and as it is universally known that the function is carried on in the cells and in no other part of the organ, the residual air must be regarded as the only agent in the process of renewal of the blood.

The air receives caloric in the passages, acquires much increase of temperature, and consequently much augmentation of bulk; the air expired, indeed, on account of its short stay, is not much affected, but the alimental portions taken from each inspiration and the residual gain the temperature of the body. A quantity of water in the state of vapour becomes mixed with it—another quantity of water becomes chemically combined with it—its oxygen is continually attracted by a substance residing there, and entering into new combinations—and carbonic acid gas is continually being generated, and mixing with it. All these changes are known to be in progress, because all expired air is increased in temperature—has water mixed—and in chemical combination—and exhibits loss of oxygen—and acquisition of carbonic acid. These changes are effected upon the air of inspiration during its momentary stay in the passages, and they must be more considerable upon the alimental and residual portions, which having once entered the passages never return. The alimental portion descends into the cells, and comes into action on the blood in this altered condition.

Two ox lungs were procured, they were cut into narrow strips in the pneumatic trough, under a glass jar, and as much air as could be expressed was collected. A large coffee-mill was fastened to a log of wood, this was inverted in the trough, each strip of lung passed through the mill, and the air set loose directed into the jar. Into a jar full of this air, ignited charcoal was introduced, it was speedily extinguished. Phosphorus would not burn in it. Lime-water was poured in, and shaken together with it; a copious precipitate of chalk took place. Into a jar containing another quantity of the air a solution of sulphuretted hydro-sulphuret of potash was poured, and after shaking, and opening the mouth of the jar under water, it was scarcely perceptible that any water rose in the jar. In the jar again filled with another quantity of the air deprived of carbonic acid by means of quick lime, caustic potash was suspended, and the mouth of the jar sunk in water, after a few hours much water had risen. Thus it was ascertained that the residual air consists of water in chemical combination with it,—of a large proportion of carbonic acid gas; perhaps it contains no oxygen, and if any, a very small portion. What other gases it contains was not discovered through want of acquaintance with chemical manipulations.

A summary of the facts thus produced is this. But let it be premised, that the tubes ramify in the lungs, and terminate at about an inch distance from each other on their surface,—that the cells are placed in rows parallel with them, and all around them,—that the cells in contact with the tubes communicate with them by means of pores in the sides of each,—and that the cells communicate with each other by like means.

In the ordinary process of respiration there is a pause, the moving powers rest after expiration has been finished, and before inspiration begins; in this state let the organ be contemplated: the cells and passages are all full of air. The muscles of inspiration are called into action — the cavity of the chest is enlarged—the cells are expanded—each cell is affected in an equal degree—the line of cells in the centre of the space between tube and tube attract air from the cells in contact and communication with them—these last also from others attached to them—each row in succession does the same —the last, or those in contact and communication with the tubes, attract from them—the membranous tubes expanded in circumference, and elongated attract from the cartilaginous bronchi—these enlarged in length attract from the trachea, mouth, nose and atmosphere.

This will be the course of procedure whatever may be the relative capacity of the parts, and the amount of air taken in. But in the succeeding act of inspiration it will be necessary to consider it in two different relative states of capacity, first as though the volume of air inspired exceeded the capacity of the passages, and secondly as though it was equal or inferior to it. Following then the progress of the function, bearing in mind that inspiration and expiration are supposed to take in and send out an equal volume, these will be found to be the mode of proceeding, and result under the first consideration. The muscles of expiration act, the air is propelled from all the parts in the order in which it entered them reversed, all the air contained in the passages is sent out through the mouth and nose, and as much from the cells also as is equal to the excess of bulk of inspiration over and above the capacity of the passages. Say, for instance, that the capacity of the passages is 30 cubic inches, and the bulk of inspiration 40, then ten cubic inches of air will rise from the cells and be thrown out of the body. Besides this portion of air, another will rise from the cells equal in magnitude to the capacity of the passages, and occupy them; the cells too will remain full of air. The inspired air during its stay in these parts gives away part of its oxygen, which uniting with carbon existing in the fluid contained in them, forms carbonic acid, the new compound being of equal bulk with the oxygen which disappears. The air obtains also a quantity of water in the state of vapour. This body of air so changed, and augmented by addition, is further swelled by the expansive quality of heat, to which it is here exposed. A quantity of air equal to the magnitude acquired by these means is left behind in the passages after expiration, and besides it will be found that expiration is not really equal to inspiration, consequently another portion equal to the excess of inspiration over expiration is also left behind. This quantity of air so changed, deprived of some of its original components, and increased by addition of other matters, remains at the top of the column, in the mouth and nose, at the end of expiration, at the succeeding inspiration descends again into the cells, again at expiration ascends, but now not so high as its former position, by as much space as it at first occupied, a fresh quantity occupying that which has taken the same course and undergone the same changes as itself; at each succeeding inspiration it descends deeper and deeper, and at each expiration rises less and less high, till it is spent in the object of the function. From the time of its entrance by the mouth, till it is wasted, it travels an incalculable number of times up and down the passages, and it must be plain that as the expired air undergoes so considerable a change during its momentary visit in the passages, this suffers an incomparably greater; perhaps it loses all its oxygen; it is known that it obtains carbonic acid, and a large quantity of water, in a state the most apt for using its affinities, and forming fresh unions. We know that the nitrogen returns unchanged and undiminished, but we do not know whether the nitrogen, carbonic acid and water, form any new compounds amongst themselves, or with other substances when they come into communication with the blood. The conjecture that they do carries with it much probability from the length of time they are returned in the passages and cells, subject to all the means of synthesis, and exposed to agitation, such a powerful auxiliary of the purpose.

It has been seen that in this first assumed state of relative capacity of the passages and bulk of air a portion of the air, equal to the excess of the last above the capacity of the other, will have entered the cells. It may be objected to the present hypothesis, that this portion receives all the carbonic acid, contained in expired air, from the blood, to which it is in them exposed, and that all the oxygen lost is there given to that fluid. To this objection, the first experiment is a full answer; and it may be stated, in corroboration, that the same fluid (mucus) is poured into the cells as is found in the passages; therefore the same change takes place in them as in the passages, by the same process, between the same substances.

If the second supposed state of parts exists, namely, if the magnitude of inspiration is equal or inferior to the capacity of the passages, then the same course of procedure will be observed, and the same result follow, as in the former case. This has been traced and pointed out above, except that none of the air will enter the cells or come in contact with the blood, and all the changes effected upon it will be wrought within the passages only.

This account of the result of the process has reference only to the air expelled by expiration; but in this second supposed case, as in the other, there is a portion left behind by each act of expiration, which has been designated alimental, because it maintains the residual, the most important, vitally important, part of the air of respiration, the only part which has influence in the function, none other coming into contact with the blood of the pulmonary artery. Each alimental portion takes the same course, and rises and sinks in the same manner in the passages in this as in the former, and undergoes the same changes; the only difference is, that whereas, in the first, some of the atmospheric air taken in by inspiration goes directly to the cells, and is there exposed to the blood, and is expelled into the atmosphere by the succeeding expiration; in the second, all the air taken in by inspiration is confined to the passages, and all, except the alimental portion, is again expelled by expiration, without coming in contact with the blood in the cells, the seat of the function.

---

### LVI.

### CASE OF TIC DOULOUREUX, PRODUCED BY A SCIRRHOUS TUMOUR ON THE BASE OF THE BRAIN. By JAMES HEYGATE, M.R.C.S.

MRS. B. aged 53, the lady of Capt. B. R.N. had been troubled some time with chronic inflammation in both eyes, and though the conjunctiva was considerably suffused with red blood, and lymph thrown out on the cornea, she felt no pain in the eyes. This inflammation reluctantly gave way to the usual remedies. She then complained of pain in the left side of the head, over the temple. This continued (with temporary relief from bleeding, blistering, &c.) for a few weeks, when the affection began to put on a more formidable appearance, and was marked with all the characteristic symptoms of that most painful disease "tic douloureux." Paroxysms of severe pain extended over the cheek bone, just below the orbit, the alæ of the nose and the upper lip, which was considerably drawn up, shewing the second branch of the fifth pair of nerves to be affected. The line of demarcation was most clearly drawn, the nerves of that side of the face being implicated only. The pain was not attended with any discoloration of skin, only numbness, and occasionally the cheek and temple were puffed. Pressure neither gave pain nor produced relief; severe fits of pain would sometimes reach the ear, which led me to suppose that the portio dura of the 7th pair of nerves was affected. The digestive organs, in the earlier part of the attack, were not deranged. Thus things went on, with nothing decidedly and unequivocally to lead one to suppose that there was organic disease at the base of the brain; but my suspicions gradually began to strengthen, when no real, or hardly temporary benefit, followed the use of the most approved remedies; such as, externally, counter-irritation by blisters, tincture of iodine,

&c.; internally, carbonate of iron in large doses, and for a long continuance. Then followed arsenic, quinine, and, in fact, the whole catalogue of medicines usually recommended; anodyne plasters applied down the face, with fomentations of like matters, together with opiates taken internally, gave the only perceptible relief.

After suffering for about four months in this way, it was perceived that the left eye-ball began, by degrees, to protrude, till it was pushed out to a very considerable extent. Whilst this was going on, the power of vision in the eye became completely lost; then followed, at first in a trifling degree, and then more and more profusely, an acrid discharge from the left nostril, of a very offensive nature. It was now clear that there must be ulceration; and, from the nature and seat of the pain, I concluded that this ulceration was where the nerves supplying the face and head make their exit from the brain. During this time, it was obvious enough that diseased action was extending its ravages; the glands on each side of the neck became much enlarged, and ultimately encroached on the œsophagus—so much so, as to render the passing of fluids only allowable.

After the discharge from the left nostril had continued, and increased in offensiveness, for about three months, bloody matter began to flow from the right nostril also, and the sight of the eye on that side gradually diminished. Thus things went on, from worse to worse—the tumour in the neck increasing. If asked about her head, she would say there was a feeling of torpor and heaviness, not to be described. The discharge of matter was occasionally very copious, and when it did not issue so freely from the nostrils, it passed into the pharynx; and, as swallowing was very difficult, it frequently produced a sense of suffocation; latterly, it seemed *insensibly* to pass into the stomach and through the bowels. She became extremely emaciated; and, after suffering the most excruciating agony with exemplary patience, and with only short intervals of ease, for upwards of a year, she was seized with a diarrhœa (which had frequently before threatened), and which now terminated a most deplorable state of existence.

*Post-mortem Examination of the Head.* On dividing the dura mater, and gradually lifting up the brain, I discovered a tumour at the base, about the size of a pullet's egg, resting partly upon the left crus cerebri and tuber annulare. On cutting into the tumour, it appeared of a hard, white, cartilaginous consistence; the surface of the tumour, on the fore-part, was ulcerated, as were the parts of the ethmoid and sphenoidal bones adjoining. The great pressure on the left optic nerve accounted for loss of sight; and ulceration had extended along the course of the optic nerve, and matter was seated behind the eye-ball: ulceration had extended itself to the right optic nerve and underneath; and this may account for the sight of that eye, in the latter part of the illness, giving way. The scalpel, through the ulcerated parts, passed very readily into the nostril and left eye-ball. A short time before her death, pieces of slough were removed occasionally from the back part of the mouth. There was more water in the ventricles than is usually found. No other deviations from health were observable in the brain.

Two or three conclusions may be drawn from this case:—1st, That it had some connexion with the catamenia, which continued till within two years of her death; and, as Mrs. B. had occasionally complained of a dull pain, and odd sensation above the left eye, probably this tumour might have begun to form some years, as, when well, she frequently expressed a wish to her maid that her head should be opened after her death, feeling convinced that there was something uncommon to be found there, from what she frequently felt; and wherever there seems to be a fixed determination (however slight at first) for diseased action to continue, our prognosis should be doubtful.

### LVII.

### MR. RAINEY ON ARSENIC.

*To the Editor of the Med.-Chir. Review.*

1, *Maze Pond, Borough.*

SIR, As I believe no process has hitherto been described by which the oxide of arsenic and antimony may be reduced to their metallic state by the agency of galvanism so as to afford an easy and satisfactory test of the presence of those metals in minute quantities, I have been induced to send you the particulars of some experiments which I lately made for the purpose of supplying this deficiency in toxicological chemistry. Should you consider these experiments sufficiently conclusive, and the subject one of interest enough to merit a place in your Journal, you will oblige me by inserting this letter in your next number.

If two or three grains of arsenious acid be dissolved in two drachms of strong liquor ammoniæ and six of water, and a drop of this solution mixed with about half as much liquor ammoniæ be put upon a piece of polished silver, and these brought in contact with a clean piece of zinc, and detained there for a minute or two, decomposition of the arsenious acid takes place; the oxygen appears to be attracted by the zinc, which is discoloured, while the metallic arsenic coats the silver. Nearly all that part of the silver which was covered by the solution, excepting the point of contact of the metals, receives a coating of arsenic: hence in the employment of this test for very minute quantities, the solution should be spread over as small a portion of the silver as possible. The colour of the arsenical coating varies a little according to the quantity of arsenic, but though the quantity may be ever so small, even the 3,000th part of a grain, the metallic lustre will be distinctly visible. After the reduction has been effected the excess of ammonia must be washed off immediately by immersing the silver in water, otherwise the arsenic will be dissolved. After all the ammonia has been removed the metallic arsenic will be found so firmly adherent to the silver, as to be with difficulty separated. If a drop of pure aqua ammoniæ be put upon the arsenical coating, it will gradually dissolve the arsenic, and this solution placed upon a piece of glass may be made to exhibit the presence of this metal when tested by the nitrate of silver or sulphate of copper. It will also exhale the alliaceous odour if collected in a sufficient quantity and ignited with charcoal. A very weak solution of tartrate of antimony may be tested in the same manner. The metallic coating which this salt imparts to the silver may be easily distinguished from arsenic both by its colour, and more satisfactorily by the easy solubility of the latter in liquor ammoniæ, which does not affect the former. The decomposition of the sulphate of iron and copper, the muriate of cobalt, nitrate of bismuth, the nitro-muriate of platinum, and probably several other metallic oxides, can be effected by means of a similar employment of galvanism; but these which have been named differ from antimony and arsenic in not requiring the aid of ammonia to decompose them. The manner in which ammonia contributes to assist the decomposition is probably by yielding hydrogen to the oxygen of the arsenic, and perhaps too by acting slightly upon the zinc, it increases the galvanic action of the metals. Minute bubbles of gas are apparent about the point of contact of the zinc and silver, which from their remaining involved by the solution may be presumed to be nitrogen. To ensure the efficacy of this test, the silver, and more especially the zinc, must be kept perfectly clean and bright, otherwise the latter covered with a small portion of arsenic from a former experiment, when brought in contact with the silver might give it a coating of metallic arsenic, and lead to mistake or uncertainty. If the arsenic acid has been previously mixed with tea, broth, or porter, it still suffers decomposition on the application of the foregoing test, though in a slightly diminished degree, unless the quantity of arsenic be greater than that already named. G. RAINEY.

*May* 23, 1832.

P.S. The arsenical coating may very conveniently be tested by the addition of a small quantity of slightly diluted nitric acid to it, and afterwards dissipating the moisture by the heat of a spirit lamp. The acid, when first applied, detaches the arsenic, which soon becomes oxydized and combining with the silver, coats it with a greenish-yellow crust. As a piece of coin was employed in this experiment, the green colour may be attributed to the copper which had been mixed with the silver.

---

### LVIII.

### Drs. Vivenot, Sen. and Jun. on the Cholera of Vienna.

In our last number we published the letter of Dr. Yates (see page 607), accompanying the translation of a paper by the above-mentioned eminent physicians of the Austrian capital—a paper that was first solicited by the Board of Health here, and afterwards refused insertion in the Cholera Gazette, because the writers were non-contagionists! We regret that the press of matter, and the lapse of time prevents us from giving more than a short extract from this interesting document, to shew that the physicians of Paris were not the only people who denied contagion in cholera on the Continent.

"The observations which chiefly lead us to the conclusion that the disease is not contagious, are as follow:

1. Without having recourse to any kind of preservative, having left the house fasting, we exposed ourselves freely and unreservedly to the influence of the disease at a time when it was at its highest; and, during the first days of the most violent raging of the disease, we came in the most perfect and nearest possible contact with the patients, and even had our faces and clothes soiled by the rejected contents of their stomachs; and then we would often remain fatigued, exhausted, and deprived of sleep for days together, but still in the enjoyment of the best of health up to the present hour.

2. By reason of our very numerous avocations, and the hurry and pressure of business, we were prevented either cleaning our clothes, washing with vinegar; or having recourse to any other similar precaution, after having visited the sick on any one occasion; and yet we do not know of a single instance in which the disease had been carried from the Cholera Hospital (No. 2 in the town, the care of which was intrusted to my father, Dr. Vivenot) into private practice, either by himself or me, who inhabited the same house, and had, moreover, the charge of a district of the town, containing a population of 14,000 persons. We seldom found that nurses (and especially those whose duty led them to attend on cholera patients) were attacked by the disease; and when it did so happen, it could generally be traced to other causes, to wit, improper diet, previous indisposition, timidity, anxiety, or other contending emotions, which predispose to, and are themselves exciting causes of, the disease.

3. Locking up and seclusion did not prevent the spreading of the disease. On the contrary, those persons who did so, and imagined themselves safest, frequently fell a sacrifice within the very walls of their supposed asylum, in consequence of an anxious separation from their friends and relatives; and were so much the more certain of the epidemic, as they were the victims of their *fears*.

4. The falling sick of cholera of several individuals of a family, or of various persons in one and the same house, depends upon, and is to be attributed to, sorrow, anxiety, or fright, which all excite the disease; a circumstance which by no means speaks for the contagious nature of the epidemic."

A perusal of the paper from the pens of the Doctors Vivenot has impressed us with a high opinion of their talents and judgment, as physicians and philanthropists.

---

### LIX.

### Broussais' Lectures on Cholera.

It was to be expected that the numerous disciples of Broussais would press their master for some declaration of opinion and practice, on such a destructive epidemic as that which has lately scourged the French capital. Accordingly the professor of the Val de Grace has delivered two lectures on cholera, that have attracted considerable attention on the Continent, and even here, where we had the advantage of priority in observation. We can only abbreviate the professor's chief ideas.

I. M. Broussais believes that the disease has reigned in Europe at former periods (à d'autres époques), and that it is the same epidemic which, in the fifteenth century, was called the "black plague." The professor seems puzzled how it should have travelled from India by way of Russia, instead of coming by a much quicker journey with the English ships and commerce! In Paris, there was an avant-courier of cholera, namely, a morbid sensibility ("une grande susceptibilité intestinale") of the alimentary canal.

II. "How has this scourge been generated, or how has it got amongst us? Its invasion has been too sudden to account for it by the introduction of a germ, a veritable contagion, passing from the infected to the healthy. Nevertheless, as there is generally more than one person affected in each house—as several people usually take the disease in succession—as the epidemy spreads, as it were, by chance or caprice, through towns and villages, under conditions the most opposite, and under circumstances the most dissimilar—sparing some and scourging others, we are compelled to confess that there is something mysterious and unknown in the mode of its propagation."

III. The predisposing causes were numerous. Among the chief were derangements of the digestive organs—preceding diarrhœa and other affections of the intestinal canal—*fear*, of whose influence M. Broussais cites some curious illustrations. The Neapolitan Ambassador had studied the charts day and night, watching the course and predicting the irruptions of cholera. Among other predictions was one, that he himself would fall an early victim to the contagion of cholera! It may be fairly presumed that in this, as in many other instances, the prophecy fulfilled itself! All excesses, moral as well as physical, predisposed to the epidemic.

M. Broussais considers the disease as varying in its phenomena, according to three different portions of the alimentary canal on which it happens to make its first impression. The first portion extends from the mouth to the termination of the duodenum—the second consists of the small—and the third of the great intestine. The last is most frequently the seat of cholera—the first is least frequently attacked—and still more rarely are all three invaded at the same time. When the colon is affected, there are slight colics and abundant evacuations, by each of which the patient feels relieved; but, after a time, the evacuations change their appearance, becoming white, very thin, flocculent and characteristic of cholera. There is no bile. When the small intestines are first affected, there are borborygmi, distention of the abdomen, and ultimately diarrhœa. When the stomach and duodenum are primarily affected, there is constipation instead of diarrhœa, nausea, vomiting, at first without pain, but afterwards with progressive suffering, cramps in the lower extremities, and a kind of trismus of the lower jaw.

In some cases the disease first attacks the nervous system, and the patient faints, or becomes prostrated in an instant, with vertigo, but with no pain, no gastric or intestinal disturbance. Vomiting or diarrhœa, however, do not fail to succeed sooner or later.

The lining membrane of the alimentary canal has been found more inflamed and disorganized, according to M. Broussais, than we have found it here. The consequence has been, that the celebrated professor of the Val de Grace has pronounced the disease to be highly inflammatory action of the whole alimentary canal—and that it is extremely rapid in its course. If we do not always find the mucous membrane red and injected, he thinks it is owing to the quantity of watery fluid thrown out, by which the membrane becomes blanched.

The treatment pursued by M. Broussais may be easily imagined. Ice was given to the patients almost ad libitum —leeches were applied to the epigastrium—and heat to the extremities. By these means, it is said that he lost about 1 in 6 at the beginning of the epidemic —and only 1 in 40 towards the decline of the same. This, if true, is strongly in favour of the antiphlogistic treatment —for few physicians can boast of such success.

In respect to prophylaxis, the professor recommends a reduction of one-half of our food—the avoidance of all sorts of vegetables and fruits—of all sorts of stimulants, watchings, fatigues, and excesses of every description. He strongly advises people of timid disposition or weak nerves to avoid the sight of choleric patients. There can be little doubt that tens of thousands have become affected with cholera, during the reigning epidemic, through fear. Of all exciting and predisposing causes, this is the most powerful.

---

### LX.

### Anthelmintic Emulsion.

Turpentine is now regarded as the most certain of our medicines for the expulsion of intestinal worms: it has been exhibited under a great diversity of forms, with a view chiefly to the modification of its nauseousness, or the increase of it specific powers: as combined in the subjoined prescription, it acts with remarkable efficiency.

℞. Infusi Sennæ, f. ℥x.

Syrupi Rhamni, f. ℥j.

Confectionis Scammoniæ, ℈ij.

Copaibæ, ♏xxx.

Olei Terebinthinæ Rectificati, f. ℥vi.

Ritè misceantur ut fiat emulsio, quæ horâ matutinâ sumatur.

This is the dose for an *adult*, having no contra-indicative symptoms: the patient should take it in bed, about four or five o'clock in the morning, and afterwards endeavour to sleep. Generally, within four hours, the medicine begins to determine cathartic effects: if these do not then commence, a small teaspoonful of the confection of scammony, or half an ounce of castor oil, will induce alvine evacuations; after each of these, some drops of an essential oil, or of the aromatic spirit of ammonia, or a little brandy in water, will counteract the tendency to squeamishness and exhaustion which the terebinthinate medicines usually produce. In cases where invermination is indicated by general symptoms, though worms have not been discovered in the patient's dejections, this medicine often accomplishes the dislodgment of these reptiles and a removal of the disorder which their presence had occasioned: in all other cases, the results of its administration are invariable and salutary. For the destruction of tæniæ, the emulsion should be exhibited at least four times, at moderate intervals; its repetition, within a fortnight, seldom fails of eradicating the other kinds of parasitic animals by which the alvine canal is infected. The use of this vermifuge, should be accompanied and followed by a course of medication with bitters, iron, and mild resinous aperients with aromatics.

---

### LXI.

### Preternatural Lactation.*

Aristotle gave immortality to the he-goat of Lemnos, when he affirmed, for the benefit of after-ages, that this generous animal yielded a profusion of milk, from which the goatherd made excellent cheese. Very useful too, in his way, was "the wether which suckled a lamb;" and the history of this phenomenon is a contribution to philosophy,† from the pen of Dr. Doddridge, alike distinguished for his sagacity, and erudition, and piety.

Donati,‡ in his "Marvellous His-

---

* Communicated by Dr. Kennedy of Ashby-de-la-Zouch, Leicestershire.

† Philosophical Transactions, Vol. XLIV, for 1746-7; Phil. Trans. abridged, Vol IX, p. 557.

‡ Marcello Donati; De Medicâ History;" Paullini,* in his second "Century;" and the Bishop of Cork,† relate intances of the lacteous secretion being abundant in men.

Cardani‡ enumerates the circumstances of a young woman, who had never been impregnated, whose breasts yielded milk, on their being excited with the stings of nettles.

Dr. Stack,§ and Dr. Montègre,|| have detailed cases of copious lactation, accidentally re-produced in *aged* women: to these the present writer will add some account of a woman who gave suck to children, from the *twenty-fifth* uninterruptedly to the *seventy-second* year of her age; this person is still alive and accessible to examination.

I. *Dr. Stack's History.* Elizabeth Brian was examined by Dr. S. and another gentleman, at her house in Tottenham-court, in 1733: she was then in the sixty-eighth year of her age; and for more than 20 years she had not given birth to a child. Her breasts were "full, fair, and void of wrinkles; but her face was very much withered, her cheeks and mouth vastly sunk in, her eyes red and running with a clammy humour." Upon pressing her right breast at the Doctor's desire, "she fairly squeezed out milk which gathered in small drops at three of the lactiferous ducts terminating at the nipple." Having himself carefully dried the nipple with his handkerchief, Dr. S. made her repeat the experiment, and it had the same result. About four years previous to this examination, E. B.'s daughter, being obliged to be absent for a considerable time, left a babe she was then suckling, in the care of its grandmother: the old woman "finding the child froward for want of the breast, applied it to her own, barely in order to quiet the infant, without the least thoughts of milk." This having been repeated several times, a son of E. B.'s, now grown a man, perceiving that the young one swallowed something from the nipple, "begged leave of his mother to try if she had not milk: his experiment succeeded, and he drew milk out of the same breast from which he had been weaned above twenty years; for seventeen or eighteen years previously his mother had not given suck to any child. Two years afterwards, E. B.'s daughter had another babe; whereupon its grandmother weaned the first, and commenced suckling the new-born infant. "In my presence," says the Dr., "this infant took the nipple with as much eagerness and seeming delight as I ever perceived in a child of two years old; and at it plainly performed the actions of suction and deglutition: the two children, both girls, are, as to constitution, such as I could wish to the dearest friend; plump, and firm in flesh; in complexion, cleanly, fair, and healthy; and in temper, brisk and sprightly, considering the mean diet of their nurse. When this good woman came to town," the Dr. adds, "which was near two years since, her milk abounded to that degree in both breasts,

---

toriâ Mirabili, opus variâ lectione repertum. Lib. VI, Cap. 2. 4to. Venetiis, 1588.

* Chr. Fr. Paullini; Observationum Medico-Physicarum Centuriæ IV. Francoforti, 1706.

† Philosophical Transactions, Vol. XLI, p. 813; for 1739-1741. Phil. Trans, abridged, Vol. IX, Part III, p. 208. Medical Essays and Observations, abridged from the Philosophical Transactions, by S. Mikles, M.D. Vol. II, p. 426, 1745.

‡ Girolamo Cardani; De Rerum Varietate Libri Septendecim, Lib. VIII, Cap. 43. Basileæ, 1557.

§ Thomas Stack, M.D. Philosophical Transactions, Vol. XLI, for 1739-1741, p. 140. Phil. Trans. abridged, Vol. IX, Part III, p. 206. Mikles. Medical Essays, Vol. II, p. 368. In Phil. Trans. Vol. IX, for 1674, p. 100, there is a "Relation of Women of 60 and 66 years of age giving suck to children."

|| Antoine François Jenin de Montègre, D.M. Gazette de Santé, 1810, of which Journal Dr. M. was Editor, from 1810 to 1818; he died in Hayti, 4th September, 1818; his excellent "Traité Analytique de toutes les Affections Hemorrhoidales," is analyzed in the Medico-Chirurgical Journal, Oct. 1818.

that, to convince the unbelieving, she would frequently spout it above a yard from her: a particular which, among others, the good man and woman of the house, and others of the neighbourhood likewise, assured me of. Now her left breast is run dry, and she has no great quantity in the right; but what there is, is as good milk as one may desire in a nurse. The poor woman seems perfectly honest and artless, and even inclines strongly to dotage: she very religiously throws the whole upon a miracle."

II. *Dr. Montègré's History.* Having collected a number of analogous facts, Dr. M. published them in the 'Gazette de Santé,' in connexion with the following, which came under his own observation. "La Femme Charles," a delicate female, had male twins in 1810, but from the great weakness of her constitution, she could hardly supply one of them with milk; and, in consequence of extreme poverty, was unable to procure the services of a nurse. Affected by this discouraging embarrassment, says Dr. M., her mother took one of the infants and put it to her own breast, which the babe instantly seized and began to suck. This ingenious grandmother was then in the sixty-fifth year of her age, and had been twenty-nine years a widow. At first her breasts yielded a fluid, in small quantity; but in a few days, her infantine grandchild drew from them an abundance of healthy milk. This tender nurseling continued for two and twenty months to be nourished at its grandmother's breasts; and throughout all that period was more vigorous than its brother who imbibed his sustenance from their common parent.

III. *Dr. K.'s History.* Judith Waterford lives in the village of Thringston, on the Forest, between Ashby-de-la-Zouch and Loughborough, Leicestershire. At this time (*December*, 1831), she is in her eighty-first year, and though infirm, her constitution has singularly retarded the advances of old-age.

Through life, this woman has been the active and laborious inmate of a peasant's cottage; her person is short and well-proportioned: at one time she weighed about 14 stone: her temperament is the neuro-sanguineous, distinctly marked. The peculiar circumstances of her history being extensively known in the district where she resides, she has been visited by many clerical, medical, and other curious inquirers.

J. W. had two husbands. Previously to her first marriage, her menstrual secretion appeared at regular periods: it continued nine days usually, and was very profuse, but preceded and accompanied with little pain or disturbance of the system. After this went, it returned only nine times, at distant and irregular intervals; and on each of these occasions it was abundant, without causing any perceptible diminution of her milk.

J. W. was first married in 1777; her first child was born in May 1778; and from that period till May, 1825, her lacteous secretion never in the least subsided. Besides giving her breast freely and frequently to the young ones of her neighbours, she suckled six children of her own by her first husband, and eight nurselings: she had two miscarriages. Her first confinement was followed by an excessive uterine discharge, which continued three weeks and induced considerable debility. No infant, however vigorous, was ever nearly able to use her supply of milk; and, in consequence, she often had her "breasts drawn" to the amount of two quarts in a day: her belief is, that, in general, she could have suckled four lively children, at the same time. Many attempts were made, even under medical direction, to suspend the secretion of her milk; but they all utterly failed. Her breasts often became tense and painful; and, for the removal of this state, she had them well rubbed with butter made from the cream of her own milk; this process was invariably advantageous. Sometimes about a pint of this cream was collected, and the butter it yielded was white, and soft as lard, with a sweet taste. Judy took much food, and seldom had recourse to medicine; it is her boast, indeed, that

"she never paid a shilling in her life for doctoring, for the sake of her health;" her bowels generally were torpid, often constipated.

J. W. was a widow nearly three years, and gave suck to her own and other children during the whole of that period. She had one still-born child to her second husband. Her breasts even now retain a size quite extraordinary, and the axillary glands are occasionally large. Soon after the disappearance of her milk, in 1825, her health became more variable, her energies gradually failed, and her voice, which still is strong, lost its natural strength. Her actual powers of mind seem to have experienced little change; she continues "heart-whole;" her appetite is considerably impaired; she wishes to eat often, but takes little food at one time; her sleep is disturbed and unrefreshing, and a cough gives her occasional annoyance; her pulse is full, soft, not quick, and resistant. For many months, she has been slightly troubled with those periodical disturbances by which menstruation is preceded.

Although Judy's milk ceased, for a time, in 1825, it repeatedly appeared in minute quantities during the five subsequent years; but, since the Autumn of 1830, the secretion of this fluid has been constant, and, if encouraged (she thinks), would be sufficient to nourish a child; from attempting this, however, she is deterred by the idea, that "it would soon be the end of her."

December 6th, 1831. This day, being in the middle of her 81st year, Judy readily filled a small spoon with her milk, by squeezing her left breast frequently with the hand. This milk was rich and sweet, and not different from that yielded by young and healthy mothers.

Here, then, are the remarkable circumstances of a woman who menstruated during lactation—who suckled children uninterruptedly through the full course of forty-seven years—and who, in her eighty-first year, has a moderate but regular secretion of milk.

---

### LXII.

### ANEURISM, FROM WOUND OF THE BRACHIAL ARTERY IN VENÆSECTION.*

"FOUR forms of aneurism may arise in the bend of the arm from a wound of the brachial artery (sometimes the radial) in venæsection, aneurismal varix, varicose aneurism, diffused and circumscribed false aneurism. Respecting the treatment of the two last, considerable difference of opinion exists among practical surgeons, some contend that both require operation, whilst others maintain their curability by compression.

The advocates for the operation have not as yet decided upon the precise situation where the vessel should be secured, nor upon the mode of securing it, some apply a ligature upon the vessel above the aneurism, and depend upon the absorbents for the removal of the tumour, whilst others open the aneurism and tie the artery above and below the wound.

The supporters of the mode by compression have not agreed either upon the place where the compressing force should be applied, nor upon its degree; some direct violent pressure to be made upon the vessel above the aneurism to obliterate its canal, whilst others are content to apply very slight pressure to the tumour itself.

In the midst of such conflicting opinions, we must appeal to facts, before we can arrive at any thing like a satisfactory conclusion."

CASE 1.—*Circumscribed Traumatic Aneurism—Cure by Compression.*

John Miley, æt. 27, applied at Stevens' Hospital on the 11th October, 1828, with circumscribed aneurism of the brachial artery in the bend of the arm. The tumour was firm, pulsatory, about the size and shape of a pigeon's egg, apparently intimately connected with the artery. It was diminished by pressure, and regained its size on its removal. The radial pulse was weaker on that side than on the other. The integuments presented a small cicatrix.

---

* Dublin Journal, No. II.

The patient had been bled *by a black-smith* thirteen days previously. The patient observed "the beating tumour" on the removal of the bandage several days afterwards. The case appeared a fair one for the employment of compression.

"The patient was immediately confined to bed, the limb placed upon a pillow, some cathartic mixture prescribed, and sixteen ounces of blood abstracted from the opposite arm.

The next day, a thin compress of wetted lint was laid upon the aneurism, and a roller bandage applied from the fingers to the bend of the arm, in the manner recommended by Genga, care being taken that the compression should not extend above the aneurism, the turns of the bandage over the aneurism were very loosely applied. The patient was desired to keep the compress constantly moistened with cold water, and to take a draught containing two drops of tincture of digitalis every sixth hour. Two days subsequently, the bandage was opened and re-applied, the draught was continued, and he had sixteen ozs. of blood taken from the opposite limb.

On the 7th day, the aneurism felt more solid, and did not diminish so sensibly when compressed. The bandage was re-applied, and a compress of sponge substituted for the lint. The digitalis continued, and other treatment as before.

On the 12th day, the aneurism appeared sensibly diminished, and much more solid; the pulsation in it was less distinct, whilst that at the wrist appeared more full. Bandage continued; the turns which passed over the compress being applied more firmly than before. Pulse 66. Patient is in perfect health, but complains much of the strict antiphlogistic regimen which he is compelled to observe. He was ordered to take a draught, containing thirty drops of tincture of digitalis, every second hour.

In the evening his pulse was down to 38. He did not complain of nausea or giddiness. The draughts were discontinued.

On the 14th day, the digitalis was administered in doses of twenty drops every sixth hour.

On the 20th day, the tumour was not larger than a marble, perfectly hard and without pulsation. The radial pulse beat firmly, and nearly as full as on the opposite limb. The bandage was re-applied, the compressing force being increased. Digitalis omitted. Antiphlogistic treatment still persevered in.

On the 30th day, there was no trace of the aneurism; the brachial artery could be felt pulsating strongly beneath the cicatrix in the integument, and the radial pulse appeared as full as that of the opposite limb.

On examining the patient eighteen months subsequent to his dismissal from the Hospital, no trace of the aneurism could be detected, the artery pulsated beneath the cicatrix strongly; the pulse at the wrist felt perfectly natural, and he stated, that he had not suffered the slightest inconvenience from the arm, in pursuing his very laborious occupation."

Case 2.—*Circumscribed Aneurism—Compression—Cure.*

Terence M'Evoy, æt. 25, was admitted into Dr. Stevens' Hospital March 2d, 1829, with circumscribed aneurism at the bend of the arm. The tumor was distinctly circumscribed, fully as large as a turkey's egg, pulsating, uniformly elastic, except at its anterior part, where it felt soft and pulsated most strongly. Pressure on the brachial artery stopped its pulsation but did not diminish its size. An artery of considerable magnitude ran over its anterior surface, but did not seem to be connected with it. The integuments were healthy; an old cicatrix was observable on the centre of the swelling. Nine months previously he had been bled, and, on removing the bandages three days afterwards, he noticed a small pulsating tumour. The treatment so nearly resembles that adopted in the preceding case, that we need not stop to particularize its items. On the 50th day there was no trace of the aneurism, the artery which had been wounded could be felt pulsating strongly beneath the cicatrix at the bend of the arm, and the vessel which ran across the tumour (the radial) appeared perfectly natural.

CASE 3.—*Circumscribed Aneurism—Compression—Aneurism rendered diffused—Operation—Cure.*

John Byrne, æt. 35, admitted Feb. 25th, 1830, with circumscribed aneurism of the brachial artery, produced by wound of the vessel in venesection five weeks previously. The tumour was oval, as large as an orange, adhering intimately to the brachial artery, solid at the base, soft and elastic in the centre. On compression of the brachial artery the tumour lost its pulsation, and became softer.

The same treatment was adopted as in the former cases, and, on the eighth day, the tumour appeared more solid. The pressure was re-applied with more firmness. He passed a restless night, and on the following morning complained of great pain in the tumour and arm. The aneurism was found to be no longer circumscribed, but to extend along the inner edge of the biceps muscle, towards the axilla, for several inches. The whole swelling, particularly the upper part, was soft and fluctuating; the pulsation throughout not very distinct; in short, it was now a case of diffused aneurism. In consultation, it was determined to treat it as a wounded artery. The tumour was fairly opened into, the coagula turned out, the artery tied above and below the wound in it. The ligatures were thrown off on the twelfth day, and, on the 30th, he left the hospital, quite well.

"It is manifest, from the foregoing cases, that the form of aneurism to which pressure is most applicable, and in which it is most likely to effect a cure, is that of circumscribed false aneurism, unattended by inflammation either of the sac or surrounding parts.

It is equally obvious, that the pressure should be applied to the tumour alone, and not to the artery above, and that the degree of pressure be slight until the diminished pulsation and solidity of the tumour indicate that coagula fill the sac, otherwise the aneurism will become diffused, as took place in the third case.

It is probable, if not certain, that the opinion hitherto entertained respecting the obliteration of the artery at the part wounded, is, in the majority of cases, erroneous and without foundation. The pulsation beneath the cicatrix remained strong and natural, after the absorption of the tumour in both cases, and in a case of circumscribed false aneurism in the bend of the arm from bleeding, which Mr. Wilmot cured by pressure several years ago, in Jervis-street Hospital, the artery continues to pulsate strongly beneath the cicatrix to this day.

The utility of combining Valsalva's mode of treating internal aneurisms with gentle pressure on the tumour itself, was well exemplified in these cases."

---

LXIII.

ABSCESS OF THE LIVER—PUNCTURE—DEATH.

JOHN EVANS, æt. 23, a sailor, admitted Jan. 5, under Dr. Elliotson, with a great but circumscribed tumefaction over the hepatic region, extending to the umbilical, with fluctuation at its lower part. The pulse was full and irregular. He had been for five years engaged in a voyage to and from the West Indies, and for the last five had been trading between London and Bourdeaux. He had been ill for 12 months, and attributed his complaint to exposure to cold and inclement weather. He was first attacked with bilious vomiting, and acute pain in the right hypochondriac region, the whole surface becoming of a saffron hue.

*Hirud.* xx. *regi. hepatis—cal.* gr. v. *o. n., ol. ric. o. m.*

"6th. Continues the same.

"A linseed meal poultice ordered to be applied to the abdomen, and six grains of calomel to be given every night: to be kept on milk diet.

11th. Complains of very acute pain over the region of the liver.

*Twelve leeches were ordered to be applied immediately to the part affected,*

*and one grain of opium to be taken; five grains of calomel to be continued twice a day.*

14th. Appears somewhat easier, but still complains of great pain over the abdomen, very much increased by pressure. The same medicines to be continued.

16th. Continues the same. *Ordered six grains of calomel, to be taken three times a day.*

20th. The patient's health is a little improved, and there is an evident fluctuation over the tumefied region of the liver. A puncture was made by Mr. Green, at the lower part of the umbilical region, from which four quarts of a thin and highly fetid purulent matter escaped. Since the operation the patient has been much easier, and can lie down without pain.

24th. Complains of great weakness, the pulse rather full, soft, and easily compressed.—*Ordered arrow-root and two pints of milk daily.*

There has been a discharge of hydatids from the puncture.

29th. Appears to be gradually sinking: still complaining of great pain on pressure over the whole abdomen. *Ordered three grains of opium to be taken immediately, and half a pint of wine, daily.* If the pain should increase, the wine to be diminished.

28th, Being very urgent in his request to leave the Hospital, his wife removed him.

He died on Monday, the day after his removal, at 2 p. m.

*Post Mortem Examination.*

Strong adhesions of the liver to the surrounding viscera; the liver considerably enlarged, especially the left lobe, which was full of small collections of fœtid pus, and the surface of a dark colour, the right lobe almost entirely degenerated into a large abscess filled with numerous hydatids, its proper substance forming a large and thin cyst. The gall bladder empty.

The other viscera were not examined.

Dr. Elliotson remarked that this case closely resembled one described in Dr. Baillie's Morbid Anatomy."

---

### LXIV.

### Dr. M'Cormac on the Oil called Palm Oil.

We extract the following short notice on this oil from our Glasgow contemporary for May of the present year.

"The palm oil of commerce, is obtained from the Cocos butyracea, which we are told is a native of Brazil. Now we find that the greater part if not the whole of the palm oil in use, comes from the coast of Africa, by way of Liverpool and London. Then the cocos butyracea is either a native of Africa, which I take to be the case, or otherwise, the officinal palm oil of the Edinburgh pharmacopœia is procured from the African palm. This I know to be the case, from having seen the plant and its oil upon the spot, up the river Sierra Leone.

It is stated in our dispensaries, that the palm oil tree furnishes a yellow succulent fruit, with a fibrous pulp, containing a hard cartilaginous kernel, which last, by grinding and maceration, furnishes the oil. I shall now state the real process by which it is prepared, from which it will be seen that an error must have crept into our accounts on the subject.

The palm tree growing on the coast of Africa, furnishes at the base or origin of its leaves, clusters of a yellow succulent fruit. Each of these bears some resemblance to a grape shot. The bunches are of different sizes, and the fruit composing them of different shapes, as might be expected from their reciprocal pressure, although naturally round, when not exposed to it. The pulp of this fruit is soft, and of a bright yellow colour—it is from this that the oil is obtained. Within it lies enclosed a hard and thick-shelled stone, of a dark colour, within which is contained a firm white kernel of a pleasant oily flavour. This kernel also affords an oil, which is not yellow, but white—and not fluid but concrete even in Africa. I need hardly say that the yellow palm oil is quite fluid while in Africa, and that it is not until it has been exposed to the cold of our temperate regions, that it becomes solid—whereas the oil of the kernel, as I have said, is always concrete or nearly so.

Both the white and the yellow oil are obtained by expression. The latter is procured in immense quantities in Africa, where it is partly consumed by the negroes along with their rice and pepper, or fried with their fish; and partly exported to Europe, where its principal use is in the manufacture of soap.

It continues to possess a pleasant fragrant odour for a long time after its extraction, and holds the same importance among the necessaries of an African, that olive oil does, among those of an Italian or Spaniard. It affords an amusing spectacle to a new comer to witness a number of merry negroes squatting on their hams round a calibash of rice. They seldom use a spoon, but knead the grain into huge balls, which they roll over in a mixture of pepper, salt and oil, and then pitch them with unerring aim and surprising velocity into their mouths, whence they almost seem to descend unbroken into the stomach. The white oil is only used as an ointment for the skin, which it keeps nice and soft, while it at the same time prevents too great an excretion of perspiratory matter. Not content with the hue that nature had given them, I have sometimes seen fond mothers mix this oil with something like lampblack and rub their children over from head to foot, giving them a singularly lustrous appearance especially in the sun.

The palm tree is one of the most stately in the African forest, towering above the rest, as the lofty pine does at home over its fellow trees. Parrots are said to be fond of the fruit. I have seen it given to them after they were newly caught. Indeed the strong arched beak of this bird, seems to render it peculiarly fit for tearing the fibres of fruit asunder. The preceding statement affords but a trifling addition to our knowledge, but as every thing helps to swell the great mass, I may be permitted to bring it forward."

The brothers Lander in their amusing and not uninstructive Journal of their adventures in Africa, whilst in quest of the course and termination of the Niger, make several remarks on the great consumption of palm oil by the natives. Wherever they were hospitably received by the dusky chief of an African town, and chiefs and towns seem plenty as blackberries in that region of the sun, palm wine and rice or other native dainties swimming in palm oil, were usual and acceptable presents. The God of the white man and the black has neither forgotten nor forsaken his dark children. The palm tree is a gift above all price to the African. It furnishes him with a pleasant drink, which he may ferment, a valuable oil, a fruit from the nut, and materials to construct his hut and his canoe; it gives him, in fact, food, drink, shelter, and conveyance. "The juice which is called wine, is obtained by making a hole in the trunk of a tree, and inserting a piece of the leaf into it so as to form a spout; the liquid flows through this and is received into a calabash placed beneath it, which probably holds two or three gallons, and will be thus filled in the course of a day. It shortly assumes a milky appearance, and is either used in this state, or preserved till it acquires a rather bitter flavour."* It is curious that man in all situations, and, so far as we know, in all ages, has continued to procure an intoxicating beverage. The Scythian and Sarmatian, in lands where immense plains were broken only by lakes and forests equally immense, where cultivation was never even attempted, and the nomade tribes lived like the modern gipsies in waggons, and wandered over their wide tracts from their youth to their age, still contrived to convert the milk of their mares into a stimulating drink. We are told by the Landers that in the interior of Africa palm wine is drunk to excess, and parties are formed to partake of the fascinating liquid. Nay, in one place an epicurean community exists, the elders repairing at morn to the shade of a majestic tree, and sitting till night with their calabashes by their side, draining draughts which they would not barter for the mead in the

* Lander.

halls of Valhalla, discoursing of by-gone times, and quietly awaiting death with only the palm between them and the sky where their Pagan heaven is, and the mighty juice of the noble tree beside them.

---

### LXV.

### Case of Secondary Hæmorrhage in which the External Iliac, Inguinal, and Femoral Arteries were tied. With Clinical Remarks. By M. S. Buchanan, M.D.

There are few subjects in surgery on which the accumulation of authentic facts is more likely to throw light, or, indeed, on which light is more required, than that of secondary hæmorrhage. Not that we are deficient in valuable and certain information respecting it, but we want to go farther than we have gone, to advance our outposts, to gain possession of points which shall give some clearness to what is now obscure, some certainty to what is now uncertain. Where is the surgeon who has not been surprised with the occurrence of secondary hæmorrhage when he did not look for it, who has not seen it prove fatal without apparent adequate cause why it should continue, who has not seen it cease as suddenly as it appeared, and prove but a bugbear when it promised to be a formidable foe! In point of fact we do not understand the laws which govern it, the circumstances necessary to its occurrence or its cessation—if those laws are certain we do not know them, and if they are uncertain we do not know the amount of uncertainty. It is on this account that we notice the following case, and that we usually make it a point to republish in this Journal such instances of secondary hæmorrhage as are related in other Journals, and seem to be deserving of attention.

*Case.* A boy, 12 years old, fell on the 15th January from a railway waggon, the wheels of which pushed his body onwards before it to the centre of the track. A dreadful contusion of the upper part of the thighs and lower part of the belly was the consequence, the skin and subjacent tissues were killed, they sloughed, and on the tenth day after the injury the sloughs had separated. On admission into the Glasgow Hospital on the 25th, the whole of the right and left inguinal and iliac regions, with the hypogastric from the root of the penis to within a few lines of the umbilicus, was one mass of ulceration of a healthy florid appearance, and covered with thick benign pus. At one spot in the left groin, where the ulcerative process had dipped deeper than elsewhere, the granulations were flabby, of dark grey appearance, and in a state approaching to gangrene. His health was much impaired—he was much emaciated—pulse 140, weak—thirst urgent—countenance anxious—troublesome dry cough—hectic. Besides all this, there were bed sores and extensive bruising on the sacrum and dorsum of the ilium. He was put on generous diet, the wound was dressed with carded cotton, and he continued daily to improve.

"Early in the morning of the 2d February, (being eighteen days from the accident,) hæmorrhage to an alarm-extent took place during sleep, and before he awoke, and compression was applied, he had lost upwards of ℥xxx. of blood. Syncope then took place and a coagulum formed, which stopped further effusion. The bleeding was remarked to have occurred exactly over the femoral artery, which was felt distinctly pulsating under where clot had formed, and in the soft friable and sloughy spot mentioned in first part of case.

Ten hours after the hæmorrhage, the report in the Journal bears, that he had again revived by the application of warmth to his extremities, and mulled wine at intervals, which he swallowed greedily. His moaning, however, had returned, and his cough had again become very troublesome.

You will find by a reference to the minute reports of the 3d, 4th, and 5th, that his strength continued rapidly to improve, his restlessness, moaning, and short cough gradually disappeared, and his appetite became so keen that he entreated to be allowed full diet, which was in part complied with.

About 11 o'clock in the evening of the 5th, arterial hæmorrhage again appeared from seat of femoral artery; a consultation was instantly called, and compression with the finger had recourse to. On my arrival at the Hospital, I made a minute examination of the wound, and observed the arterial stream to issue from a small aneurismal pouch about the size of a field bean, in the centre of the soft flabby ulcerated surface of the left groin, and which with my finger I freely opened, but to my astonishment hæmorrhage again ceased completely. By this time the members of consultation had arrived, but, in consequence of the extreme exhaustion produced by the hæmorrhage, they were of opinion that compression with the finger over the site of the above-mentioned pouch, should be again had recourse to, after which, if the boy revived, and hæmorrhage recurred, from what was by the minority thought the femoral artery, then the external iliac should be secured by ligature.

The quantity of blood by this hæmorrhage having amounted to about ℔i., the extremities being cold, and the face of the little sufferer of a deadly pallid hue, with a fluttering and compressible pulse, I ordered him wine, *ad libitum*, and to be strictly watched, requesting the gentlemen of the consultion again to give me their assistance at the visit hour, 1 o'clock, P.M. I ought to have stated, that the majority in consultation thought that it could not be the main trunk of the femoral which was ulcerated, but the epigastric, and if so, that tying the iliac would be of no use, from the free inosculation of the former with the external mammary, and the great risk besides of mortification of the limb, from the above operation, in such a subject. To the first part of this position, I felt inclined to subscribe on a former occasion, and for the reasons then urged; but on the present, my mind was made up as to the source of the hæmorrhage, and the consequent practice. I felt somewhat at a loss, however, to account for this bell-shaped pouch over the main trunk of the femoral, the ease with which the stream was arrested, and the length of time which had intervened since the previous flow. The only way in which I could explain this peculiarity in the case was, that Nature in her effort to save life had, when the first hæmorrhage was arrested, spontaneously formed, between the coats of the vessel and the sheath, a layer of lymph,—that this becoming organized, constituted the bag alluded to, which being distended and ulcerating had burst, and after the application of the finger, that Nature again was in the act of renewing her former manœuvre, to the astonishment of all her spectators.

At 1 P.M., of the 6th, matters had much improved, and you will find by the very accurate, though lengthy report in the Journal of this date, after stating all the particulars of the case, that 'no further hæmorrhage had occurred.' The majority of the consultation were therefore of opinion, that nature should get, once more, a trial of her powers at plugging, prior to the dernier resort—the ligature of the artery. But I must say, that at this consultation there was a considerable difference of opinion, and I state this here, that all of you may know the facts of the case, and the arguments which were used, *pro* and *con*. as in my opinion, a knowledge of this consultation is of essential importance to the right understanding of all that afterwards occurred. In page 237 of Vol. III. of this Journal, in relating my case of ligature of the subclavian artery, you will find a *facsimile* of the case now under consideration; and the arguments there brought forward by the minority, were exactly of a similar kind to those advanced in the present case; namely, that hæmorrhage would undoubtedly again occur, and put it out of our power to proceed to an operation; and that the patient would never again have an equally good chance of surviving, nor an operation of being successful, as at the present moment."

In the evening of the 6th he was much revived, although he had taken nothing but wine. This was continued, and 15 drops of tincture of digitalis ordered to be given every fifth hour. Lunar caustic was applied to the flabby granulations round the hæmorrhagic spot, and cotton applied to the ulcerated surface. The patient improved, and, with the exception of another hæmorrhagic drain on the 24th, nothing untoward occurred till the 26th; the patient in fact appeared convalescent.

"I must here crave your attention to a few passing remarks on the history of this case, since the 14th, the date of last bleeding. I mentioned that at the consultation of the 5th, a kind of qualified permission was by the majority given, to throw a ligature round the external iliac artery, if hæmorrhage again occurred from the same spot. The qualification was, if the bleeding was distinctly observed to proceed from the trunk of the femoral artery. I need not inform any of you, how much opinions on this point changed, from the 5th to the 14th, or in other words, how eight days of calm and convalescence strengthened the impression, that such a formidable operation was quite unnecessary, and also the opinion that a small arterial branch only had given way, which time and perhaps compression would cicatrize. For this reason I did not proceed to the operation on the 14th, though much inclined, but rather preferred the sanction of another consultation to abiding by my own conviction, and notwithstanding the result of the consultation, as reported on the 15th, I still had the satisfaction of having done my duty. It may be said, that timidity rather than caution prompted me on this occasion, and I am free to confess that I had fearful forebodings of the result, for the reasons which shall hereafter be detailed; but a paramount feeling of duty to my patient, absorbed at this stage of the case, every other consideration. After the consultation of the 15th, however, I as it were resigned the case into other hands, and from this period ceased to look upon it as of the same interest, for I felt that no sooner had I made up my lee-way by the exhibition of tonics and stimulants, than all must again be lost by another and a more fatal hæmorrhage. True it was, I could not positively affirm that it was the femoral artery, in consequence of the soft, friable and sloughy state of the wound—true it was, I could not very satisfactorily, or according to any of the known rules of surgical pathology, explain the meaning of such long intervals of hæmorrhagic repose, or the process by which, so frequently, nature in this remarkable case, formed for herself a plug, in so large an arterial conduit; still, I was not singular in this opinion, founded as it was on the observation of nature's resources in aneurismal cases, and as the result will show you, I had the misfortune to be right—but you observe, with what difficulties I had to grapple, and against what odds I had at every step to contend. The application of lunar caustic, and the finger to the soft flabby granulations, over the hæmorrhagic spot, were resorted to, merely to assist in forming a coating of lymph, and prolonging the interval of repose, and from the success which had previously attended these efforts, I meant, (but for the event which I am by and by to detail,) to have continued them for some time longer."

On the 26th these favourable appearances were at once destroyed by another bleeding. At 3 a. m. arterial hæmorrhage again occurred from the sloughy spot already mentioned, but was immediately commanded by the finger, and not above ℥ii. of blood were lost. A consultation was called at 6, a. m. when, compression having been removed that the wound might be examined, the arterial gush instantly took place, apparently from the femoral artery, exactly over the spot whence all previous hæmorrhage had issued, but it was at once arrested by the finger. The vessel and sheath seemed to be in a soft friable state. The pulse was firm, and he was not depressed.

"The consultation, which consisted of Drs. M'Farlane, Weir, and Auchincloss, being unanimously of opinion that the external iliac artery should, without further delay, be secured by ligature, the operation was performed in the following manner:—I made an incision from outer opening of inguinal canal, to about half an inch from anterior superior spine of ilium, about an inch above Poupart's ligament. The first incision was straight and carried through skin and cellular substance. The superficial fascia covering external oblique muscle, was then divided upon the director through whole extent of

P 2

wound, and the fibres of external and internal oblique were thereafter incised in a similar manner, exposing fascia transversalis and peritoneum. I now introduced my finger into wound, and with my nail detached with ease the peritoneum from sheath of external iliac artery, to the extent of about three-fourths of an inch, and having now passed the common aneurismal needle under it, from within outwards, armed with a single silk ligature, I secured the vessel by a double knot, carrying the ends on the points of my fore-fingers to the bottom of the wound, which was nearly an inch in depth. Pulsation and hæmorrhage were instantly arrested, and one end of ligature being allowed to remain, lips of wound were approximated by a stitch in centre, and adhesive plaster. He bore the operation well, and though it lasted about twenty minutes, not two ounces of blood were lost."

Into Dr. Buchanan's reasons for modifying the operation in the manner in which the well-informed reader will perceive that he did, we need not enter. Until March 1, when pulsation was first perceptible both in the femoral and popliteal arteries, nothing of any moment occurred. The patient was moderately supported. At 7 o'clock of the morning of the 2d, hæmorrhage again occurred from the same spot as before. Only one ounce of blood was lost, the little patient making immediate pressure himself. Till the visit hour at half-past one o'clock compression was steadily maintained.

"When visited about half-past one o'clock, it was evident that collateral circulation had become completely re-established, directly through femoral artery upwards to inguinal, from the gush of arterial blood which had taken place at two different periods since 7 o'clock, on attempting to remove the finger from the hæmorrhagic spot; and also from suppression of bleeding and pulsation, by compression of femoral artery, on the distal side of place whence blood issued. On this account, with the concurrence of my colleague, I proceeded to throw a ligature around the femoral artery at the place, where compression arrested the reflux channel. For this purpose, the patient was placed in the same position as in the operation of the 26th, the finger of my assistant during the performance of the operation being kept firmly applied over bleeding spot. I then made an incision about 1½ inches in length, in a slanting direction, from border of fascia lata, towards inner side of sartorious muscle through skin and cellular substance. Femoral fascia was now incised in centre of wound, a director being introduced, first upwards, then downwards, and the membrane divided upon it with a scalpel. Cellular substance between this last and fascia propria was now cautiously cut, the artery separated from the vein, isolated to about one-fourth of an inch, an aneurismal needle armed with a single silk ligature passed under it and tied, one end being cut away close to vessel, and lips of wound approximated by straps of adhesive plaster. The operation occupied about ten minutes, and was almost bloodless. Trusting to the report of the young gentleman who made compression with the finger over the bleeding spot, I tightened the ligature, and secured the femoral at the spot above-mentioned, but most unfortunately, on attempting to remove the finger of the assistant, the gush of arterial blood was as copious and furious as before. The application of the actual cautery, button forceps, and pelvis tourniquet were now thought of, but as soon abandoned. Compression having been applied between the ligature, around femoral artery, and bleeding spot, it was found, that both pulsation and hæmorrhage were by this means instantly arrested, though there were not seemingly above two inches between the above situations, and more than the half of this intervening and interesting part of the arterial conduit, was covered with soft blue flabby ulceration. For the above reasons I determined, with the assistance and concurrence of my colleague, to take up the inguinal artery as near the spot where hæmorrhage had appeared as possible, without running the risk of intermeddling with the soft and diseased part of the tube. For this purpose I made an incision from superior termination of former one, upwards through cicatrix of ulceration, and by this means exposed falx of fascia lata, I now introduced my director under this last and Poupart's ligament, and with my bistoury incised both upwards to the necessary extent; my finger now easily traced inguinal artery to within about half an inch of bleeding spot, and finding it, as I thought, in a sound state, proceeded to isolate it from the vein and anterior crural nerve, and with ease passed the aneurismal needle, armed as before with a single silk thread, on tightening which, pulsation and hæmorrhage were instantly arrested. In this last operation, which did not occupy more than two minutes, as little blood was lost as in the former one, and the after dressing was conducted in the same manner.

It was remarked by the little patient that not only during last night, but on every previous occasion immediately preceding the hæmorrhages, he had a *presentiment* of what would occur, both from a peculiar restlessness, and also from being annoyed with unpleasant dreams."

During the night, the patient was restless and incoherent. On the 3rd, no pulsation could yet be detected in the femoral or popliteal artery—the left limb was colder than natural—pulse 144 and weak. He took wine and water. On the 4th the pulse was 144, jarring and hæmorrhagic. During the preceding night, he had had a rigor of about a quarter of an hour's duration, a certain harbinger of death. In the night of the 4th he had another rigor, and in the afternoon of the 5th another. On the 6th, pulsation had returned to the popliteal artery. His strength now gradually gave way; there was profuse discharge from an abscess on the dorsum of the ilium, and a sore upon the sacrum, and yet, under quina and support, he again plucked up. The femoral and abdominal wounds produced by the operations rapidly cicatrized. On the 12th (10th day), the ligatures were detached from the inguinal and femoral arteries. On the 13th he began to be affected with dry tickling cough and uneasiness of chest, the pulse was 140, abdomen somewhat tense. In the following evening he vomited his wine and arrow-root, sank rapidly, and died in the afternoon of the 15th.

"*Post-mortem Appearances.*—Contents of cranium, thorax and abdomen, having been examined, were found perfectly natural. A wax injection was thrown into left common iliac artery downwards, and into popliteal artery upwards. Abdominal viscera having been entirely removed, peritoneum lining the abdominal parietes was found to be in every part entire, and on sliting it open, from bifurcation of common iliac to left groin, over course of external iliac artery, this vessel was found injected only about two inches in its progress, the remainder of the artery downwards having degenerated into a soft substance, still attached to psoas muscle. The superior portion so far as it allowed the injection to pass, was very much reduced in caliber, not being larger than a crow-quill. The internal iliac was about the size of the little finger, and its gluteal branch was traced through the ischiatic notch filled with injection, and anastomosing freely with the external circumflex, which in this case went on from the profunda; external iliac, from spot where ligature had been applied downwards to seat of hæmorrhage, was completely obliterated, and epigastric artery, which went off about an inch below the tied portion, was so small as to be with difficulty traced. Injection had passed freely upwards, from popliteal artery to within half an inch below the place where lower ligature was applied round femoral; here adhesion of the coats was complete. On tracing the remains of femoral artery, from situation of third ligature, upwards to seat of hæmorrhage, the tube was found in the same state as inferior portion of external iliac, mentioned above. The pouch, which was remarked at the second hæmorrhage, was now well seen, and found to consist of the sheath of main trunk of inguinal artery, and when its soft friable walls were removed, an opening about the size of the point of the little finger was observed at the outer side of main stem, the whole circumference of which, and also the caliber of the vessel, for the space of more than half an inch, partook of the same morbid appearance as the above portion of the arterial sheath. Profunda could only be traced from below to within half an inch of spot where third ligature was applied; here it was confounded in the general disorganization of parts. On dorsum of left ilium, extending laterally from anterior superior spinous process to sacrum, and inferiorly to tuberosity of ischium, was an extensive abscess, excavated into irregular hollows, occupying the whole external surface of left os innominatum. To this abscess there were many openings, the largest of which was situated on the dorsum of left ilium, immediately behind anterior spinous process. On right haunch were two irregular sloughy spots, and on anterior surface of left there were similar marks of disease."

Thus was this long but interesting case concluded. Without entering into any speculations, or indulging in any criticisms, we cannot but regret that the operation was not earlier performed. We do not clearly understand, from the description of the state of the parts after death, whether the profunda joined the superficial femoral between the second and third ligatures, or above the latter. If it did not unite itself to the femoral between the ligatures, or if no other branch of any size arose in that spot, we are left in the dark as to the cause of the hæmorrhage after the application of the second ligature, and its cessation after that of the third. If it did join the femoral in this situation, another reflection naturally presents itself. When a surgeon is aware of a large artery, like the profunda, taking its rise from the inguinal, he should surely refrain from placing a ligature on the femoral below that origin, in order to arrest hæmorrhage from the artery above. The blood getting back through the profunda into the inguinal, is not arrested in its retrograde course by such an operation. It is no excuse to assert, that ulceration in the groin renders it dangerous to tie the vessel where its tunics are probably unsound. Under such circumstances, it is absolutely necessary to tie the vessel as near the source of the hæmorrhage, as a healthy condition of its coats can possibly permit.

---

### LXVI.

On the Use of the Nitrate of Iron in Diarrhœa. By Mr. W. Kerr, of Paisley. Ed. Journal, January, and Glasgow Journal, April, 1832.

The medicine here recommended is a solution of iron wire, or small pieces of iron, in nitric acid, so as to form a persesquinitrate. This, in moderate doses, has been found very efficacious in diarrhœa, and some other affections of the mucous membrane of the alimentary canal. In our Glasgow contemporary for April, Mr. K. has published some more cases, illustrating the efficacy of the medicine in the premonitory diarrhœa of cholera.

---

### LXVII.

A Practical Treatise on Uterine Hæmorrhage, in connexion with Pregnancy and Parturition. By John T. Ingleby, Member of the Royal College of Surgeons in London, one of the Surgeons to the General Dispensary, Surgeon to the Magdalen Asylum, and Lecturer on Midwifery at the School of Medicine in Birmingham. One Plate, 8vo. pp. 276. London, 1832.

That the art of midwifery, on the scientific and successful practice of which the lives of the most interesting portion of our species often depend, should have received no legal protection, and been controlled by no authorized regulations in Great Britain, a country so pre-eminently distinguished for humanity, appears almost incredible. The commercial spirit which pervades every part of the empire, and appreciates every pursuit in proportion to the pecuniary advantages it may afford, seems to have penetrated into the sanctuaries of physical science. Hence our great chartered bodies, having relinquished this branch of the profession, and being no longer competent to ascertain the qualifications of candidates, the most ignorant adventurers are permitted to abuse the public confidence, and endanger the lives of His Majesty's subjects, without incurring any fine or penalty. To this neglect and degradation of the obstetrical art, we may trace the constant occurrence of disgraceful and fatal errors in practice and the paucity of publications, in comparison with those in other departments of therapeutic science.

"Questions, it is true (observes the author), are sometimes proposed to the candidate for diploma at Apothecaries' Hall, although not forming a necessary part of the examination, whilst examiners at the College of Surgeons take no cognizance whatever of the obstetrical qualifications of the student, a circumstance which sufficiently accounts for the indifference, not to say contempt, with which many regard this study."—*Preface, p.* viii.

He might have added, that licences to practise midwifery were formerly sold at the Royal College of Physicians in London; but, after it was discovered that the Fellows of that learned body were, by their own bye-laws, restricted from acquiring any practical information on the subject, their licenses became obsolete and despised.

To remedy one of the vicious consequences arising from those minute and arbitrary subdivisions of the profession, which were the offspring of academical pride and popular prejudice, an attempt was made to form an obstetrical society in this metropolis; and it is much to be regretted that such a needful institution was not supported and extended by legal enactment, as no branch of the healing art is so fit for, or so likely to be promoted by, separate cultivation. We would, however, on no account recommend the study of one branch to the exclusion of the whole; for we believe that any one, understanding every portion of a science, must be best adapted for exercising or improving any distinct part. Whoever, therefore, may expect to refine and improve his practice by contracting his education, would be like an agriculturist, who, instead of cultivating all his land with a view of producing cheap and wholesome food, of which all may partake, confines his attention to his greenhouse, that he may excel his neighbours in the propagation of expensive and useless flowers. In this respect, the mere physician or surgeon is often found to labour under a decided disadvantage, where his limited education is brought into practical competition with the omniscience of the general practitioner, "omnia scire; non omnia exsequi."—*Tacitus.*

The neglect with which the midwifery department has been treated by the Colleges of Physicians and Surgeons in Great Britain, has not escaped the notice of the profession at large; and we hope the time is not far distant, when the obstetrical professors will combine their efforts for the purpose of establishing a national institution, which may improve and regulate the cultivation and exercise of an art so useful to the public.

In the preliminary observations, with which the able work under review commences, the author reminds the young practitioner of the absolute necessity of obtaining an intimate knowledge of the various duties he may be suddenly called upon to discharge; as time and circumstances, especially in remote parts of the country, will often not admit a consultation.

"In the graver hæmorrhages, whether antecedent or subsequent to delivery, alacrity and promptitude are indispensable; hesitation is often fatal in its consequences, life is fluttering away, and, when time does not allow of a second opinion, the practitioner is at once thrown upon his own resources."

"If the student is not impressed with the value of human life, his own self-interest, so closely interwoven with his success in the obstetric art, paramountly demands an intimate knowledge of these very duties."

The infrequency of death, during or connected with the process of parturition, in the country in comparison with the metropolis, is accounted for by the author from the superior salubrity of the atmosphere and moral and physical condition of the patient, which, in preventing disease, and promoting recovery, operate with equal advantage. To these may be added the intimate knowledge of midwifery which the well-educated country surgeons are found so pre-eminently to possess. The most common causes of death appear to Mr. Ingleby to be hæmorrhage, and peritoneal (inflammation, improperly called) fever; and Mr. Robertson, one of the surgeons to the Manchester Lying-in Charity, asserts that more fatality arises from hæmorrhage than from all other causes. The brute creation, in the natural state, from the peculiar economy of the placenta, are exempt from those fatal hæmorrhages; but, when domesticated, some of them have been known to die from this cause. We have also found females of the canine race subject to a peculiar cause of death, namely, mortification of the uterus, before expulsion of its contents has been effected; which fact we have ascertained by repeated post-mortem examinations. In these cases, as soon as mortification commences, the uterine contraction immediately subsides. The remote cause of this extraordinary circumstance has been found by us to consist in a remarkable disproportion between the size of the mother and that of the young; the latter appearing much too large for expulsion by the natural efforts; and a corresponding disproportion has, on inquiry, been found to exist between the male and female parents. The decease of these animals, immediately after parturition has been completed, may probably be owing to some analogous cause.

The premature retirement of the accoucheur after delivery next occupies the author's notice, who makes some very proper remarks on the subject, accompanied by a detail of four cases of sudden death from flooding after delivery. We make it an invariable rule never to leave our patient, until we are satisfied that the uterus has undergone such a degree of contraction as may ensure her safety; and the vascular and nervous systems have recovered from the collapse, which often follows the most natural labour. When considerable hæmorrhage has taken place, the greatest care should be taken to prevent any sudden change of position; as women have been known to die in a moment, while in the act of rising up in bed for the purpose of taking food. In these cases, we are not to be guided by the amount of blood, but by the alarming effects produced from its sudden extravasation; after the constitution has been exhausted by previous pain and hæmorrhage. The best practice, in these cases, is to apply external pressure, which, the author justly observes, should be continued several hours, lest blood should insidiously accumulate within the uterus.

As a means of obviating hæmorrhage, and supporting the pendulous abdomen in the latter months of gestation, the author extols the use of bandages, particularly Dr. Gaitskell's, which he has modified so, that it is equally applicable before or after parturition, as will appear from the following note:

"I allude to the bandage commonly used; but I have modified Gaitskell's, so as to answer for either state, by inserting on each side, for the hips, two pieces of cloth, commonly termed gores, four inches in depth and three in width at the lower edge, and placed one inch and a quarter apart. Two plates adapt it for fitting the back. The bandage has two rows of loops, with corresponding tapes, and averages from 30 to 36 inches in width, and from 15 to 18 in depth at the centre. As buttons are placed at different distances round the lower edge of the bandage, understraps may be used if needful. This answers every purpose, and can be made sufficiently reasonable to be within the reach of the most indigent."

The application of a bandage having been neglected before serious hæmorrhage occurs, the simple plan of applying three napkins, described by Dr. Blundell in the Lancet, No. 265, page 801, may be adopted; or the following, recommended by the author.

"Rather than disturb a woman at such a moment, it will be better to pass a large towel or broad piece of linen *external* to the clothes, and draw it as tight as it can be borne. The discharges and wet linen, including the napkin and folded sheets, must be removed from about the person, as soon as possible, and dry linen substituted. After reposing an hour, or longer if needful, a suitable bandage may be applied. Care must be taken in conveying it round the body, not to disturb the position of the patient. The under garment, which during the last stage of labour ought to be taken away from the discharges, may now be adjusted; and a petticoat, which opens in front, being drawn underneath her, the broad top-band should be pinned sufficiently tight to afford an agreeable support. If the bed has been properly prepared, the patient may now be placed in it, or if she is already in bed, laid on the opposite side, slowly and without raising her."

"After four or six hours have elapsed it may be necessary to slacken the bandage, agreeably with the feelings of the patient, whose comfort will also be promoted by excluding the atmospheric air from the external parts."

Some useful directions follow, respecting temperature, repose and diet after the completion of the parturient process.

*After-pains* seldom occur as a consequence of the first labour; and, if our experience with the secale cornutum does not deceive us, we believe that they may be greatly modified by its exhibition before the detachment of the placenta, especially in such individuals, as have been formerly found to labour under a flaccid state of the parietes and feeble contraction of the uterus. These pains are of two kinds, active and passive: the former consisting of the natural, contractile efforts of the womb, and the latter of a forcible dilatation, occasioned by an influx of blood, often suddenly distending it to an enormous size. In all cases, when the uterus is distended with blood, which either from large, firm coagula or any other obstruction cannot escape, severe pain and tenderness are experienced; and these symptoms in extreme cases continue, until such a degree of decomposition of the extravasated blood takes place, as permits the entire evacuation of the uterus. This process frequently occupies several days, and simulates uterine enlargement, produced by sub-acute inflammation. This kind of after-pains is peculiar to persons advanced in life, or who have had several labours, and is probably that described in the following passage:

"But when severe and long continued, these paroxysms sometimes occasion febrile dirturbance, the uterus becoming large, and highly sensible to the touch; and when attended with *a defective flow of the lochia*, they may be considered as denoting a congested or inflamed state of the large uterine veins."

The active species of after-pain, particularly when proceeding from a partial contraction, will often require the aid of opium; the other is most effectually relieved by ergot of rye, assisted by an open state of the bowels.

The sub-acute inflammation, the seat of which is in the fibrous structure of the uterus, makes its appearance from the fifth to the tenth day; and, when it has attained its height, the organ acquires a magnitude fully equal to that, which it possessed at any period before the commencement of labour. It is accompanied with extreme tenderness, fever, thirst and a pulse at 102. The blood, when coagulated, has a thick buff on its surface, but is not cupped. The treatment consists in the abstraction of blood in small quantities, followed by the application of leeches and gentle mercurialism.

The customary period for decumbiture after parturition is five days. We agree, however, with the author that, after a severe labour or hæmorrhage, a much longer time should be enforced; and, where prolapsus is apprehended, it should be extended to several weeks.

"This plan has been attended with complete success, even in cases in which the uterus in the early months of gestation had fallen completely through the vulva. Very recently I was called to reduce, at the fourth month of gestation, a gravid uterus, which lay completely without the os externum. By means of the largest-sized globular pessary and the horizontal position, it was prevented, after its reduction, from again descending, and thus the ascent in the abdomen would be accomplished almost as early as in common cases."

By attention to position alone after confinement we have uniformly found prolapsus, occasioning the greatest distress before pregnancy, which of itself in the latter stages is a temporary cure, have been entirely obviated.

In the chapter on *menstruation occurring during pregnancy* the author states that the menstrual secretion rarely appears in the last six or eight weeks of utero-gestation in these cases, and believes that it proceeds from the uterus in opposition to the opinions of Denman and Hamilton. This however is not an original sentiment; for Cruikshank long ago supposed the secretion took place from the exhalant arteries of the uterus, which experience a periodical enlargement for this purpose.*

To evince the fact of the absence and termination of the decidua at the orifices of the fallopian tubes, the author relates the appearances observed in a fatal case of extra-uterine pregnancy, occurring in the practice of Mr. Bellamy.

In females of a plethoric habit *a hemorrhagic tendency is sometimes acquired by pregnancy,* which, neglected or allowed to proceed, usually occasions such a detachment of the placenta, as is inconsistent with the life of the embryo or fœtus. Hence arise most of the abortions in such constitutions. The most effectual treatment consists in adequate depletion, temperance and abstinence from violent exercise. There is a passive kind of hæmorrhage, the author observes, which arises from constitutional weakness; and to this class may be referred that which proceeds from a mechanical obstruction to the circulation.

*Hydrops Amnii,* a preternatural secretion from the amnios, next engages the author's attention; and in a note is recorded the following extraordinary case.

"A lady, subject to uterine hæmorrhage, was afflicted with dropsy of the ovum in her second gestation. At the fifth month her size was prodigiously large. The fluctuation was as distinct as in a case of ascites, and the legs threatened to burst. Indeed, had she not experienced aggravated symptoms of pregnancy, no one would have suspected it. At the sixth month the patient, who had then abandoned the idea of pregnancy, was delivered of twins, and a deluge of water reduced her to a state of imminent peril. In the third pregnancy the same symptoms recurred, but earlier than in the preceding. At the fifth month, she was as unwieldy as though she had reached the ninth. Small bleedings, abstinence from liquids, mild purgatives, diuretics, with the horizontal position, carried her on to the sixth—at this period the membranes unexpectedly gave way during the night, and twelve napkins were saturated with the fluid prior to my seeing her early in the morning. The singular part of the case is, that every second or third day the waters escaped, affording relief in proportion to the quantity discharged, being from a pint to a quart each time, so that fresh secretion must have been going on. I can vouch from frequent examinations that the fluid proceeded from the uterus. Either from the thinness of the uterus, or the small quantity of liquor amnii contained within the membrane after these discharges, almost every part of the child could be felt through the parietes of the abdomen; the knees could be distinguished from the elbow, and the head could be brought from the inguinal region to the hypochondrium, and *vice versâ;* just as the sutures of the fœtal head have been distinguished through the abdomen without lesion of the uterine parietes. Indeed, if the fluid had not been unequivocally passed through the os uteri, I should have regarded the case as an extra-uterine gestation. I scarcely need say that the fluid was not urine. Urine I am aware is not unfrequently mistaken for the liquor amnii in those discharges, which on muscular exertion are passed in the latter weeks of gestation. On one occasion the bladder was quite empty; on another, when the discharge was flowing away, I introduced a catheter, and drew off the urine. As the

* Cruikshank's Anatomy of the Absorbents.

ninth month advanced, the head rested permanently on the pubes, and could no longer be moved about freely. She attained the full period, and was delivered of a child unusually large. At the second pain the secundines were spontaneously expelled, my hand receiving the mass, when at the outlet, lest its weight should tear the membranes. I then carefully examined the whole, and in addition to the aperture in the centre of the membranes, made by the passage of the head, there was a circular one very distinct just at the edge of the placenta. From this aperture the fluid had doubtless from time to time escaped, the patient prior to each evacuation being sensible, by a kind of passive contraction of the uterus, that it was about to come away. The situation of the rent accounts also for my not being able to draw off the liquor amnii with a catheter, though the attempt was frequently tried with a view of accelerating labour. The longest period I had known between the rupture of the membranes and the accession of labour was eight days. A respectable surgeon, formerly of this town, knew a case, in which a month intervened."*

[*To be continued.*]

---

### LXVIII.

### Analysis of the Blood and Urine of Diabetic Patients.†

We need scarcely inform our readers that the labours of Dr. Prout have thrown considerable light on the various alterations of the urine coincident and connected with alterations of the system in general, or the urinary organs in particular. It has been stated, and apparently proved, by that able chemical physician, that in diabetes the highly-animalized principle urea is not to be detected in the urine, its place being supplied by sugar, a principle eminently vegetable. Prior to the appearance of sugar, in other words, before the diabetic condition is established, Dr. Prout asserts that albumen is found in the urine, and thus a regular series of changes is observed in that secretion, from its state of health to diabetes, and from diabetes back to its state of health. But it seems that chemistry has its fallacies as well as physic, indeed animal chemistry will, we fear, be for ever an uncertain branch of an uncertain science. For this gloomy notion we might offer several powerful reasons; but two will at present be sufficient—the varying character of the animal fluids, and their necessary alterations under the chemical re-agents to which they are exposed. Sure it is, that no two chemists will agree in their finer analyses of the animal solids or fluids. We do not intend to throw cold water on chemical investigations in connexion with physic; we think that they have already effected much good, and that they will yet effect much more. But we have seen enough to render us distrustful of the conclusions of this or that particular chemist, more especially if he deviates far from the ordinary track.

Mr. Kane, professor of chemistry to Apothecaries' Hall, of Dublin, has published a paper in the Dublin Journal, contradictory of the belief in an absence of urea from diabetic urine. He asserts that it is not even diminished in quantity in that disease. We shall not relate the manner in which he detected the error of Prout and others, but merely shew the method which he adopts to procure the urea. Before doing this, we may mention that he discovered that, if nitric acid (the re-agent by which the presence of urea is determined) be added to diabetic urine under ordinary circumstances, a considerable increase of temperature takes place, and urea is not obtained, a circumstance probably owing to some chemical combination between it and the acid. If the urine, on the addition of

---

* We regret that the late period at which we received Mr. Ingleby's important work prevents us doing much more than commence the analytical review of it. In our next number we shall give a full acccunt of the remainder of the publication.—*Ed.*

† Dublin Journal, No. I.

the nitric acid, be plunged into a freezing mixture, urea is procured in the quantity shewn by the following tables.

"A given quantity of the urine was desiccated cautiously as long as it lost weight, retaining the temperature constantly below 212°, the residue gave the relative proportions of water and of solid matter in the urine. To determine the quantity of sugar, an excess of solution of acetate of lead was added to the urine, and the precipitate separated by the filter. The lead in the filtered liquor was then thrown down by sulphuretted hydrogen, and the sulphuret of lead having been separated by the filter, the liquor was evaporated on a water bath to dryness, and the residue weighed; in some instances the confused crystalline mass was dissolved in spirit, and crystallized, but this part of the process was not found necessary.

The quantity of urea was determined by the process by means of which it had been first obtained in quantity; the mixture of acid and urine was immersed in a freezing bath, and the nitrate of urea deposited, separated, dried, and weighed, and the quantity of urea calculated from its known composition. The nitrate thus obtained, is slightly tinged, but being weighed in this state, it gave a closer approximation to the truth than if it had been purified and decolorized; in which case, a portion of it would infallibly have been lost. The quantities of urea given, are those actually found, and I consider that they are less than actually existed in the urine, as I only succeeded in diminishing very much the action of the acid on that principle, without, however, by any means putting a total stop to its exertion.

I think it probable, that the uric acid not having been found in diabetic urine, depends merely upon the great state of dilution of that fluid, and on the small quantity in which that animal acid exists even in healthy urine. Some trials which I made in order to isolate it, were not successful; want of time prevented me from determining the relative proportions of the different salts, but they certainly exist in the natural proportions, as their total quantity is diminished merely in consequence of the dilution of the urine.

The following are the results of the analyses of diabetic urine, performed on those principles.

A man in the Meath Hospital, April 20th, 1831.

Urine, Sp. Gr., 1032.

| | |
|---|---|
| Water | 929 |
| Sugar | 47 |
| Urea | 9 |
| Salts, Uric Acid, &c. | 13 |
| | 1000 |

Mackay, a patient in Sir P. Dunn's Hospital, clinique of Dr. Leahy, who kindly allowed me to examine the blood and urine:

Urine, Sp. Gr., 1030.25.

| | |
|---|---|
| Water | 942.75 |
| Sugar | 31.5 |
| Urea | 9.5 |
| Salts, Uric Acid, &c. | 16.25 |
| | 1000.00 |

A man in the Meath Hospital, May 6th, 1831.

Urine, Sp. Gr., 1036.25.

| | |
|---|---|
| Water | 913 |
| Sugar | 60 |
| Urea | 6.5 |
| Salts, &c. | 20 |
| | 1000 |

A man in Sir Patrick Dunn's Hospital, clinique of Dr. Graves:

Urine, Sp. Gr., 1033.

| | |
|---|---|
| Water | 929 |
| Sugar | 51 |
| Urea | 5.3 |
| Salts, &c. | 14.7 |
| | 1000 |

A man in the Meath Hospital, May 27th, 1831.

Urine, Sp. Gr., 1050.5.

| | |
|---|---|
| Water | 880.5 |
| Sugar | 70 |
| Urea | 13.5 |
| Albumen | 3 |
| Salts, &c. and loss | 33 |
| | 1000" |

Mr. Kane also made some experiments on the blood of diabetic patients. He could detect no sugar whatever in it, although five grains of sugar, dissolved in water and mixed with a pound of blood previous to coagulation, were afterwards readily discovered in the serum. The following is the analysis of the blood, the quantity operated on being in no case less than eight ounces.

"A man in the Meath Hospital, clinique of Dr. Graves.

Sp. Gr., of Serum 1030.

The blood was composed of

| | |
|---|---|
| Water | 798.13 |
| Fibrine | 2.18 |
| Hematosine | 114.50 |
| Per-oxide of iron | .45 |
| Albumen | 50.13 |
| Phosphuretted fat | 1.70 |
| Osmazome, Salts and loss | 33.21 |
| | 1000. |

Mackay, Sir Patrick Dunn's Hospital.

Sp. Gr. of Serum 1029.75.

| | |
|---|---|
| Water | 781.0 |
| Fibrine | 2.9 |
| Hematosine | 130.4 |
| Per-oxide of iron | .56 |
| Albumen | 45.5 |
| Phosphuretted fat | 2.0 |
| Osmazome, Salts and loss | 37.64 |
| | 1000.00" |

We do not profess to offer an opinion on these chemical researches. The subject is highly deserving of attention, and will probably receive it from those who are qualified to pursue the investigation.

### LXIX.

**Observations on some Diseases of the Vascular System. Essay I. Diseases of Veins—Essay II. Diseases of Capillaries. By T. A. Wise, M.D. Bengal Medical Service.**

We notice this pamphlet, in order to do an act of justice to an able and zealous member of our profession. From the facilities with which medical men residing in London, or the large cities of the empire, can lay their thoughts or their experience before the world, and from the rapidity with which they are disseminated by the periodical press, an advantage is given them which can hardly be possessed by the practitioner in the country or the colonies. With such men, the results of experience are too often buried. Dr. Wise, for instance, the author of the Essays now before us, has anticipated Mr. Arnott in many particulars; but Mr. A. from his residence in the heart of the Empire, having got the start of our countryman in Bengal, the latter is not likely ever to recover the ground he has thus lost. The Essays in question were read before the Medical and Physical Society of Calcutta in 1828, but were not published in their Transactions on account of their length, and of the expense of two ordinary lithographic plates. If there is one thing dear to a professional man, it is his reputation; and it does seem cruel, that one who has laboured in an investigation should have any credit or emolument which may be due to him wrested from his grasp by another. Such is said to be the case in the present instance. In a letter accompanying the pamphlet, a charge is openly and broadly advanced against Mr. Arnott, of plagiarising and purloining the author's property. We are no parties in the quarrel—we espouse neither side; but, as this is a heavy accusation, it is imperative on Mr. Arnott to meet it and repel it. In order that he may see with what he is charged, we shall insert the statement of Dr. Wise as it is transmitted to us, and we entertain no doubt, from the high character which Mr. Arnott bears, that he can satisfactorily remove the impression which such a statement, uncontradicted, would be calculated to produce.

"When dissecting in M. J. Cloquet's private room at St. Louis, in 1825, M. Richerand was unfortunate in several cases of amputation, which I examined. The cause of death, in these cases, led me to attend to the subject of phlebitis; and I had soon after the pleasure of finding my opinions approved of by the celebrated Dr. Edwards, of Paris, and others. The year following, I held the appointment of house surgeon at St. Bartholomew's, London, where I had an opportunity of verifying many of my conclusions; but I was still anxious to have the opinions of some of those, whose candour, industry, and discrimination, I thought I might rely on. One of these gentlemen was Mr. Arnott, at that time engaged in collecting cases on erysipelas; and it is but justice to myself to affirm, that I spared neither time nor trouble, as far as my then extensive opportunities afforded me, of assisting him in his researches. In those frequent meetings, I drew his attention to the subject, which I had been studying with much care. I shewed him the cases I dissected, my drawings, and placed in his hands the result of my labours on the pathology of the vascular system. I did this, of course, with the idea that he would not make any unfair use of them, nor do I suppose that either of the other two gentlemen,* to whom I was equally candid, ever heard him hint that he had an idea of publishing them as his own opinions—cases to which *I* first directed his attention, in confirmation of conclusions I had formed from former experience.

However, a few months after I had bid adieu to my country and friends, amongst whom I unsuspectingly included him, he read his 'Pathological Inquiry into the secondary Effects of Inflammation of the Veins' before the Medico-Chirurgical Society; to which, he says, his 'his attention was particularly directed, by some occurrences which marked the course and termination of the fatal cases of inflammation of the veins, after venesection, which he had an opportunity of observing (vide London Med. and Phys. Journal, No. 38, page 138, new Series). Then follows the account of three cases, two of the first, and I believe the third, will be found related in the following Essay, and were those that I particularly shewed him as proofs of my opinions. In some parts, perhaps, there may be a slight difference of opinion: but I believe they are few, and on points of little importance. Mr. Arnott was evidently necessitated either to mention my name, or content himself with appearing first before the public under a peculiar designation, although he was well aware that my Essay had been ready for publication before he had even thought on the subject.

More I could say in corroboration of those opinions; but I am disgusted with the ungrateful subject, and leave my essay to the candour and discrimination of an impartial public."

We have not space to give any thing like an account of the paper in this part of the Journal, but probably we may be enabled to notice it more fully in our next number.

---

LXX.

### CASES OF ABSCESS PRESSING ON OR OPENING INTO THE VAGINA.

DR. DAVIS relates two cases of this description in Part VII of his Principles of Obstetric Medicine lately published. The first case occurred to himself. An intense inflammation of the left ovary terminated in an abscess so extensive as ultimately to involve the peritoneal flooring of the left lateral portion of the posterior chamber of the pelvis. The pain being chiefly referred to the hypogastrium and small of the back, an examination was made to ascertain the condition of the uterus. The orifice and neck were very tender to the touch, and a large fluctuating tumour was felt to occupy the left and posterior portion of the pelvic cavity, causing an extensive projection or convexity of the corresponding portion of the vaginal parietes towards the interior of the passage. The abscess, for such it was, was the result of a severe and much mismanaged labour. On examination of the body after death, the matter of the abscess was found to have made its

* "For obvious reasons I do not mention these gentlemen's names; but I am sure both of them will be happy to substantiate the facts, should that be considered necessary."

way through the peritoneum of the posterior chamber of the pelvis into the cellular structure connecting the vagina and rectum with the inferior portion of the pelvis on the same side. For the particulars of the second case Dr. D. is indebted to Dr. Stroud.

"Elizabeth Hunt, a married woman, fifty-three years of age, was admitted a patient of the Northern Dispensary under the care of Dr. Stroud, about two months before her death. During the previous four or five months she had suffered severe pains, first in her feet, afterwards a little above the right knee, and ultimately in the neighbourhood of the right hip joint. Her health was already much broken, and she was very feeble and emaciated. She was lame from the state, as supposed, of the hip-joint. Her appetite was greatly diminished. Her sleep was disturbed, and she was the subject of a slight hectic fever. During the patient's first visit to the Dispensary, she assigned no cause for her complaint except cold, and fatigue from laborious occupations. But during her second visit it was ascertained that, some months before, there had been a discharge of blood from the vagina, accompanied by a fainting fit, and that, subsequently to that period, blood had occasionally issued either from the vagina, or from the rectum. The alvine dejections were said to be contracted and of a flattened form. Under these circumstances an examination per vaginam was proposed, and with some reluctance permitted. 'Two fingers introduced into that passage, although with the greatest caution, gave much pain, and were withdrawn covered with blood. A hard round substance, supposed to be the uterus diseased and displaced, was found near the orifice of the vagina, projecting backwards in the direction of the rectum. On account of the tenderness of the parts, and the great weakness of the patient, a more accurate examination was deferred to a future opportunity. In the mean time the suspicion of a malignant disease of the uterus, which had been previously entertained, was now considered to be confirmed."

The patient died, and a very minute and unnecessarily prolix account of the dissection is appended. We may as well take this opportunity of protesting against the long list of nothingnesses which it seems the present fashion to cram into relations of cadaveric alterations. If disease of the uterus is to be described, the writer begins with the crown of the head, and tells of every thing healthy or unhealthy, natural or unnatural, that presents itself from that starting point till his arrival at the Ultima Thule, the sole of the foot. With laudable liberality all receives the same degree of attention, and whether we are told of the condition of the soundest organ, or of that which was chiefly diseased, the same amount of labour, and nearly the same amount of words is expended, and the weary reader wonders to which the author attaches most importance. It is really abominable that in order to learn the nature of a disease in a single organ or apparatus, one must wade through a dissertation on every other to be met with in the body. The case before us will be shewn to be one of psoas abscess, ulcerating into the vagina. Then what in the world can such information as the following be worth, or why should it be here unless to exhaust the reader's patience, and make him exclaim against morbid anatomists as absolute nuisances. "The cartilages of the ribs were very short, and the ensiform cartilage was extremely narrow. The heart was quite sound and contained recent coagula on both sides. The left auricle was rather large and the right ventricle small. The venous trunks were full, and the arterial ones empty. The coronary vessels, particularly the veins, were beautifully injected, embossing as it were the surface of the heart, with abruptly serpentine lines," and so on. What interest or utility can possibly attach to this? We might as well be amused with a geographical and statistical account of Peru, before entering on the description of a psoas abscess. We do protest most earnestly against such a system of case making and case taking, and when samples come before us we shall not fail to visit them with heavier censure than we deem it necessary to resort to at present. To pass to the principal pathologic features of the case—the right psoas muscle was found excavated by a very large abscess which extended outwards to the groin, and downward to the bottom of the pelvis, where it had formed one considerable opening into the vagina and another into the rectum. The cyst was filled with greenish pus and flakes of lymph, which had found their way into the vagina and rectum and prevented the escape of the pus. Some of these flakes were found quite putrid in the vagina. There was a small distinct abscess on the left side of the rectum and rather behind it. The cavity of the large abscess was traversed from above downward by the anterior crural nerve, which was entirely denuded, and at one point seemed contracted and almost reduced to a membrane, and above and below became suddenly large and dense. The bone at the sacro-iliac synchondrosis was denuded. The cellular tissue at the posterior part of the vagina, contiguous to the peritoneum was infiltrated with lymph and pus, the peritoneum presenting some adventitious bands. The posterior half of the vagina, immediately below the neck of the uterus was destroyed by an ulcer an inch in breadth, by which it communicated laterally with the cellular tissue alluded to, and inferiorly with the rectum by an opening large enough to admit the thumb. The posterior lip of the orifice of the uterus was to a certain extent destroyed by ulceration. The rectum at the perforated part was thickened, and the mucous membrane in front of it red and swollen. These are the essential particulars. The case was one of psoas abscess, and the changes about and in the parietes of the vagina and rectum were consecutive.

---

### LXXI.

### CASES OF CONTRACTION OF THE VAGINA.*

IN that portion of his work which treats of imperforate, obstructed, and contracted vaginæ, several interesting cases are related by Dr. Davis. From these we shall select two which occurred to himself, and are not uninstructive. The first is an instance of general, the second of partial contraction of the vagina. The first is so brief that we give it in the author's words.

*Case* 1. "Its subject was a lady of thirty-nine years of age, whose spine was considerably distorted by an injury which she accidentally sustained during her infancy. When she had been in labour only for two or three hours, she was advised by her monthly nurse, who probably anticipated more than ordinary difficulty, to send for her medical attendant. On the author's arrival, he found the orifice of the uterus very amply dilated, NEARLY OBLITERATED, and the fœtal head presenting favourably. In ascertaining these facts, however, he encountered some difficulty in carrying his finger through the middle of the vagina, where he found it contracted into a very narrow diameter, and presenting such a feel as it might have been expected to present, if it had been bound by a ligature coiled round it on the outside It moreover felt firm, thick, and rigid. The mother of the patient, being in attendance, reported that her daughter had always menstruated regularly and well, and that she had never been the subject of any known disorder of her genital organs. The affected part gave no evidence of its ever having been the subject of cicatrisation. Time and patience, therefore, presented themselves as the principal remedies. The cavity of the pelvis was sufficiently ample. The labour-pains became very active in the course even of a few hours, and ultimately exceedingly urgent, accompanied by a tempestuous excitement of the

* Dr. Davis's "Obstetric Medicine." Part VI.

heart and arteries. The patient was bled freely and repeatedly, and the hand was as much used to promote dilatation as was deemed consistent with the soundness and safety of the part to be dilated. She was delivered of a still-born child in about FIFTY HOURS after the commencement of the labour. She recovered slowly but perfectly. She sustained no retention of urine, nor purulent discharges during her convalescence. The loss of an heir to a good property, as it might well be supposed, was not a little regretted. But the disappointment was forgotten, and the loss doubly repaired by the subsequent birth of two living children, both sons."

In some cases of contracted vaginæ a considerable, and we may say, sudden dilatation occurs during labour, of which the following is not a bad example.

*Case* 2. The wife of a corn-chandler had a first labour of long duration, and attended with great excitement of the heart and arteries, and during her recovery she was subject to severe purulent discharge. In the next labour, another medical practitioner was employed. On making the usual examination, he found the vagina, about an inch and a half from its orifice, so contracted that he could not pass his finger through the narrowed portion. He ordered the patient a dose of castor oil: and, as it was early in the labour and late in the evening, he left her till the following morning. He then found that the pains had been neither frequent nor urgent; the contracted part of the vagina was somewhat less rigid, and, on one side of the ring, it was distinctly soft and relaxed. Nothing further was done till the following evening, when the labour became more active and the circulation began to be excited. The morbid ring was dilated to the size of about half a crown, continuing rigid, tuberculated, and thick on one side—soft on the other. The medical practitioner employed a full bleeding, exhibited forty drops of Battley's sedative, and remained with the patient during the night. Early next morning he sent for Dr. Davis, who did not arrive till 3, p.m. The orifice of the uterus was now dilated to the diameter of two inches and a half, the head of the child had descended nearly half way into the pelvis, *the membranes were unruptured*, the left side of the vagina felt hard and greatly thickened, but the other was soft and disposed to dilatation, when the child's head was propelled by the uterine contractions against it. The excitement of the heart and arteries was considerable, the skin moist. A pound of blood was abstracted. At nine, p.m. the symptoms were more formidable; the arterial action was very great, the countenance expressive of great suffering, the patient extremely restless. The liquor amnii had escaped for several hours; but the descent of the fœtal head had made great progress, one-third of it being in the act of clearing the impediment of the left side of the vagina. There was doubt as to what should be done; but, abandoning a resort to the forceps or the knife, it was determined to try the effect of another bleeding. The patient was accordingly bled to faintness (thirty-two ozs.) and with success. The excitement was allayed, and the patient, after sustaining some fifteen or twenty more powerful pains, was delivered of a living and well-grown child. Her recovery was speedy and perfect, and her family now consists of four children, of which the three younger have been born at the eighth month of gestation, in consequence of premature labour having been purposely induced in the pregnancies subsequent to the one we have described. All the labours have been extremely slow, and the two last rather alarmingly severe. The child whose birth forms the subject of the present case came into the world, like Tristram Shandy, with his head rather the worse for its voyage. It was considerably disfigured by the pressure it sustained in its passage through the narrow part of the vagina, and presented on its left side a furrow, long, broad, and deep, with some abrasion of the cuticle. We know not whether the father took the matter so much to heart, as did Mr. Shandy the flattening of Tristram's nose, by the newly-invented and all-but-perfect forceps of Dr. Slop. But this by the way.

"A case, bearing something of analogy to the one just narrated, may be found recorded in the first volume, p. 461, of the London Medical Repository. But as the practice which was described by its contributor as having been resorted to for its relief, has always appeared to the author much too hasty, as well as prematurely vigorous, he cannot give it a place in the present work without introducing it to his reader by an expression of regret, that a case so peculiarly important on many accounts, so supremely easy apparently as to its management, and so strikingly felicitous in its results, should have been given to the public, without the benefit of the most ample authentication. 'A woman fell into labour of her second child. The vagina seemed to be imperforate. But on more attentive examination, an opening was found very near the perineum, which appeared not to lead directly to the vagina, but to pass at first sideway downwards towards the rectum. It would not admit the point of the little finger. On dilating this with the first finger, the child's head was felt, high up, pressing against the obstruction, which extended a considerable distance along the vagina in the form of general contraction of the canal, rather than obliteration from adhesions. It became necessary to DIVIDE MUCH OF THE SKIN LINING THE VAGINA, and AT LAST TO DELIVER with the forceps. After delivery, a mould of wax, in the shape of a long pear, was worn, and has prevented the accident from occurring again. In her labour preceding this, she had been delivered with the forceps after three days pain, of a dead swollen child, and for a fortnight afterwards the catheter was used for retention of urine. The contraction was discovered within five weeks after delivery.'"

Dr. Davis concludes the subject by the mention of a case, illustrative of what certainly appears to have been infamously bad management on the part of some surgeon in the country. The patient was a young woman who became pregnant soon after her marriage, and, while on a visit to her friends in the country, had a premature labour a few weeks prior to the natural period. She represented her medical attendant as having used instruments, and experienced great difficulty in effecting her delivery. On her return to town, after a tedious and painful recovery, the vagina was found to present a perfect cul-de-sac, at the distance of an inch and a quarter from its orifice, the whole being perfectly smooth, lined with mucous membrane, and free from rugæ. Sir Astley Cooper declined an operation, and the case is irremediable. From all that he could learn, Dr. D. thinks that the employment of instruments must either have been totally unnecessary, or, if required, that they were used most unskilfully. Can any thing be more reprehensible than such culpable ignorance on the part of a medical man.

---

### LXXII.

### CASE OF ABDOMINAL WOUND. By JOHN JAMES HALLETT, Surgeon, &c.

I WAS sent for, on the 16th ult., to visit E. Brereton, a young man residing with his father, at about a mile from this place, and found him labouring under symptoms of mental derangement, which, however, were not so apparent at the time of my seeing him, as, from the account given me by his friends, who represented him as having used violent and unjustifiable threats to his parents, although naturally of an inoffensive disposition. His appetite, also, had fluctuated; for at some times he would refuse food for many hours, although a labouring man—at others, a moderate allowance for three or four men would not suffice to answer his cravings. He complained to me of a sense of deep-seated heat in his abdomen, about the epigastric region, with slight tenderness. He was very shy of being questioned, and refused any and every medicine; yet said that, if he could make up his mind in a day or two to take any, I should be sent for again. On the Monday following (the

21st), I was called in a great hurry, at 6, p.m., to attend him, as he had attempted suicide by plunging a knife into his abdomen. He had had one knife taken from him by his father, and another by a cottager, half a mile from his home, where he had run to on being interrupted at home; and it was with the third that he succeeded in producing the wound. I found him on his back, with an irregular, ragged wound, the direction of which was oblique, with the umbilicus for its centre. The extremity on the right side was the highest. There was a small tongue of integument and cellular substance at either end of the wound, presenting such an appearance, as induced me to imagine that the knife had been thrust in at the umbilical depression, drawn upward and outward, and then turned and pushed downward and to the left. My idea was confirmed in my own mind, by the appearance presented by the knife, which, when found, had a considerable quantity of fæces on its blade, near the point, and appearing to have been placed on it by a sort of turning or scooping action. This latter circumstance, coupled with the fetid odour emitted from the wound, amounted, I think, to almost the most direct evidence of a wound having been inflicted in an intestine. Lying on the abdomen, were portions of omentum and large and small intestine *inflated*, equal in contents to at least two quarts. The extreme extent of the wound was about three inches, which, from its nature and irregularity, possessed a capacity that it would not, had it been produced by a simple straight incision:—So much in favour of a replacement of the dislocated parts, though a larger portion had, of course, been protruded. Thanks to the jealousy which, I fear, actuates too many of our profession, having but recently established myself in this neighbourhood, circumstances conspired to render it necessary for me to undertake the case alone, which I did, and which I have had no cause to regret. The displaced intestines were but slightly if at all discoloured, and I contrived, without much delay, to return them in that order which I conceived necessary to securing a return of function. I applied a light dressing, with numerous broad and long cross straps of adhesive plaister; and over all a roller of linen, four times round the abdomen. For some hours my patient was tranquil; yet, aware that circumstances might arise which might tend to mischief, I remained all night, and it was fortunate that I did, for, about midnight, he struggled so violently with the abdominal muscles, that two knuckles of large intestine were thrown out between the sutures of the wound, of which I omitted to state I had applied three. The difficulty which I had now to contend with, was greater than that which was presented in returning the original mass. I first attempted to divide the sutures, but they were so imbedded beneath and among the convolutions, that the extreme risk of wounding the intestine induced me to desist. By dint of perseverance I at last succeeded, by gentle pressure, to return them again, though not until they (the intestinal convolutions) were more discoloured than I liked; they had also become perceptibly tender to the patient. After this all went on well.

Having learnt that his diet, for some days, had consisted of a small quantity of coarse bread daily, and that he had tasted nothing on the Monday, I would not bleed him, but determined to watch him closely, and do so should a shadow of excitement appear. On the second day the bowels acted four times, after taking two small doses of magn. sulph. and nothing occurred to excite my attention till the Sunday after the occurrence, when the patient complained of a slight tenderness in the region of the crest of the right ilium. The application of a dozen leeches quite removed this; and, as a measure of precaution, the leeches were re-applied in two days after. He has taken pulv. antim. gr. ij., c. ant. tart. gr. $\frac{1}{15}$ in a powder, and followed by tr. hyosc. ʒss., and liq. ammon acet. ʒij., in a draught every six hours.

The wound filled up early, and has now healed. It will be necessary, however to remove him to an asylum, which will be done on Monday next.

There has been no recurrence of the tenderness.

---

### LXXIII.

### FATAL CASE OF COMPLETE CYSTOCELE, OR HERNIA OF THE BLADDER. By MR. CLEMENT.*

THOUGH several authors of reputation have mentioned cases of hernia of the bladder and offered remarks on its origin and nature, they are still sufficiently obscure to make any additions to our knowledge of them desirable. The following case is related by Mr. Clement, and is equally interesting and instructive.

*Case.* Mr. Bowley, æt. 60, very corpulent, had been affected with scrotal hernia on the left side for five and twenty years. The hernia at first was very small, but had slowly increased until it attained a magnitude that will presently be described. The chief inconvenience experienced was from its great bulk and weight. He repeatedly, however, suffered from constipation of the bowels and slight attacks of hemiplegia. He could never make water without first raising the pendulous rupture from between his thighs towards his belly, when, after rolling it about for a short time, the urine would pass in a full stream, though never in considerable quantity at one time.

A fortnight prior to his death the bowels became obstinately constipated, and he was attacked with paralysis of the left side of the body. The more urgent symptoms of strangulation of intestine were absent, but there was constant stillicidium urinæ. The catheter was used several times by two surgeons in attendance, who believed that it passed into the bladder; not more than a tea-cup full of urine followed each introduction of the instrument. Strong purgatives and enemata were employed; the stillicidium urinæ was supposed to be dependent on the paralytic affection. No alvine evacuation was procured, yet the patient laboured under symptoms of retention of urine rather than strangulated gut. There was great pain about the pubes and in the rupture, which became more distended—articulation became indistinct, and the patient expressed his feelings only in a low muttering tone—he became delirious—and died. Such is the imperfect but correct outline of the case. Mr. Clement was first present at the examination of the body.

*Cadaveric Examination* 24 *hours after death.*—"The circumference of the rupture was two feet five inches; its greatest length, measuring in a direction over the pubes to the apex of the tumour, one foot two inches and three quarters. The whole of the penis was retracted within the integument covering the rupture; the opening through which the urine flowed very much resembled the navel, and this gave to the tumour the appearance of an immense umbilical hernia, extending over the pubes and falling down between the thighs. One of the testicles could be distinctly felt near the surface, about the middle of the tumour, but the other was not discoverable before the parts were dissected.

Although the rupture was so large as to extend generally over the pubes, and occupy both inguinal regions, it was easily ascertained, without making any incision, that the protruded parts came down through the left abdominal ring.

The examination was commenced by dissecting for the left inguinal canal, which was exposed, and part of the colon found passing through it greatly distended with feculent matter, but exhibiting no marks of inflammation or strangulation: the latter could not have taken place, the passage being so much dilated as to give the whole hand free admission into the cavity of the abdomen.

A semicircular cut was next made through the integument, following the course which the colon had taken downwards in the sac: this incision was continued some inches in length, when one

---

* Observations in Surgery and Pathology, pp. 144, et seq.

of the testicles was exposed, and we were surprised by the sight of another distinct sac, very tense and containing fluid. By this we were led to suppose, that some portion of the intestine must also have protruded through the abdominal ring on the right side, forming a double kind of hernia. This part was again carefully examined, but no intestine could be traced to that side: besides, the surface of this second or supposed sac was too regular to contain either omentum or intestine; it formed the greatest bulk of the whole tumour, and more resembled a hydrocele, if it was possible to conceive one to attain a size so immense. By dissecting the integument from the surface, the apex of this second sac was found to be very thin, red, and pointed —in fact, appearing ready to burst: this inflamed part was accidentally ruptured, and about two quarts of very fetid urine was evacuated, which removed all doubt and previous uncertainty as to its nature.

By following down the course of the urethra, which was wonderfully displaced from its natural situation, we discovered that the bladder of urine had protruded through the abdominal ring. Owing to the prejudices of the patient's friends, all the parts could not be brought away, so as to make a complete preparation shewing the relative situation which they observed to each other. The bladder was however removed from the body, and, upon examination of the prostate gland, which was much enlarged, I found that it had been perforated by the catheter, in the ineffectual attempts made to draw off the urine. The diameter of the ureters was so much enlarged, as to admit my fore finger with great facility.

The abdomen presented no marks of acute inflammation having recently taken place: the omentum was much loaded with fat, and the whole length of the colon was greatly distended with feculent matter.

In the pelvis there was nothing remarkable, excepting the want of the urinary bladder; the natural connexion which exists between that viscus and the inner surface of the pubes, was not to be discovered."

Cases like these are only useful when they afford a profitable lesson or serve to inspire future caution. There are several circumstances in the history of the case which might perchance have afforded a clue to its nature, if presented to a well-informed and reflecting surgeon. The long continuance of a large hernia unaccompanied with much disturbance of the intestinal functions, the occasional attacks of retention of urine, the mode in which the bladder was most easily emptied, the character of the final and fatal attack, might possibly have led an acute observer to suspect the real nature of the hernia, or will serve to do so to those who read the present particulars, and chance to meet with a similar case hereafter. We are not informed that the size of the hernia was diminished after the passage of urine, a circumstance generally, we believe, remarked in the recorded cases of hernia of the bladder. Mr. Clement adds some reflections on the mode in which hernia of the bladder takes place, but this is a topic on which we need not dwell. It is of more importance for our readers to be aware that the bladder having passed wholly or in part into the inguinal canal, a pouch is formed above by the peritoneum accompanying the viscus, into which pouch omentum or intestine, or both may descend, or even be accidentally strangulated. We have noticed this case to put practitioners on their guard, for although the knowledge of rare cases is of less importance than that of diseases of common occurrence, yet a surgeon from ignorance of the former may lose his patient and endanger his professional character.

---

### LXXIV.

### Mr. Lizars' Letter on Cholera.

To those acquainted with the professional zeal and professional acquirements of Mr. Lizars, any mention of them would be superfluous, and we need not therefore say that the letter before us is both instructive in a practical, and interesting in a physiological point of view. The letter also derives an additional degree of interest, from its having been the occasion of a personal attack on Mr. Lizars by some individuals in the Edinburgh Board of Health. An attack so ungentlemanly, so uncalled for, has seldom been witnessed of late years, and the general feeling of indignation which it aroused will prove we hope an ample guarantee, against the repetition of similar outrages from authority on common decency and common sense. Mr. Lizars may be persuaded that however galling such a scene may be to his feelings as a private individual, he has gained, not lost, in his public character. The spirit which could dictate in these days wanton insult on account of a professional opinion, is a lineal descendant of that which in darker ages could raise the Smithfield fire for religion, and persecute Galileo for science.

The first part of the paper contains a letter to Mr. Lizars from the able Professor of Montpellier, M. Delpech. Our readers are probably aware that M. Delpech conceives the semilunar ganglia, and abdominal plexus to be the principal and essential seat of the morbid action of cholera, and that he has found his ideas confirmed by numerous dissections. To establish this point is the main object of the present letter. We have already dedicated so much space to cholera, that we cannot enter into it again. Mr. Lizars is an anticontagionist, and portrays in a manner equally argumentative and eloquent the absurdities into which contagion has led both Boards and individuals. That doctrine is now so generally abandoned that we need not discuss it here. Yet we cannot refrain from mentioning one instance of the cruel folly to which it has given birth in Scotland. While cholera was at its acmé at Fisherrow, *and when the most unrestricted intercourse by land and by foot passengers was allowed*, a solitary boat with two men from the place touched at Leith harbour. The poor fishermen had no sooner landed than they were hurried away under guidance of a guard-boat to perform quarantine at St. Margaret's Hope! We can scarcely believe that men in the possession of their senses could be guilty of such absurd injustice. We will not say with Fouché that such conduct is "worse than a crime—it is a blunder;" but assuredly it is both a crime and a blunder. As a matter of rational argument it cannot admit of defence, but when the injurious consequences upon a population already pauperized and ruined are considered, we shudder at the levity of men who can rashly deal abroad destruction, with such indifference and indiscretion. The following quotation is the only one we shall make from Mr. Lizars' pamphlet. It is obviously insusceptible of abbreviation, and yet contains a very valuable digest of the morbid anatomy of cholera. It is a "summary of twenty dissections."

"Brain.

Of this number twelve had this organ examined, and, in all, the arteries and veins of the integuments and muscles covering the cranium were distended with the dark blood, which, in some flowed like tar.

In ten, the blood-vessels of the dura mater were turgid with this blood; and in three, there were fibrinous coagula.

In seven, there was serous effusion under the arachnoid membrane.

In four, the pia mater was congested with blood-vessels.

In seven, the cerebrum was highly vascular; and in one, slightly softened.

In seven, the cerebellum was very vascular; and in three, its substance was slightly softened.

Spine examined in ten.

In six, serous effusion between theca vertebralis and arachnoid membrane, and, in one of these, the fluid was bloody.

In two, serous effusion between arachnoid and pia mater.

In six, blood-vessels of spinal chord highly injected with the dark blood; and one with evidence of inflammation between dorsal and lumbar regions.

In six, the spinal or rachidian veins turgid with dark blood.

GANGLIONIC SYSTEM EXAMINED IN SEVENTEEN.

In ten, the neurilema of pneumogastric nerves was injected with blood-vessels; in one, the nerve was enlarged; in another, it was thickened; and in a third, the neurilema was inflamed with ecchymosed patches.

In six, the neurilema of splanchnic nerves was vascular; in two, the ganglia at their origins were vividly injected; and one ganglion was ecchymosed.

In sixteen, one or both of the semilunar ganglia were vascular; in one, it was inflamed; in three, it was enlarged and infiltrated with blood or serum; and in two, softened.

In eight, the solar plexus highly vascular throughout; in three, the ganglia and nerves enlarged, and one infiltrated.

In four, the renal plexus was very vascular.

In four, the œsophageal plexuses were vascular.

In one, the recurrent of the pneumogastric nerve was vascular.

In five, the cardiac plexus was enlarged, and very vascular.

THORAX.

*Heart.*—In three, the heart was flabby and pale; in two, collapsed; and many of them had the left ventricle so contracted and firm, as to contain only a drachm of blood. In thirteen, the right side was full of the dark gory blood, part of which was generally in the state of a fibrinous coagulum.

In three, the left side was full of the same blood with coagula.

In three, the right auricle full of dark blood and coagula.

In six, the left auricle full of dark blood and coagula.

In four, left ventricle was moderately filled with blood and coagula, and one affected with softening: in two, coagulum extended into aorta.

In five, right ventricle full of blood and coagula. In one, coagulum extended into pulmonary artery. In two, the parietes were softened.

*Pericardium.*—In one, this sac was distended with gas; in two, it was dry, like paper, and vascular; and, in a third dry, vascular, and diaphanous. In four it was vascular. In all, the coronary vessels more or less injected with dark blood.

*Venæ Cavæ.*—In all, more or less of the dark blood was found.

*Pulmonary Veins.*—In six, these veins were turgid with the dark blood.

*Lungs.*—In four, these organs were congested with the dark blood.

*Pulmonary Artery.*—In one, a large coagulum, which extended into its two large branches. In three, it was full of the dark blood; and, in three others, the vasa vasorum were highly injected.

*Pleura.*—In five, highly injected; and in two, there was effusion of lymph.

*Aorta.*—In all, it contained more or less dark blood, with fibrinous coagula; in six, the vasa vasorum were highly injected,—the dark blood, and occasionally coagula, extended into the carotid, brachial, femoral, tibial, ulnar, and radial arteries.

ABDOMEN.

*Peritoneum.*—In nine, this membrane was highly injected; in six, evidently inflamed; and in three there was albuminous effusion, with some turbid serum. In one, the omentum was very vascular; and in another, it was inflamed.

*Stomach.*—Generally of a white colour both on its peritoneal and mucous tunics, and containing more or less of the rice-water fluid.

In seven, there were distinct vascular patches on the mucous coat, with several ecchymosed spots, varying in size, from that of a sixpence to that of a half-crown; and in all, there was manifest softening. In one, the mucous tunic was eroded.

*Small Intestines.* — In twelve there were evident marks of high inflammation, and vivid and extensive injection; in nine, ecchymosed patches; in four, mucous tunic softened in many points; and in one, incipient ulceration. Contents of a viscid white mucous, or greenish colour; and in two, they were bloody.

*Large Intestines.* — Transverse arch and sigmoid flexure of colon, commonly spasmodically contracted. In five, vascularity, with ecchymosis. Two inflamed, with softening; and one with ulceration. Two with dark venous congestion, similar to intestine in strangulated hernia.

Contents generally rice-watery, or gruelly and flocculent, occasionally greenish and viscid. In many, the colon, with the exception of the caput cæcum, was empty.

*Liver.*—Very various in colour; two with Bright's yellow deposit. In some, the vena portæ were moderately congested; and, in one, the biliary ducts full of bile.

*Gall Bladder*.—Generally two-thirds full of rather inspissated olive green bile. In the twenty cases, ten were full of this fluid; the others varied from a little bile to two-thirds.

*Pancreas*.—Generally healthy.

*Kidneys*.—Commonly healthy; but varying, like the liver, according to the habits of the individuals. Three were slightly congested; one gorged with the dark blood; and another presented a livid appearance.

*Urinary Bladder*.—In all, contracted, and almost empty. When any fluid was present, it was about a drachm of muco-purulent. One, however, was contracted horizontally, and contained five ounces of limpid urine."

---

### LXXV.

### Aneurism by Anastomosis, requiring Extirpation.*

Anne Evans, aged nine months, was brought to Mr. Clement, at Shrewsbury, with a tumour on the forehead, resembling in all respects the "aneurism by anastomosis." It was very prominent, oval, rather larger than a shilling in circumference, of deep purple colour. Under gentle pressure, it communicated a vibratory pulsating feeling to the finger: but, under firm compression, this sensation ceased. Four large tortuous arteries ran superficially into the substance of the tumour at different angles. At the birth of the child the tumour was only half so large; it had increased with great rapidity during the last two months. Mr. Clement determined to tie the vessels leading into the tumour. He placed ligatures round the two upper, which pulsated strongly, and were nearly as large as crow-quills; but the child struggled so violently, that he deferred securing the two lower. Little change, save a diminution of the force of the pulsation, was produced in the tumour. On the following day, Mr. Clement secured the two inferior arteries. The pulsation of the tumour ceased entirely; it became more tense and of darker colour, and seemed ready to burst. On the next day it was much softer, less prominent, and not so livid; and, on the second day, it was quite flaccid, and scarcely darker than the integument around. The ligatures separated on the fifth day, the tumour having become flaccid and barely discoloured. On the second day after the separation of the ligatures, the purple colour and pulsation of the tumour had returned, and in nine days, it was as large and discoloured as before the operations; the four large arteries were not perceptible, nor was the tumour so prominent. Mr. C. now excised the tumour. Although he made the incisions at a considerable distance from the base of the tumour, where the integuments did not appear unusually vascular, the hæmorrhage was very alarming. The child was much exhausted, but recovered perfectly in the course of a few days, though the cavity of the wound was so deep, that nearly seven weeks elapsed before it was filled up with healthy granulations.

Mr. Clement makes some remarks with which we do not altogether coincide. He observes that "every other method, excepting that of complete extirpation, has generally failed in curing this affection." If by extirpation Mr. C. implies excision, the observation is incorrect; for the ligature is employed more generally than the knife, and at least with equal success. We have repeatedly seen these nævi and aneurisms by anastomosis cured by the ligature. A small nævus may be tied by simply passing two double ligatures, crossing each other at right angles under the base of the tumour, and either strangulating each half by making two knots, or including four portions of the tumour, by separately tying each end of the two double threads. If the nævus is of large size, or if the case be one of aneurism by anastomosis, which is nævus of a larger growth and affecting the vessels of the cellular tissue, then

---

* Mr. Clement's Observations in Surgery, &c.

the employment of the ligature, in the manner recommended by Mr. Brodie, is perhaps the best method. Mr. B. passes two long needles, which cross each other at right angles beneath the base of the tumour, and under these applies a circular ligature so tightly as to strangulate it. He has operated successfully in this manner on several cases. The first case in which he employed it is related in a recent volume of the Medico-Chirurgical Transactions, and was copied into this Journal. The most perplexing cases of aneurism by anastomosis, are those in which a large extent of surface is irregularly affected. A boy, for instance, was recently in St. George's Hospital with an enlargement of the blood-vessels of the left cheek, angle of the mouth, angle of the orbit and probably part of its interior, and temple. The pulsation was indistinct, the disease, according to the mother's account, on the increase. It was not deemed prudent to attempt an operation.

---

### LXXVI.

### Lithotomy in a Female Child.*

A female child, five years of age, was brought into the House of Industry, at Shrewsbury, in a state of great debility and emaciation. She complained of constant pain in her belly, cried violently when in the act of making water, and had much excoriation of the external organs of generation, and of the skin between the thighs. This latter was removed by proper applications, and Mr. Clement was induced to examine the child's bladder, which he did with a common probe. He discovered a stone, which appeared to be one of such size as to preclude the idea of its being capable of removal by any dilatation of the urethra. Mr. C. however, attempted to dilate it so far as to introduce a pair of forceps, and crush the stone. This was found to be impossible, on account of its extreme hardness, and the child suffered so severely, that Mr. C. determined to perform lithotomy, which he did on the 25th July.

"The child being placed in the same position as for lithotomy in the male subject, I first introduced Sir Charles Bell's largest sized male staff into the bladder, and placed it upon the stone, giving instructions to my assistant to retain it in that situation. With a large scalpel, I made a long and free incision along the side of the vagina nearly down to the anus: the point of the knife was then passed into the urethra and carried onwards to the neck of the bladder, when the urine gushed out in a full stream. I next introduced the fore-finger of my left hand into the bladder, and felt the calculus;—after ascertaining that the incision was sufficiently large to admit of its easy extraction, the forceps were introduced (my finger acting as director for the blades), and a stone larger than a pigeon's egg was immediately extracted. A piece of oiled lint was placed lightly in the wound, and the patient removed to bed.

The performance of the operation occupied only a few seconds, and the child apparently suffered less from the incisions than from the previous attempts which were made to seize and break the calculus.

It would be useless to follow up and to describe minutely the details of this case; I shall, therefore, only observe, that in the course of sixteen days the wound was perfectly healed, and the child able to run about, suffering no inconvenience from the operation excepting the want of power to retain the urine.

She was taken from the House shortly afterwards, by her parents, and I lost all sight of her until last year, when I was happy to find that the stillicidium urinæ no longer continued to trouble her. The bladder, however, continues irritable, for the patient cannot allow any large quantity of urine to accumulate in it. In every other respect, she is perfectly well."

---

* Mr. Clement's Observations in Surgery, &c.

LXXVII.

### Excerpta Choleralogica.

### I. IMPORTATION OF CHOLERA.

It is not a little remarkable that the introduction of this mysterious disease into each city and place can be clearly traced—when at a great distance—but never, when under our own sight. Thus if we ask the advocates of importation, how cholera was imported into Sunderland? They answer, we cannot tell; but we can shew you how it was traced from Jessore into Calcutta. How did it travel, man from man, to Newcastle? We know not; but we know how it travelled, by means of personal communication from Bengal to Bombay. Can you trace it from Newcastle to Haddington? No. But we can trace it from Bombay to Bagdad. From Haddington to Musselburgh? No. But from Bagdad to Ispahan. From Musselburgh to Kerkintilloch? No. From Ispahan to Moscow. Can you tell us how it was introduced into London? Oh no. But we can tell you how it radiated from the barques into St. Petersburgh. Such are the facts upon which the doctrine of importation rests. Our Hibernian brethren seem to be amazingly puzzled about the introduction of cholera into Dublin, as will appear by the following document, which deserves record, as it will prove of some importance, perhaps, hereafter.

CENTRAL BOARD OF HEALTH FOR IRELAND.

March 30.

"The Board were engaged on that day and yesterday in examining the different reports of the medical gentlemen who attended the cases, and hearing *viva voce* evidence on the subject, from all which it appeared that the symptoms of these cases very much resembled those described as attending the epidemic cholera in England, but tbe Board were not able to trace the disease to any communication by which it might have been introduced into the neighbourhood of Dublin."

The central Board ought to have provided themselves with some keen-scented cholera hunters from this side of the water, who would soon have found them a ship-scraper, a pedler, or a mendicant who came from some "infected district" of England, and carried the dire contagion with him in his wallet or knapsack. The Irish Board has shewn a lamentable lack of foresight on this occasion! P.S. As we expected they have here committed themselves. The disease has spread, and they themselves proved that it was not traceable to any contagion!

---

II. So too, cholera has taken the Gallic capital by surprise. Instead of landing at a sea port, as it is said always to do, and "travelling" by the great roads and channels of human intercourse, it has thought proper to step at once into the heart of France—aye, and into the heart of its capital, without paying the least respect to the documentary line of march chalked out for it "by authority"—and by the various historians who have traced its progress on the contagious route, from Jessore to Sunderland! This, certainly, is not as it *ought* to be! But so it is:—and it may give us some insight into the authenticity of those documents which have emanated "from authority," among the slaves of Persia, the hordes of Russia, the tongue-tied vassals of Prussia, Austria and other countries. The unfortunate bias which was given to the investigation in England, "by authority," has, for very obvious but humiliating reasons, cast a stigma on the medical profession of this country, which will require half a century to efface. True it is, that a few independenl and resolute minds have stood in the breach, and defended the ramparts of truth and science, when the battalions of contagionists and importers poured like a torrent upon the devoted citadel. They flinched not. They resisted the fearful preponderance of their countless adversaries, till reason dawned, and gave them powerful auxiliaries, from the very ranks of their opponents. The delegates of the "grand nation," and the quarantine restrictions of our Gallic, Spanish and Lusitanian neighbours, were cited as proofs that all nations regarded cholera as an indubitably contagious malady, to be stemmed and bounded by cordons and files of bristling bayonets! Let us hear then, what the first physicians of the "most civilized country" in Europe, say, when the epidemic is pouring into their hospitals.

"The undersigned physicians and surgeons of the Hôtel Dieu think it their duty to declare, in the interest of truth, that although up to the present time this hospital has received the greatest number of persons affected with the cholera, they have not observed any circumstance which authorises them to suspect that the disorder is contagious. (Signed)

| | |
|---|---|
| "Petit | "Recamier |
| "Husson | "Dupuytren |
| "Magendie | "Breschet |
| "Honou | "Gueneau de Mussy |
| "Samson | "Cailiard |
| "Gendrin | "Bailly |

"*Done at the Hôtel Dieu, Paris, March* 31, 1832."

Most of the other hospitals have followed the example of the Hôtel Dieu.

We shall not revert to the humiliating picture of our own professional magnates on a similar occasion. The large and black or red letters that *were to be* painted on the tenements of cholera patients, fortunately could not find painters—and London, that was confidently prognosticated to be a "*Charnel-house*" by the first of May, 1832, is now, (10th May) almost free from the pestilence! We will not be so uncharitable as to suspect that the prognosticators would have gloried in the accomplishment of their horrible anticipations; but, from what we have seen and *experienced* during the last three months, we have not the smallest doubt that, had the prediction been verified, the contagionists would have presented a petition to parliament, praying that the anticontagionists might be burnt, en masse, in Smithfield, "fire and ashes" being first sprinkled on their heads!

The proofs of cholera being a local epidemic, rather than a contagious disease, are daily multiplying. Thus the Duke of Orleans, the Archbishop of Paris, and many distinguished personages were in the habit of daily visiting the hospitals and conversing with the cholera patients, without catching the malady; while Casimir Perrier, busily employed in his cabinet, together with many others took the disease. This does not look like contagion. And when we find more than a thousand men stricken down in one day, and upwards of 400 of them perishing, in various parts of a great metropolis, can we possibly connect this with personal contact or contagion? But then some of the medical officers have died of cholera. Have they a special protection against an epidemic or an endemic influence? Why should not they be affected as well as Casimir Perrier, and several of the nobility of Paris? Were not the faculty, by their avocations, compelled to visit those localities where the primary causes of the epidemic were in greatest activity? Were they not obliged to spend great part of their time in the vitiated atmosphere of crowded hospitals, and the wretched apartments of the sick? Did a single physician or surgeon of a cholera hospital in London catch the disease, at a time when the talented and lamented Dr. Dill, of the London Fever Hospital caught the typhus and died of it?—That distinguished and gifted individual was busily employed in the investigation of cholera, visiting the sick, and dissecting their bodies—yet he caught—not cholera, but typhus, and fell a victim on the fifteenth day of the fever! Had he belonged to a cholera hospital; or had he caught this highly contagious epidemic, we should never have heard the last of this incontrovertible proof of the contagion of cholera.

---

### III. Ely Board of Health.

This hopeful scion of a celebrated stock in London and Edinburgh, has come to a most logical conclusion that cholera is contagious, "under certain circumstances," in the malarious locality of Ely. What are these certain, or rather uncertain circumstances adduced by the sapient Board of Cambridgeshire? *First,* some two or more individuals of the same house have taken the disease in succession—and *secondly,* individuals who came from a distance to attend the funerals of their friends in Ely, caught the disease there. Ergo, cholera is contagious. Let us compare this with some other similar circumstances and events. Several soldiers, in the same barrack, at Fort St. George, have become affected with hepatitis, in succession:—Secondly, some Europeans, who had just landed, and went to occupy the same barracks, became ill with hepatitis—ergo hepatitis is contagious. Now facts of the latter description are every day presenting themselves at Madras and other places, yet no one thinks of drawing the conclusion which the Ely Board of Health has drawn.

### IV. PROGNOSTICATIONS.

IT is well known that the Editor of this Journal hazarded a prophecy some eight or ten months ago, that, should the Asiatic pestilence reach our shores, it would be shorn of its terrors, and produce much less destructive ravages than in other countries through which it has travelled—or where rather, it has originated. The prophecy has, like many other prophecies, been partly fulfilled—partly falsified. The epidemic did *not* come shorn of its terrors—for the panic was universal. But the more important part of the prognostication was verified. The impression of the malady on the soil of Britain was extremely slight. It is sufficient to say that, during the first fourteen days of the epidemic in Paris, there died double the number that died in all England (including London, Edinburgh, and Glasgow) in seven months! We need say no more on this point at present.

### V. BARON HEURTELOUP ON THE PARIS EPIDEMIC.

WE quote the following passage from the Baron's letter to the Editor of the Times, 24th April, 1832, after the Baron's return to Paris.

"I cannot close this letter without saying a few words upon the important question of contagion; and I must here take occasion to affirm, contrary to the opinion of the contagionists, that notwithstanding the frequent contact between those suffering under the attack of cholera, with numerous individuals who have visited them in all parts of the city, not one single example of contagion could be cited. And if some persons, after contact with cholera patients, have been attacked by the malady, that must be attributed to the influence of general infection, and by no means to the communications which may have taken place between the healthy and the sick. Besides, the non-contagion doctrine, supported as it is by an infinity of proofs, has been proclaimed by all the physicians of the Hôtel Dieu, and by nearly all those attached to the other hospitals. The only question now is, to determine whether one or more cases of presumed contagion will pravail over an immensity of facts which prove the contrary, and whether there be not ground for this reflection—namely, that though the existence of contagion should be proved with respect to some individuals, it is necessary to take into consideration the frequency of its occurrence and the quantity of evil which may result from it,—in a word, to ascertain whether this quantity of evil is not inferior to that which is necessarily produced by sanatory measures. It would be a foolish attempt of the people of Paris to endeavour to escape (and the infection of Paris proves that such escape is impossible) from a less evil by submitting themselves to a much greater. Finally, it is bad logic that would lead to the conclusion, that to save 10,000 individuals from the risk of taking the cholera, 50,000 should be starved to death in consequence of the restraints imposed on commerce by sanatory measures. The whole question of contagion depends, I think, upon striking this balance.—I am, &c.

Baron HEURTELOUP.

### VI. MODIFICATIONS OF OPINION.

IT is well known that our contemporary, the LANCET, laboured most indefatigably in the cause of contagion:

and, from the circulation and influence of that Journal, we have no doubt that the subscribers to that doctrine have been centupled through the country, where written documents were all the evidence on which practitioners could depend; and where the *travelling* of the disease, by personal communication was made out most luminously. When, however, the evidence of the senses was thrown into the scale, the medical profession began to waver—and now, the advocates of contagion, as an essential character of the epidemic, are very few indeed.

We will allow our opponent on this point of controversy, to speak for himself; and give him much credit for the candour with which he avows a change, or very important modification of his opinions, though some of the reasons for that change are not to us very conclusive.

"In the interval that elapsed between the publication of this article, and the first appearance of the cholera in England, every feature in the disease proclaimed the absolute and direct operation of contagion. With the rapidity of an arrow it crossed by the Spree, and descended the Elbe to Hamburgh. Halting as it were here, it next broke out in one of our *maritime* ports on the coast opposite to that last infected. Here it dwelt for many days and weeks, and at last, step by step, and with all the characters of a disease still essentially contagious, it reached Newcastle and the adjacent villages, and lurked in them almost with the demeanour of contagious typhus.

Up to this period we maintain that to all reasonable men, the statistical history of the cholera was such as to permit no other rational conclusion than that it had reached England from the European continent by human intercourse, and had extended itself in England by that channel alone. Had the disease ceased at that time, *no other conclusion could have been adopted.*

But since that period the statistics of cholera have undergone the most complete alteration. In Great Britain it sprang up in Haddington, in Goole, in Hull, in Ely, in Rickmansworth, and in a dozen other places, which have little or no intercourse with each other, and with the fury of a West Indian hurricane, it has now burst out in Paris, in a few days sweeping off 20,000 victims to its rage. These facts again turned the scale, to shew us the operation of the unknown epidemic influence. They prove that infection is no longer the *essential* cause, and even tend to indicate that, for a time, its operation may be materially suspended. Such is the sole inference which an unprejudiced mind, anxious only for the truth, can derive from the contemplation of these events. If the operation of contagion was palpable in the transit of cholera from Berlin to Sunderland, the influence of some other cause is equally proved in the subsequent irruption of the disorder. The man who makes this inference changes 'no opinions.' Opinions are of a double kind, those founded on facts and reasoning, and those hazarded without a knowledge of either; rational opinions must undergo modification with the facts they flow from.

Even in the localities occupied by the disease, the most rigid scrutiny often fails to trace the operation of infection. In one district of London, thirteen families were, to our own knowledge, infected; but the first case having occurred in each without any appreciable infective cause, the other cases are inconclusive, as they might have originated in the unknown source by which the first was occasioned. This position is as clear as the construction of a common syllogism."

The Reviewer in the Lancet expresses his opinion that "*the presence of an epidemic cause* is, to a certain extent, a protection against human poison; or, in other words, that it is principally *in places free from the epidemic* that the disease can, under ordinary exposure, spread from person to person." This is strange reasoning. It amounts to this, that cholera is contagious when *sporadic;* but when epidemic, it loses that character! Valeat quantum!

The Medical Gazette also has been forced, by public opinion, to modify its doctrines not a little. While clinging to the doctrine that cholera *may be* contagious (which few have doubted), the learned Editor acknowledges that his "estimate of the *degree* of its contagiousness is very much reduced." "We had anticipated (says he) that, *when* the disease came to this country, the doctrine of contagion would have been extended and confirmed; whereas just the reverse has happened." This is candid, we acknowledge; but why the Editor of the Medical Gazette should lose his temper, and abuse all who held an opinion contrary to that which he is now forced to abandon, is not quite clear. It is certainly very impolitic in him to shew his anger so unequivocally and so frequently. It evinces a losing game.

We have received a letter from Surgeon Martin, of the 73d regiment at Malta, respecting cholera, of which he had ample experience during the years 1818, 19, 20, and 21, in India. The numerous facts and arguments which he adduces respecting the non-contagious character of the disease are now somewhat late, and the most strenuous advocates of contagion are now beginning to take rational and moderate views. We shall, however, extract a passage or two from the letter of our zealous correspondent. "Upon the subject of the disease being conveyable from one part of the globe to another by ships, &c. I never but once, during the above period, heard it even suggested. When the disease, of a very malignant character, first appeared at Point Pedro, in the Island of Ceylon, many vessels were daily clearing out for different parts of the coast, yet no disease was communicated to any of the ports to which they went—the cholera pursuing its own course uninterrupted along the coast of Jaffnapatam, and thence to Trincomalee, over an immense tract of jungle, where I witnessed its devastation, arriving at Ballacolloa, by such regular marches that its daily progress might have been laid down on a map before-hand." "I would ask the contagionists how it was that this deadly disease would make its appearance in a quadrangular fort, or the filthy narrow lane of a bazaar, attacking the occupants of only one face of the fort, or one side of the bazaar, leaving the remainder of the inhabitants, for that time, in perfect health." "While the 73d Regt. was drawn up one morning on the parade ground at Trincomallee, several men were simultaneously attacked with cholera, and were all dead and buried the same evening; yet not one of the others were seized with cholera at that time." Unfortunately the active depletory treatment recommended by Mr. Martin has not been found applicable to the epidemic of this country, for the reasons pointed out in our last number, especially the premonitory diarrhœa, by which the patient is too much depleted before that stage of the disease arrives which resembles the Indian cholera.

### VII. Cholera in Glasgow.

The contagionists are losing ground in every direction where the personal knowledge of cholera obtains. Thus at Glasgow, (as appears by the last number of the Glasgow Journal,) the doctrine of contagion has sustained a signal defeat. Dr. Auchincloss, in an able report from the "Town's Hospital," has clearly shewn that the disease arose spontaneously, not only in different wards of the hospital, but in different localities of the town, without any positive evidence of its being propagated from person to person afterwards. It appears that, in Glasgow, as elsewhere, diarrhœa was the premonitory or rather the primary stage of cholera.

In the review department the editors have taken the side of non-contagion, and our friend Mr. Moir, of Musselburgh, cuts but a sorry figure when his "proofs of the contagion of malignant cholera," are overhauled by the rigid censors of Glasgow. After citing some of Mr. Moir's "proofs," the reviewer remarks—"he (Mr. Moir) must presume his readers to be fools, or he intended to gull the contagionists, when he believed that even they would swallow such absurd nonsense."

### VIII. Proofs of Identity.

"The malady (Cholera in Dublin) has decidedly altered its type, since first *introduced*. Not one patient in thirty has it now in the original form. FEVER, with cramps, vomiting, and sometimes purging, is the form which at present is most prevalent, and it generally yields to antiphlogistic treatment."—*Medical Gazette*.

It will be remembered that the Irish Board of Contagion declared that the disease was not *introduced*, but occurred spontaneously; yet still the darling doctrine of importation is clung to with as much tenacity as their salaries! But now we find this specific disease which has *travelled* by means of personal communication from the Jumna to the Liffey, all at once changing its type, and becoming a gastric fever! Truly this is a strong proof of identity!

### IX. FUNDAMENTAL DOCTRINE OF CONTAGION.

Mr. ORTON is labouring hard in his vocation to disprove all he had formerly asserted respecting the non-contagious nature of cholera. We shall here advert to a curious circumstance which has escaped the notice of Mr. Orton's critics. It is well known that Mr. Orton stated in the Westminster Medical Society, that his opinions on the non-contagious nature of cholera were hastily formed, and that it was during leisure reflection afterwards in England he altered his sentiments. This might have been admitted had Mr. Orton cancelled a single leaf of the second edition of his work, which exhibits an exceedingly awkward disclosure. When Mr. O. had got as far as page 165, he refers to his Appendix, where he tells us that the balance of evidence will be found clearly *against* the infectious nature of cholera. But alas! between page 165 and page 313, certain circumstances occurred which entirely changed Mr. Orton's creed, and this very Appendix comes upon us crammed with arguments in favour of contagion, Mr. Orton forgetting to cancel page 165, where he refers to the Appendix with a totally different view!! What the reasons were which changed Mr. Orton's creed in the course of a few days, or at most, a few weeks, we are unable to divine. Perhaps the arguments in favour of contagion which emanated from the Board of Health just about that time, may have dazzled our author, and coming from his military superiors may have carried more weight than as emanating from other sources.

Be this as it may, Mr. Orton is now very hard pushed for arguments in favour of contagion, when he has recourse to the effluvia from the fæces of those labouring under diarrhœa, as the disseminating cause of cholera. The Parisian cholera is now clearly traced by Mr. Orton to a person with diarrhœa "who sleeps at Calais, infects the atmosphere of at least one public privy there, and perhaps transgresses the prohibition stuck up on the Boulevards, 'ni faire ni deposer ses ordures,' and passes on. The disease immediately afterwards appears there, &c."—risum teneatis amici?

And is all the beautiful chain of proofs which connected the cholera of Hamburgh with that of Jessore, now vanished into air—the mal-odorous air of *privies!* The dirty doctrine of diarrhœa is the last stay and hope of the contagionists! But even here, Mr. Orton has evinced his usual shortness of memory. It is not even a second-hand, but a third-hand doctrine. Dr. Whiting maintained it months before Mr. Orton wrote in the Medical Gazette (May 19th) and Dr. Kirk (page 14 of his work on Cholera) distinctly enunciates the doctrine which is claimed with such an air of originality by Mr. Orton. There is no doubt, however, that this *stercoraceous* hypothesis was hailed with delight in the *privy*-council of Whitehall.

### X. CHOLERA IN CORK.

Dr. D. B. BULLEN has recently published a short, but practical pamphlet on the epidemic, as it has appeared at Cork, of which we can take but a very brief notice. The disease first broke out on the 5th of April, in the person of a youth of eleven years of age, who died in about 13 hours. On the 13th and 14th of the same month, several well-marked cases occurred, in low, ill-ventilated lakes, among densely crowded inhabitants "living in poverty, wretchedness, and filth."

"In some houses, several families were living in actual contact with a mass of putrefying animal and vegetable matter."

"Upon every inquiry, there are no grounds for supposing, that cholera was imported into Cork. A strict quarantine was established upon all vessels arriving in the harbour from suspected ports, and not a single case occurred amongst the shipping. Although the epidemic existed for some weeks in Dublin, before it appeared in Cork, still the persons first attacked in the latter city, could not possibly have had any communication to give reason for suspecting that the disease was produced by infection. It is a singular fact in the propagation of this epidemic, that it should have broken out and spread along the eastern coast of England, whilst so many great towns in the same parallel, upon the western part of the Continent escaped, and that it should have established itself in so extensive and severe a form in Dublin and Cork, upon the eastern and south-eastern coast of Ireland, before any town upon the western coast of England had been attacked."

Dr. Bullen considers the epidemic as a febrile disease presenting three stages, now so well recognized. In the first stage he recommends emetics, followed by calomel and other aperients. In the second stage, or that of congestion, he depends chiefly on calomel. He assures us that "not a single case of cholera has been lost in the North Cholera Hospital, where the constitutional effects of mercury were produced." He placed a scruple of calomel on the tongue, with one grain of opium, and washed it down with some warm brandy and water. If relief was not produced, he repeated the dose every two hours, until three doses were exhibited. In the stage of consecutive fever, the treatment depended on the symptoms, and on the organs chiefly affected. Dr. B. properly deprecates specifics in a disease consisting of distinct periods, each requiring appropriate treatment. The pamphlet is creditable to the author.

---

### XI. Saline Injections in Cholera.

When the administration of saline medicines, whether by the mouth or per anum, was proposed for the cure of cholera, we then observed that this practice was surely carrying coals to Newcastle, since the saline ingredients of the blood were drained from the circulation into the alimentary canal, and consequently the addition of other saline medicines in that quarter was superfluous. We find that Dr. Latta of Leith, in his communication to the Central Board of Health, avers that the introduction of saline solutions into the intestines of cholera patients "aggravated the tormina, vomiting, and purging." So much for Dr. Steeven's plan of curing cholera, said to be successful in the hands of one practitioner in London. Of that success we shall here say no more than that any remedy, however inert, or even absurd, may gain great reputation towards the close of an epidemic, when Nature herself is generally equal to the cure. But the physical effects, resulting from the injection of aqueous saline fluids into the blood, are of a positive nature, and capable of demonstration. They appear, in the present case, to be very important, and likely to lead to other and still more important results. The following extract from Dr. Latta's letter, as published in the Lancet, is deserving of great attention.

"The first subject of experiment was an aged female, on whom all the usual remedies had been fully tried, without producing one good symptom; the disease, uninterrupted, holding steadily on its course. She had apparently reached the last moments of her earthly existence, and now nothing could injure her—indeed, so entirely was she reduced, that I feared I should be unable to get my apparatus ready ere she expired. Having inserted a tube into the basilic vein, cautiously—anxiously, I watched the effects; ounce after ounce was injected, but no visible change was produced. Still persevering, I thought she began to breathe less laboriously, soon the sharpened features, and sunken eye, and fallen jaw, pale and cold, bearing the manifest impress of death's signet, began to glow with returning animation; the pulse, which had long ceased, returned to the wrist; at first small and quick, by degrees it became more and more distinct, fuller, slower, and firmer, and in the short space of half an hour, when six pints had been injected, she expressed in a firm voice that she was free from all uneasiness, actually became jocular, and fancied all she needed was a little sleep; her extremities were warm, and every feature bore the aspect of comfort and health. This being my first case, I fancied my patient secure, and from my great need of a little repose, left her in charge of the hospital-surgeon; but I had not been long gone, ere the vomiting and purging recurring, soon reduced her to her former state of debility. I was not apprised of the event, and she sunk in five and a half hours after I left her. As she had previously been of a sound constitution, I have no doubt the case would have issued in complete reaction, had the remedy, which already had produced such effect, been repeated.

Not having by me the number of The Lancet containing Dr. O'Shaughnessy's analysis, I adopted that of Dr. Marcet, only allowing a smaller proportion of saline ingredients. This I now find to be considerably less than natural, according to the more recent analyses. I dissolved from two to three drachms of muriate of soda and two scruples of the subcarbonate of soda in six pints of water, and injected it at temperature 112° Fah. If the temperature is so low as a hundred, it produces an extreme sense of cold, with rigors; and if it reaches 115°, it suddenly excites the heart, the countenance becomes flushed, and the patient complains of great weakness. At first there is but little felt by the patient, and symptoms continue unaltered, until the blood, mingled with the injected liquid, becomes warm and fluid; the improvement in the pulse and countenance is almost simultaneous, the cadaverous expression gradually gives place to appearances of returning animation, the horrid oppression at the præcordia goes off, the sunken turned-up eye, half covered by the palpebræ, becomes gradually fuller, till it sparkles with the brilliancy of health, the livid hue disappears, the warmth of the body returns, and it regains its natural colour,—words are no more uttered in whispers, the voice first acquires its true cholera tone, and ultimately its wonted energy, and the poor patient, who but a few minutes before was oppressed with sickness, vomiting, and burning thirst, is suddenly relieved from every distressing symptom; blood now drawn exhibits on exposure to air its natural florid hue.

Such symptoms, so gratifying both to the sick and the physician, must never allow the latter to relax in his care—the utmost vigilance is still necessary. At first the change is so great that he may fancy all is accomplished, and leave his post for a while. The diarrhœa recurring, he may find his patient, after the lapse of two or three hours, as low as ever. As soon as reaction by the first injection is produced, mild warm stimulants, such as weak gin-toddy, mixed with some astringent, should be freely and assiduously administered. An attempt should be made to fill the colon with some astringent fluid. That such is requisite, is evident from the watery diarrhœa returning with violence, and if not restrained, death will ultimately make sure of his victim, therefore so soon as the pulse fails, and the features again shrink, the venous injection must be repeated, taking care that the fluid in use retains its proper temperature. The injection should be carried on very slowly, unless the patient is much exhausted, when it may be used more rapidly at first, until a little excitement is produced, after which it should not exceed two or three ounces per minute, and now is the time for the exhibition of astringents by the mouth, which will be retained, for in general the sickness entirely leaves during the operation."

Other cases, tending to shew similar effects, though not all successful, are related by Dr. Lewins, Dr. Craigie, and Dr. Mackintosh. We hope that future experience may confirm the sanguine anticipations which the above cases are calculated to excite.

---

### LXXVIII.

### DR. FALRICH'S WAX MODELS.

THE models that we have examined are faithful reprentations of the natural structures, and are very creditable specimens of this beautiful art.

No. 1. This model is principally confined to the anatomy of the superficial parts of the cranium and face, a few structures only being shown in the neck. The facial muscles are very accurately represented, but the principal merit of this piece is the beautiful view it presents of the disposition and connexion of the several facial nerves. The branches of the portio dura (respiratory nerve of the face of Bell's classification) the three terminations of the 5th pair (the supra orbitar, infra orbitar, and inferior dental) together with the intimate communications of these nerves, are finely and accurately displayed. This piece also shews the inosculations aud courses of the several blood-vessels, especially the veins of the scalp and face. The opposite side of the model (one side only being dissected) contains a correct representation of the features and external configuration of the cranium and face.

2. This model shews all those parts which are exposed by making a vertical and median section of the skull and face and upper part of neck. It is a very useful model, because it accurately represents the *relative situation* of the numerous and important organs contained in the cranium and face. Thus it displays the several coverings of the skull—the two laminæ of the bones—the diploe—the position and relative connexion of all those parts of the cerebrum, cerebellum, and medulla oblongata, that are placed on the median plane. In the face it shews the septum narium, the cavity of the mouth, the palate, uvula, and the parts connected with the fauces, pharynx and larynx.

3. Contains the stomach, liver, gall-bladder and kidneys. The stomach is faithfully and correctly represented as to its form, colour, vessels and nerves. The concave surface of the liver, with its lobes, fissures, nerves, and gall-bladder, is also shown. I consider this model and No. 1, as the best in the collection.

4. Bend of the arm, showing the parts connected with venesection. The superficial veins and their relation to the brachial artery are well displayed. The median nerve is introduced, but the cutaneous ramifications are omitted, which in such a model is a defect.

5. This is a very good representation of the several parts of the base of the brain, including the blood-vessels, and the origin of the several cranial nerves. This is a useful model. There are some other good representations of the convolutions and of the brain.

6. A very natural model of the position of the fœtus in utero at the full term—showing the position of the head, trunk and limbs—likewise the umbilical cord, and a part of the placenta, with the connexion of the funis.*

---

### LXXIX.

A STATISTICAL VIEW OF THE NUMBER OF PERSONS REPORTED TO HAVE DIED OF EACH OF MORE THAN 100 KINDS OF DISEASE, AND CASUALTIES WITHIN THE BILLS OF MORTALIY, IN EACH OF THE TWO HUNDRED AND FOUR YEARS, 1629—1831, &c. &c. By J. MARSHALL, ESQ. Quarto, pp. 81, with numerous Tables, &c. 1832.

IT is the melancholy but the useful task of the medical philosopher to desist occasionally from the *treatment* of diseases, in order to mark their rise, acmé, and decline on the great chart of human existence—to calculate arithmetically the *rate* at which they abridge the span

* The above models we have seen at Mr. Highley's in Fleet Street.—*Ed.*

of life, and mark their influence on the population of states and cities. This is a study which expands our ideas, enlarges our views, and philosophises the mind. Our readers will therefore excuse a short article in this place on a subject which has too seldom occupied the pages of our Journal.

The volume before us is a very curious, elaborate, and meritorious work, which ought to experience the patronage of the profession—especially of the higher grades, the corporate bodies, the libraries, and the book-societies. The labour of examining the bills of mortality for upwards of two hundred years, must have been prodigious, and we sincerely hope that Mr. Marshall will be substantially rewarded for his praiseworthy pains and research. The approach of the far-famed epidemic cholera no doubt stimulated the author in his undertaking; but it appears that Mr. Marshall was at work on the subject long before the pestilence broke out in this country.

It was in 1629, that reports of diseases and casualties, of christenings and burials, within the bills of mortality, first began to be published by the worshipful company of parish clerks; and it has been objected to these returns that the office of reporting deaths and casualties has devolved on old women entitled "SEARCHERS," when it ought to have been filled up by medical men. There is no doubt that the reports would have been more exact in a technical point of view, had medical practitioners been employed; yet still, as the searchers could have no rational motive for misrepresentation, we may conclude that the returns are, upon the whole, as accurate as could be expected even under better auspices. The most interesting features of these reports are seen in the uniformity of some diseases, the extinction of others, and the seeming origin of new ones—and last, not least, the manifest aggregate diminution in relation to the aggregate population. Unfortunately we have no accurate census of the population prior to the year 1750. And even since then, we have some hiatuses and defects. It appears, however, that the population of London rather decreased than augmented from 1700 to 1750, more especially within the walls of the city. In 1631, the population of the metropolis was 130,178. In 1700, it amounted to 208,300.

"From the preceding data, and statement of Burials, as exhibited at page 67, it appears that the proportion of deaths to the Total Population of the City, in 1631, was as 1 to 21; and, in 1700, as 1 to 24; while the proportion of Burials in the whole of the Metropolis, according to the Statements at pages 62, 63, and the Statement of the Population at page 12, was as 1 to 33 of the Total Population in 1700; 1 to 26 in 1750; and as 1 to 52 in 1820. It must be observed, however, in regard to the otherwise seeming disparity between the proportions of Burials in 1750 and 1820, that the two Metropolitan Counties of Middlesex and Surrey, in the twenty years 1801—1820, received an accession of more than 375,000 Inhabitants, as immigrants from other parts of the United Kingdom, &c.; and this, it will be seen on reflection, without, comparatively speaking, any material increase of Deaths. From this also it may be inferred not only how difficult it is, but how entirely futile it must be, to attempt to establish, with arithmetical precision, an exact scale of proportion which the deaths bear to the living, not only in the Metropolis, but in any particular district where the facilities of intercourse and tendency to change of location so extensively prevail."

It is not a little curious to observe the difference of mortality in child-bed, between the early and the latter parts of the two hundred years embraced by these tables. In the *former*, the annual average of deaths was 234—in the latter 201, while the number of births had been nearly doubled. Abortions and still-born are more uniform.

"The great number reported from 1647 to 1752 to have died of *Teething*, and the progressive diminution since the latter date, form a feature deserving attention, as does also the gradual to total extinction of *Rickets,* after much prevalence from 1647 to 1715; and generally the diminution of diseases *incident to infancy* may be regarded a circumstance as honorable to the assiduity and integrity of the Medical Practitioner, as it is gratifying to the Friends of social Improvement. There are, however, some indications of Nature seeming determined to cross the purposes of the Medical Practitioner, in despite of all his assiduity: *Coughs, Croup, and Dropsy in the Brain,* all indicate a determination, since 1790, to fill up the void of Mortality which the diminution under the head of *Convulsions* otherwise seems disposed to occasion. *Dropsy in the Brain,* in particular (see Col. 10, Period IV), appears to deserve attention: how far this Disease may have been confounded with Convulsions, or otherways, prior to 1790, is a point which I must leave to the Medical Professor to determine. In the three preceding periods it is reported under *Headmoldshot, Horse Shoe Head,* and *Water in the Head;*—and its prevalence, as reported from 1715 to 1752, and gradual diminution, almost to extinction, subsequent to the latter date, form another feature deserving of notice."

Consumption, which forms so prominent a feature in the catalogue of diseases, may be regarded as remarkable for its uniformity, especially in relation to asthma and "phthisic," with which it is blended from 1690 to 1700, and from 1729 to 1739. Prior to 1690, asthma is not specified. In the three latter periods (embraced by these reports) consumption, asthma, and phthisic, are placed in juxta-position. From 1629 to 1710, the eruptive fevers, small-pox and measles, are as remarkable for their variableness as some other diseases are for their uniformity.

"Subsequent to the introduction of inoculation, the Small-Pox became far more general and confirmed in its virulence, which continued up to the period of the introduction of Vaccination, since which time it has gradually abated; but it is a fact, which seems in a very high degree to deserve attention, that the *Measles,* which continued exceedingly variable and moderate down to 1800, since that date (notwithstanding, as is fair to be presumed, the improved treatment of the Disease) have become greatly increased and confirmed in virulence. The increase of *Erysipelas* also, as one of the order of Eruptive Fevers, although at present inconsiderable in its effects, is not undeserving of notice."

From 1629 to 1689, fevers seem to have been exceedingly variable; but, from the latter date, down to 1750, more uniform and general. From 1750 to 1831, fevers have gradually diminished, while inflammatory complaints have increased in a still greater ratio than the pyrexiæ have receded. Dropsy appears to have progressively increased from 1629 to 1750, subsequent to which date, it seems to have abated, but manifests a tendency to increase again, since 1810.

Of the pestilential epidemics, the great one of 1348-9, was the most terrific. It commenced in Cathay (Western Tartary) in 1345, whence it spread North, West, and West and South, to every part of the habitable world. The consternation which it produced, and its various modes of action are well described by Cantacuzenus, a Greek historian of that period, of which a translation was given by Mr. Barnes, of Cambridge, in 1688. The following extract is an interesting curiosity.

"This plague, taking its rise from the Scythians or Hyberboreans, overran almost all the sea coasts of the habitable world, and destroyed an incredible number of people. For it did not only pass through *Pontus,* and *Thrace,* and *Macedonia,* but also through *Hellas,* and Italy, and all the Isles; and Egypt, and Lybia, and Indæa [Ethiopia], and Syria, and in a manner all the world round about. But it was such an unconquerable evil, that neither any diet nor strength of body could resist it; for it pulled down all bodies alike, as well the strong as the weak, and those who were most diligently looked after perished as well as those who wanted all things. That year was free from all other distempers; but, if perhaps any person was sick before, all other distempers terminated in this. Here the knowledge of the physicians was put to a stand: for some, enduring a little, died the same day, some the same hour; but those that held out the second or third day were first taken with an acute fever, and, the distemper getting up into the head, were rendered speechless, and insensible to all that was done; and so dropt off as it were in a profound sleep. But, if any ever came to themselves a little, they endeavoured to speak something, but the tongue was with difficulty moved; and so uttering many inarticulate things, the nerves being stupified in the hinder part of the head, they presently died. Others were not taken in the head but in the lungs: these had an inflammation in their inwards, which created acute pains about the stomach, so that they sent up blood and a loathsome and cadaverous stink from within; their jaws and tongues were dried up with heat, and black and tainted with gore; and whether they drank much, or little, it was all alike: these could take no sleep, but were in continual pain and disquiet. Some had imposthumous ulcers, and black blisters, bigger or lesser on their arms and under their arm-pits; and some in the cheeks, and others in other parts of the body. And in others there arose black nodes, spots, or tokens, over all the body, in some more superficial and visible, in others deeper and obscure: and yet of both sorts all died alike; for some had all these symptoms together, others more or less, but to most even one of all was enough to do the business. Yet those few who recovered were no more touched with the same mischief, but remained secure; for it never took any twice so as to kill. Sundry times there were great imposthumes in the thigh, and in the arms, which being cut sent forth much stinking matter; and so the disease was carried off, flinging forth together therewith all the noxious humours. And yet some, though they had all these symptoms, were beyond all expectation saved. But there was no certain remedy: for what was good for one was to another in the same condition fatal: yet he that cured another got his own death thereby, and this made the greatest havoc, so that houses were emptied of inhabitants, even brute creatures, dying with their masters. Yet nothing herein seemed more dismal than the despair to which men were reduced: for when any one perceived himself sick he abandoned all hopes of recovery; and thus they gave themselves over and died presently, adding their defection of mind as an assistance to the disease. Such a kind of malady cannot be expressed; and it was most manifest that it was not any plague natural or common to mankind, but a scourge from heaven: wherefore many also were bettered thereby, not only of those who died, but as many as survived. For then, casting aside all their vices, they applied themselves wholly to the study of virtue, and several gave all they had to the poor. But, when any found themselves affected, there was none so stony or so obdurate but that he repented heartily of his sins, and thereby gave the Divine goodness some occasion of being gracious at his tribunal."

Naples lost 300,000 people—Florence 400,000, and Avignon was depopulated. Nearly four million of souls perished within the pale of the Holy See! Paris lost 50 thousand, and Norwich 57 thousand—the same number that died in London. The following passage is curious at this time.

"One of the worst features to which the Pestilence of 1345—50 gave rise, was the accusation of different Sects and Parties of Poisoning the Waters, and other similar Mal-practices, and assigning to them the cause of the desolation which had prevailed. Both Christians and Jews, in their turn, appear to have been persecuted unto death in great numbers. At Mentz 12,000 Jews are stated to have fallen victims to the fury of an ignorant and brutal populace, under the pretext of their having poisoned the waters of the wells of that city."

In 1379, we find the mortality from an epidemic was so great that "the country became almost desolate." In 1478, the sweating sickness—in 1500, 30,000 died of the plague in London—in 1518, the same disease carried off infinite numbers of nobility and oth ers. In 1529, the sweating sicknessp rev iled all over the kingdom—1542, hot agues and fluxes—1545, the great plague—1552, the sweating sickness again, and very general—1557, hot burning agues, seven aldermen in London died—1563, 21,500 died of the plague in London—1593, the plague again, of which 17,890 died in London—1603, the plague carried off 30,000 in London. The bills of mortality, or "display of the diseases and mortality," as they are called, commences from the latter date. When we examine the table (page 66) shewing the proportion of deaths from plague, in each week, from 1593 to 1665, we find that it prevailed 20 years out of that time, during some of which, viz. in 1603 and 1625, it nearly equalled in mortality the great plague of 1665. Thus, in the months of July, August, and September, 1625, it killed from one to four thousand weekly in London. In 1665, it presented itself in the last week of April—rose gradually to seven thousand a week, and fell in December to 281. In that fatal Summer, it carried off 68,506 souls!

When we contemplate the bills of mortality in this book, and compare the devastations of former epidemics with those of our own times, we have reason to be thankful for the improved state of health enjoyed by the inhabitants of this great metropolis.

We recommend this volume to the patronage of the profession—a patronage which, we are sorry to say, has been denied it, in one, at least, of those great corporate bodies where patronage ought to be found!

---

### LXXX.

### CYCLOPÆDIA OF PRACTICAL MEDICINE, Parts III., IV., and V.

(Continued from last Number.)

THIS work goes on prosperously—indeed its success is now beyond doubt. We shall not attempt to notice all the articles in this vast assemblage, especially in an analytical manner. Nevertheless, we shall glance at those which are most adapted for comment or quotation.

1. AUSCULTATION. This, of course, is from the pen of Dr. Forbes, and we need hardly say that it is well executed, seeing the great pains taken by the author in translating Laennec, and in prosecuting the subject of auscultation in his own practice. We can only give the following extract from this condensed article.

AUSCULTATION OF THE HEART IN THE FŒTUS—DIAGNOSIS OF PREGNANCY.

"M. Mayer, of Geneva, appears to have been the first who applied the tests afforded by auscultation to discover the existence of the fœtus in utero. In the *Bibliothéque Universelle* for November, 1818, it was stated that this gentleman had ascertained that the sounds of the fœtal heart are readily perceptible by applying the ear to the abdomen of the mother. The subject was afterwards prosecuted, with the aid of the stethoscope, by M. Kergaradec, and with additional results indicative of its importance; and the following facts, verified by numerous auscultators in this country and on the Continent, are now as well ascertained as any detailed in former parts of this article.

There are two auscultatory signs of the presence of a living fœtus in the womb—1. the double sound of the heart; and, 2. the existence of the bellows-sound in the placental arteries, or rather, in the uterine arteries immediately connected with the placenta.

1. The pulsations of the fœtal heart may be perceived as early as the fifth month, or between that and the sixth. They are characterised by the double sound of the adult heart, and by the peculiar rhythm of this, which can never be mistaken for any other sound. The sounds, when compared with those of the adult heart, examined in the cardiac region, are extremely feeble; but they are, when at all perceptible, perfectly distinct. They are distinguished from all arterial pulsation of the mother by the duplex rhythm, and by the extreme frequency of their recurrence. The fœtal pulsations are generally more than double that of the mother. The sounds are found to be much more distinct at one time than another, in the same place, and in different places at different times. These variations no doubt result from the varying position of the fœtus, and from its relation to the uterine parietes.

The sounds will be much more distinct when the trunk of the fœtus is in direct contact with the uterus, and, most of all, when the anterior part of its chest is applied opposite to the spot on which the stethoscope rests. When the whole body of the child is removed from that side of the uterus over which the instrument rests, it is doubtful if the action of the heart can be at all perceived. The more copious is the liquor amnii, the less perceptible, *cæteris paribus*, will be the fœtal sounds. The space over which the sounds may be heard is generally pretty extensive, being, indeed, frequently the greater portion of the region occupied by the uterus. It is, however, in general easy to discover the point which is nearest to the source of the sounds, by the decreasing and increasing intensity of these, as our examinations recede from or approach towards one particular spot. In general, the pulsations of the fœtal heart vary from 120 to 160 in a minute. In one case noticed by Dr. Ferguson,* the relative frequency of the mother's and child's pulse was singularly reversed; the former being 100, while the latter was only 20. We have met with no similar instance on record.

2. The second auscultatory sign of the presence of the fœtus has been termed the *placental sound*, or *placental bellows-sound*, likewise the *utero-placental soufflet*. It was first noticed by M. Kergaradec, and although a less absolutely certain indication of pregnancy than the sound of the fœtal heart, it is a sign of great importance. Although termed placental, there can be little doubt that the site of the sound is in the uterus, in the enlarged arteries which supply the placenta.† The sound assumes, at different times, all the character of the bellows-sound of the heart. It becomes perceptible about the fourth month of gestation, and is then more distinct than at a later period. Dr. Ferguson says that he has not observed any variety of the sounds to be peculiar to particular stages of pregnancy. In the early stages, Laennec describes the sound as resembling that which is produced by discharging a blast from a pair of bellows into an empty bottle. At a later period, the sound is duller, more diffused, and no longer conveying the impression of being limited to the caliber of a single artery. It assumes all the varieties of the valvular sounds of the heart, from the simple bellows-sound to the sound of the rasp or saw. Dr. Ferguson says that the most constant variety 'is a combination of the bellows or the sawing with the hissing sound, commencing with one of the former and terminating with the latter.' These sounds are confined to a much smaller space than those of the fœtal heart. The point where they are most audible is always fixed in the same person, but varies in each individual. In some, the sound can only be detected in a single spot a few inches square. There is no particular part of the uterine tumour in which the sound may not be found; but Dr. Ferguson says that he has found it most frequently in the iliac regions. The placental sound is always isochronous with the pulse of the mother, and is unaccompanied by any degree of impulse.

Whatever be the precise mode of production of this sound, there can be no doubt that it has its seat in the enlarged vessels of the uterus in that portion of it immediately connected with the placenta. This is proved by the following facts:—1. The sound, as already stated, is confined to a fixed space in each individual. 2. This spot is ascertained, by examination after delivery, to be always that to which the placenta had been attached. 3. That the sound is not seated, at least exclusively, in

* Dublin Trans. vol. I. New Series.

† "A striking analogical instance is afforded by the thyroid arteries in bronchocele, noticed in a former part of this article. *Query*, will the same result be obtained in active hypertrophies of other parts of the system?"

the placenta, is proved by the fact that the sound is still audible for a short period after the placenta is detached. 4. It ceases immediately upon the contraction of the utero-placental arteries, as is proved in cases of death of the fœtus without delivery, and by its instantaneous cessation on the contraction of the uterus after delivery. 5. It is in all cases synchronous with the mother's pulse. There seems little ground for believing, with Dr. Kennedy,* that the placental arteries themselves have a share in the production of the sound, any farther than by their action promoting that of the uterine arteries. The character of the sound is *changed* by the cessation of the fœtal circulation, as by the removal of the placenta, the death of the fœtus, or tying of the cord (Dr. Kennedy denies M. Ollivry's statement that it is immediately *extinguished)*, the sound becoming 'abrupt, of short continuance, and wanting the lengthened terminating whiz observed in the perfect sound.' This may easily be explained by the change necessarily produced in the uterine circulation by the removal of the fœtal circulation, without supposing that the placental arteries had any direct share in its production.

Great care and attention are requisite on the part of the auscultator in investigating these phenomena. The sounds being very feeble, perfect silence is necessary during our explorations, and we must be very careful not to confound the sounds with others which may exist at the same time, such as the sound of the mother's heart (which is often distinctly audible in the region of the uterus), of the intestinal movements, and the sound of muscular contraction produced in compressing the abdomen with the stethoscope. As the phenomena are sometimes intermittent, or, at least, not always perceptible, we must not deny their existence because they are not discovered on the first examination.

The sounds may be heard almost as well by the naked ear as the stethoscope; but to all who are accustomed to use this instrument, there are numerous and very obvious reasons why it should be preferred. It is, however, well that practitioners should be aware that the phenomena may be detected by any one, whether a practised auscultator or not."

The article bathing is also from the pen of Dr. Forbes, and occupies 48 columns. Dr. F. collects all the best information from the best sources, and concentrates them for the benefit of his readers in a moderate compass.

II. BLOOD AND BLOOD-LETTING. By Dr. MARSHALL HALL.

As the talented author has paid great attention to these subjects (especially the latter) and written, *ex professo*, on one of them, we may consider this article as a resumé of the writer's more extended work. He divides his remarks into two sections—the first treating of the natural, the second of the morbid state of the vital fluid. On the natural condition of the blood we shall not dwell. The morbid changes relate to quantity as well as quality. The blood is often deficient or superabundant—as in anemia and plethora. In various diseases, Dr. M. contends that the qualities of the blood are deteriorated, and seems inclined to infer, that these deteriorations are the cause, rather than the effect of disease. Much may be said on both sides of this question.

"Considerable changes are induced in the blood in *inflammation*. It is well known that the blood drawn in inflammation of the serous membranes is apt to be cupped and buffed; that is, the cruor is firm and contracted, and its edges are much raised above the central part of its surface; and its upper portion consists in a substance nearly deprived of colouring matter. This buff consists of fibrine and albumen; the serum contains more albumen than natural; and the crassamentum is exceedingly firm.

Is this modification of the blood the effect, or a cause, of the inflammatory process? That the blood may be cupped and buffed independently of inflammation, is proved by the facts of the occurrence of these phenomena in cases of pregnancy.

* Dublin Trans.

It is true, on the other hand, that the buffed and cupped state of the blood is not so obvious on the first day as on subsequent days, in cases of inflammatory disease. It must, therefore, be concluded that inflammation, and the state of pregnancy, and other sources of excitement, and even bloodletting itself, when so conducted as to lead to reaction, induce the cupped and buffy state of the blood.

It is an interesting fact to note, that the layers and adhesions formed upon or from the surfaces of inflamed serous membranes, are of the same chemical and physical constitution as that of the buff of the blood itself.

It is important to remark that it is not every kind of inflammation which induces the cupped form and buffy coat in the blood. Inflammation of the serous membranes and parenchymatous substance has chiefly this effect. Inflammation of the mucous membranes, as bronchitis, and dysenteria, frequently occurs without any disposition to a cupped or buffed condition of the blood. Indeed, there is a greater difference between these two classes of disease than their names would lead us to suspect.

Dr. Tweedie has observed, in two instances of acute disease, the buffy coat on arterial blood. The only reason why this appearance is not so familiar to us is, probably, the less frequent performance of arteriotomy than of venesection.

But besides the marked morbid appearances of the blood in fever and in inflammation, very peculiar changes take place in *chlorosis.* In this disease there is a general state of bloodlessness, the crassamentum is small, the proportionate quantity of serum large; a drop of the blood observed upon white linen is seen to be extremely pale. It still remains to ascertain the exact proportions of fibrine, albumen, hæmatosine, &c. in chlorotic blood, and to observe its appearance under the microscope. It still remains to compare it with the blood of persons blanched by hemorrhagy. The morbid character of the blood in chlorosis must be ranked amongst the most unequivocal proofs of the truth of a humoral pathology.

The next disease to be noticed in connexion with the pathology of the blood is *scorbutus,* the stronghold of the humoral pathologists,—the pons asinorum of the solidists. The defective cohesion and separation of the cruor, the dissolved state of the blood in scorbutus, could never be forgotten by either party; it was continually reproduced by the former, and pertinaciously neglected by the latter.

Dr. Mead gives a vivid description of the state of the blood in scorbutus. 'In the beginning,' he observes (Medical Works, Dublin, 1767, p. 332), 'as it flowed out of the orifice of the wound, it might be seen to run in different shades of light and dark streaks. When the malady was increased, it ran thin, and seemingly very black; and after standing some time in the porringer, turned thick, of a dark muddy colour, the surface in many places of a greenish hue, without any regular separation of its parts. In the third degree of the disease it came out as black as ink; and though kept stirring in the vessel many hours, its fibrous parts had only the appearance of wool or hair floating in a muddy substance.

'In dissected bodies the blood in the veins was so entirely broken, that by cutting any considerable branch you might empty the part to which it belonged of its black and yellow liquor. When found extravasated, it was of the same kind. And lastly, as all other kinds of hemorrhage are frequent at the latter end of the calamity, the fluid had the same appearance as to colour and consistence, whether it was discharged from the mouth, nose, stomach, intestines, or any other part.'

There is no point in pathology more instructive than this of scorbutus. The morbid influence of salt meat, and the curative influence of acids; the baneful effects of impure air and impure diet, and the beneficial effect of a change of atmosphere and regimen; are shewn more clearly in this disease than in any other. And as subsequent links of the chain, the influence of a

'dissolved' state of the blood in inducing a 'dissolved' state of the solids, to use the ancient phraseology, is equally remarkable in this disease.

Nearly allied to scorbutus is *purpura*. Some singular appearances have been observed in the blood in this disease. The crassamentum has been found separated without being firm, or contracted; and buffed without being cupped; its upper surface covered to a considerable depth by a straw-coloured substance resembling ordinary jelly. This, as well as the other parts of this interesting subject, still needs further investigation.

As an improper kind of diet produces a baneful influence upon the composition of the blood, so does a disordered and *loaded state of the bowels*. This is apparent from the condition of the secretions; the saliva, the perspiration, the urine, are alike deranged in their obvious qualities, but especially in odour; and there is more or less of the bloodless appearance seen in chlorosis.

*Icterus* is another disease in which the blood is known to suffer. The colouring principle, at least, of the bile is retained in the blood. It appears to act as a narcotic upon the brain, inducing drowsiness. The serum of the blood and the secretions from the skin and kidney are in some cases alike tinged with the yellow colouring matter of the bile.

Every one knows the baneful influence of a suppressed state of another secretion—*the urine*. It cannot be doubted that some of the principles of this secretion are retained in the blood. Prévost and Dumas found urea in the blood of an animal in which the ureters had been tied. In the human subject, suppression of the urine, or the presence of some of its principles in the blood, produces coma and death.

M. Dance has recently renewed the idea of a former day, that the suppression of the secretion of milk may, like that of the bile or urine, affect the blood and the secretions. In one such case it is said that caseum was found in the fluid of ascites, evacuated from the abdomen.

But if the secretions become sometimes suppressed, and produce a reflex alteration in the blood, there is a case in which the blood becomes morbidly affected, together with an excessive secretion, as in diabetes. The blood in this disease is more serous, and contains less fibrine than usual. It would be interesting to examine the effect upon the blood of excessive perspiration, undue lactation, and profuse or protracted menorrhagia or leucorrhœa.

The facts which result from this inquiry are these:—1. In many diseases, as fever, inflammation, chlorosis, scorbutus, there are undoubted morbid changes induced in the blood itself; 2. that other morbid changes are induced by impure atmosphere and an unwholesome diet; 3. that various miasmata in the atmosphere, and various poisonous qualities in food, produce morbid changes in the blood; 4. that suppressed secretions induce morbid changes in the blood; 5. that excessive secretions induce other morbid changes in this fluid; and, 6. that an affection of the innervation, or of the nervous system, may, as is observed on a division of the eighth pair of nerves, produce its peculiar changes in the circulating mass."

On bloodletting Dr. Hall is more at home; and his paper in the Cyclopædia is a compendium of his other publications on the same subject. We need not farther allude to them here.

The important article on Cerebral Inflammation, by Dr. Adair Crawford, is noticed separately in another part of the Periscope.

III. BRONCHITIS, by Dr. Williams, is a well-constructed paper, from which we shall make an extract on the treatment of the acute form of that dangerous and distressing disease.

*Treatment of Acute Bronchitis.*—1. The slighter cases of acute bronchitis are more frequently objects of domestic than of formal medical treatment. When neglected, however, a common cold may give rise to the most serious complaints; and we are far from subscribing to a prevalent opinion to which even Laennec seems to lean, that it must always run its course. By antiphlogistic diet, and a few simple means calculated to restore the secretions, and, if required, to cause revulsion from the inflamed part, a cold may often be checked in a few days, which if left to itself might run on for weeks, entailing on some subjects other and worse evils. The means which we have found most effectual are as follows:—At the first feeling of the cold, let a purgative be given, with two or three grains of ipecacuanha or James's powder. Let a hot pediluvium be used, the patient getting into a warm and well covered bed immediately after, and promoting any disposition to perspiration by a warm draught of thin gruel, barley water, or any other mild diluent. If perspiration comes on, and the purgative operates well, the cold is sometimes already cured; and it is only necessary to remain at home, and to abstain from animal food and wine next day to prevent a return.

If, however, perspiration do not follow, or if the cough has already come on, the disease generally proceeds, and we must then endeavour to mitigate its severity, and bring it quickly through its course. If the patient cannot, or thinks it not worth while to remain in bed for a day or two, he should clothe more warmly than usual; for the susceptibility of the body is such as to render a very slight exposure enough to keep up the disease, and abstinence from meat and all fermented liquors or spirits is at this period equally necessary. To loosen the cough and lead the bronchial inflammation to the stage of free secretion, small doses of ipecacuanha or tartarized antimony are often most effectual; and, although called a stimulant expectorant, squills, when combined with these, have always appeared to us to exert a beneficial effect: ten minims of the tincture with thirty of the vinum ipecacuanhæ, and six or eight of liquor potassæ, given three or four times a day, seldom fail to facilitate expectoration and relieve the cough. Whether the alkali act by facilitating the absorption of the medicine, or by any specific action on the vessels of the bronchial membrane, we cannot determine; but its effect in increasing the action of expectorants we have proved in a variety of examples. Should nausea be produced, the dose of the medicines may be diminished; and if the cough is still troublesome at night, a little extract or tincture of hyosciamus may with advantage be added.

This treatment is generally sufficient to relieve the tightness across the chest, which yields as the cough becomes loose; but if the case is obstinate, it may be necessary to resort to a blister, or some of the means which we shall presently recommend for the more severe forms of the disease.

The inhalation of the vapour of warm water may be sometimes useful; but we have seen it in some instances increase the oppression; and, from its effect of determining blood to the part, we do not deem it so universally safe a measure in recent bronchitis as it has been represented to be.

With still more force would we object to the cure by spirits or wine, as recommended by Laennec; not that we doubt its occasional efficacy, but from the experience that where it does not cure, it greatly aggravates the inflammation. Towards the termination, when the matter expectorated has become thick and loose, and all febrile symptoms are dissipated, animal food and wine may be indulged in with impunity, and even with advantage. At this period, too, if the disease shows a disposition to linger, much good may often be obtained by using some of the stimulant expectorants, as myrrh, copaiba, &c. and if necessary, the cough may be allayed by opium. This treatment will be noticed under the head of chronic bronchitis.

There are very many remedies which are in common use for coughs, which it is impossible here to advert to. The safest are those which are simply demulcent; many are stimulant and narcotic, and often do mischief. Ipecacuanha lozenges is a remedy worthy to be named, as the convenience of its form recommends it when more formal medicines are objected to; and although it is apt to cloy the stomach before sufficient quantity can be taken, it often proves as serviceable as the less agreeable compounds of the medicine.

2. The danger attending severe acute bronchitis renders far more energetic measures necessary; and these, although generally of the same class as those usually directed against acute inflammations, must be exercised with more than usual discretion and caution.

As long as the phlogistic state continues, with high fever, hard pulse, and great feeling of straitness and oppression in the chest, there can be no doubt of the propriety of bleeding more or less freely, according to the intensity of the symptoms, and the strength of the patient; but it is not prudent, as in pleurisy or peripneumony, to bleed to syncope, or to push the measure with an expectation of very marked relief. From sixteen to twenty-four ounces may be taken at first in severe cases; and the repetition of general bloodletting must depend on the state of the pulse rather than on other symptoms. If it is weak, or if the patient is advanced in life, we must endeavour to subdue the symptoms by less violent means, such as local bleeding and medicines, rather than run the risk of destroying strength that is absolutely required for the act of expectoration.

Cupping is generally to be preferred to leeches, as its effect is more speedy and more within control; and it is of considerable advantage to apply it to the part in which auscultation has discovered the greatest obstruction to the passage of the air.

There is an objection which obtains so against blisters, as to exclude their use in many cases of the early stage of acute bronchitis; namely, that before they rise, they always produce an irritating effect on the whole system, which is very apt to aggravate for the time the inflammation for which they are applied. This effect, however, varies much in different individuals, and in those whose skin easily vesicates under a blister, it is obviated by the serous discharge that immediately succeeds.

In other instances, it is advisable to resort to other counter-irritants, and we give the preference to the tartar emetic. To make it available in an acute disease, we have found it necessary to excite the skin previously to its application, by means of a brush or coarse flannel, by applying a warm hand wetted with ether or camphorated spirit, or by a short application of a mustard poultice. The tartar emetic should then be immediately rubbed in, either in the form of a warm saturated solution, or an ointment composed of one part tartar emetic to two of spermaceti ointment. With these precautions we have rarely failed to excite a full pustular inflammation in as short a time as that required for the rising of a blister, with far less irritation to the system, and with decided relief to the pectoral symptoms. We have reason to believe that a minute quantity of the tartar emetic is absorbed into the system, as we have sometimes observed nausea succeed its use; and this, instead of being injurious, as in the case of cantharides, is a part of the treatment which has been found most serviceable. This brings us to the subject of internal remedies.

At the onset of the disease, the earlier the better, a brisk purgative should be given. Where the inflammation is high, calomel, with a grain or two of ipecacuanha, combined with jalap, scammony, or any of the active cathartics, is to be preferred, and followed by repeated doses of a saline aperient, containing a small quantity of tartarized antimony. This medicine proves useful not only as an evacuant, but as a sedative and diaphoretic, and the nausea, which it sometimes produces, is generally beneficial by modifying the secretion of the bronchial lining, and facilitating expectoration. For the same reason, a full emetic has been generally recommended, and is often decidedly useful; but it is better adapted to children, and to cases in which the bronchial secretion is profuse, than to the more inflammatory forms of the disease.

For the relief of the dyspnœa and cough, those medicines are to be preferred which act on the vascular system. The sensation of dyspnœa is not to be looked on as one merely of discomfort, to be relieved by means which deaden sensibility, but as an indication of the inroad made on a vital function, which, if not restrained, must terminate in its destruction. To aid, therefore, the antiphlogistic measures that we have already recommended, it is proper to administer such medicines as are likely to moderate the action of the vessels and relieve the congested lungs. Tartar emetic, digitalis, and colchicum best answer this indication, and we generally prefer the two former. The tincture of digitalis, in the dose of eight or ten minims, with thirty or forty of the liquor antimon. tartar., three or four times a day, is the form which we usually adopt. Of course it will be necessary to watch the effects of these medicines, and to diminish or discontinue the digitalis if the pulse become intermittent. It will be of considerable advantage to increase the dose of tartar emetic; and if the dyspnœa continue, and the mucous rhonchus in the chest is undiminished, to persist in its use even should it excite vomiting; for its beneficial effects in peripneumony, to which bronchitis, in its severe form, really approaches very nearly, are sufficient to prove its power to restrain effusion and promote absorption. Dr. Badham strongly recommends this medicine in doses 'frequently repeated, and increased till the maximum which the stomach can bear without vomiting is attained.'

Calomel and opium combined, and given in frequently repeated doses, as in other acute inflammations, are likewise sometimes highly beneficial; and we consider that their use is especially indicated, where, as is not unfrequently the case, the bronchitis is complicated with gastro-hepatic disease. The opium should be in smaller proportion than usual, and is, perhaps, most safely given in the form of Dover's powder.

Should the collapsed stage have come on, and the symptoms of debility shew that the function of respiration has been already so much injured as to reduce the excitability of the system, it is necessary to resort to expectorants of the stimulating kind. Of these the best is carbonate of ammonia, which often enables a patient to expectorate what in his weakness might be sufficient to suffocate him. We are disposed to think that this remedy is more than an ordinary stimulant, and acts in an especial manner on the bronchial membrane.

The attenuating or solvent power of alkalies may with advantage be resorted to in the latter stage of bronchitis, when the matter of expectoration is very consistent, and, lodging in the bronchi, occasions local absence of the sound of respiration, and, if copious, dyspnœa. The liquor potassæ, in doses of ten minims, given with the vinum ipecacuanhæ, is productive of obvious benefit.

From what we know of the effects of the lobelia inflata in suffocative catarrh of the chronic kind, we should judge it to be worthy of a trial in the collapsed stage of acute bronchitis.

Besides attempts to relieve the lungs of the load which prevents the penetration of air into them, another indication presents itself in this stage, which is to obviate the bad effects of black blood in the system; for this it is, unquestionably, that gives that peculiarly depressed and adynamic type which precedes the fatal termination. Here, unfortunately, our experience fails us; and we feel the necessity of appealing to further researches of experimental physiology."

The subject of Bronchocele, from the pen of Dr. A. Crawford, concludes the third part of the Cyclopædia of Medicine. We shall notice the other parts in our next Number, while we strongly recommend this work as by far the best —indeed the only good work of the kind in the English language. It promises, in fact, to be a national concern.

---

### LXXXI.

### Cases of Entero-Vaginal Hernia,*

Entero-vaginal hernia has in a few cases been gradual in its formation, but in the majority it has suddenly followed some exertion, strain, or shock. The first case on record was related by

---

* Dr. Davis's Principles of Obstetric Medicine. Part VIII.

M. Garengeot, in the Memoirs of the French Academy of Surgery. As this is a rare yet important affection we shall give an abstract of some of the more interesting cases reported by Dr. Davis.

*Case* 1. The patient was the wife of a costermonger, of middle stature, who had borne five children, all large. About a month after her last confinement she made an effort to assist in lifting a load on the shoulders of a porter. At the instant of that effort she felt a derangement within the abdomen and an acute pain in the vagina, of which also she had a sense of great fulness. She consulted her midwife, who told her that she had a bearing down of her womb, and that she must place herself under the care of a surgeon. She however neglected this advice, and continued to busy herself with her ordinary affairs for some time subsequently. The descent into the vagina became so considerable that it manifested itself at the external orifice, beyond the level of which a tumour protruded about a finger's breadth. The patient felt from time to time pains of an internal spasmodic character, which seemed to take their origin from that part, as also pain of the stomach with sickness. Moreover, she was not able to void her urine except in the position of lying on her back. Informed of these particulars, M. Garengeot discovered on examination a whitish tumour, which not only occupied the vagina, but which projected beyond the labia pudendi in such a manner as to admit of the introduction of a finger between it and the vaginal parietes posteriorly. On passing his finger beyond the tumour in that direction, he could feel the orifice of the uterus occupying very nearly its natural situation: whence he concluded that that organ was not implicated in the existing mischief. He also found that the pressure which this examination caused to be made on the tumour, which indeed was unavoidable, had the effect of reducing it to about one half of its previous size. Such a change in the size of the tumour excited at once the suspicion that it consisted in a descent of intestine. With that impression on his mind he placed the patient in a lying position on her bed, and applied the taxis to the tumour with the utmost accuracy of attention. Whilst this operation was performing, he felt its contents as if receding through the superior and right lateral portion of the vagina: and after the retrocession was effected, it was left soft, relaxed, thin, and imperfectly occupying the space which had been previously occupied by the visceral protrusion. In order to strengthen his conviction that the case was one of entero-vaginal hernia, of which however he had never heard before, nor known any author who had described it, he desired the patient to get up and walk, and also to cough strongly. These movements caused the tumour to present itself immediately again. That circumstance served to establish M. Garengeot's conviction that it was actually a hernia. He effected its reduction a second time, and desired the patient to keep her bed until he should be able to supply himself with a suitable pessary. After some failure on this point, in the first instance, a well-adapted contrivance was at length applied, which had the effect of preventing any future descent so completely, that the patient never afterwards sustained any inconvenience from it.

*Case* 2. In the former case the recent confinement might seem likely to predispose the patient to the occurrence of such a hernia, but in the following there was no such apparent cause in previous operation.

A young woman, of good health and tallish stature, married at the age of twenty-four. Soon afterwards, having to walk along the unboarded joists of an unfinished house, she fell between them, and was precipitated with her face downwards on a heap of boards on the floor below. The face and temple were contused and she fainted for some time. On recovering her recollection she felt intense pain of the parts within the pelvis, referred more particularly to the bladder, and aggravated by the erect position. She thought that her bladder was ruptured, and could pass no urine when she attempted to do so. About two inches on the right side of the navel there was a lacerated wound, and contusion in the right flank. She was seen by Dr. Davis about three hours after the accident. She was in a state of great excitement, complained of great pain in the region of the bladder, with incapacity of passing her urine, any attempt at which was attended with great increase of the pain, tremendous bearing down, violent retchings, and painful gaspings for breath, which had more than once terminated in syncope. There had been a considerable discharge of pure blood from the vagina. On making an examination per vaginam, the region of the bladder was extremely tender to the touch, particularly in the space immediately anterior to the vaginal part of the uterus. No laceration was discovered, nor any sort of tumour or protrusion, but on making a second examination, during which the patient was seized with vomiting, an intumescence of such magnitude as very nearly to fill the upper and middle part of the vagina, soft and elastic, was found to proceed from the very termination of the vagina superiorly, intermediately between that part and the anterior lip of the uterus. The vomiting continued during the examination, and the swelling was exceedingly tense and painful.

Thinking that this tumour was the lacerated or displaced bladder, Dr. D. introduced the catheter without meeting any impediment, and drew off twenty ounces of bloody urine. This produced no relief, the vomiting indeed became more severe. The true nature of the case was suspected, the patient was again placed on her left side, with her breech raised and brought to the side of the bed, and by slow degrees the whole of the left hand was introduced into the vagina. Cautious and gradual pressure was made upon the tumour which was reduced in about a quarter of an hour, a gurgling noise accompanying the return. The mucous membrane of the vagina had not been ruptured, but formed an investing coat to the hernia, which had protruded at the part already mentioned. Towards this Dr. D. "bundled up" every fibre of tissue which might possibly be the remains of a hernial protrusion, and having procured a piece of fine sponge he employed it as a pessary, and found it answer extremely well.

The patient was now in a state of exhaustion, with very frequent small incompressible pulse, some disposition to vomit, great tenderness of the hypogastrium, and still some hæmorrhage from the vagina. In consultation with Dr. Sims, our author abstracted twenty-eight ounces of blood which produced deliquium, followed by marked relief. Leeches to the hypogastrium, a pill of calomel and opium every two hours, and strict quiet followed. We need not pursue the details of the case, suffice it that no more active means were required though judicious treatment was still necessary. A passage through the bowels was procured on the next day, and no further abdominal symptoms ensued. It was necessary to use the catheter for a fortnight, and the urine remained bloody for several days. The sponge pessary was withdrawn daily, and a fresh one imbued with a strong solution of alum substituted for it for about three weeks. Then, when the injured part of the vagina was pretty free from pain, a very light, hollow, and perforated wooden pessary, of nearly cordiform shape was substituted for it. This was withdrawn and re-applied daily for the first week, and after that left permanently in the vagina, without the riband, that the patient might not remove it herself. The pessary was discontinued after having been worn for four or five months, but sponge pessaries gradually reduced in size were continued for a few weeks longer. The patient was perfectly cured with the exception of a slight difficulty in micturition which has since remained, and she is now the living mother of several children.

Entero-vaginal hernia has been more frequently observed in the posterior than in the anterior chamber of the pelvis.

*Case* 3. An unmarried female, æt. 30, had been subject for many months to an excessive degree of costiveness, requiring great efforts in the expulsion of the fæces. During one of these efforts she suddenly experienced acute pain in the pelvis, extending to the left iliac region. It was violent for a moment, but afterwards continued with intermissions. Three weeks after this the patient discovered a swelling within the sinus pudendi, which increased and protruded from between the labia. She now consulted M. Hoin, who at first felt doubts as to the nature of the case. He found the tumour more distended and prominent when the patient was seated than when lying down, that it protruded half an inch beyond the level of the labia, extended about three inches within the vagina, and occupied its left side and rather posterior part. It was not large, nor was it painful except on firm pressure. By firm pressure it was reduced with a gurgling noise, leaving the vaginal parietes loose and bagging, but not so thin as in the preceding cases. Thus the hernia would seem to have occurred in this case between the uterus and rectum, in the former between the uterus and bladder.

*Case* 4. M. Leoret, in 1747, examined a case of intestinal hernia which occupied the left side of the vagina. The subject was an unmarried woman who had died idiotic. The tumour occupied nearly the whole of the vaginal passage; its reduction was easily effected. On opening the body the hernia was found to be one of the sigmoid flexure of the colon, and the acetabular portion of the ileum was much lower on the hernial side than on the other. The fundus of the uterus was directed obliquely towards the left side of the pelvis, its orifice towards the right.

"But it is much more important in practice to establish the proper distinctions between hernial protrusions of intestines into the vagina and other kinds of tumours occupying the same passage, than between the several varieties, at least in their simple forms, of such protrusions themselves. It might be very possible for a practitioner not much experienced in matters of this kind, to mistake, for instance, a case of hernial descent for prolapsion of the vagina. The practical result would of course be, that he would content himself with merely introducing a pessary into the vagina, without previously effecting the reduction of the hernia; and of such a blunder the consequences it is obvious might be very serious. A woman had for some years been the subject of an entero-vaginal hernia, which occupied the vagina anteriorly. She had sought no remedy, because she had considered her case to be one of simple bearing down of the vagina. She was, however, at length induced to consult M. Hacnel on the subject. That gentleman at once recognised the nature of her malady, and by an adroit adaptation of a sponge pessary to it, AFTER PREVIOUSLY REDUCING THE HERNIA, effected a speedy cure of it. Gunz de Herniis libellus, p. 84, et seq. The same author refers, however, to another history, where a mistaken diagnosis in a case of the same kind, suggested an operation which proved fatal to the patient. "A woman was the subject of a tumour which occupied one side of her vagina, and which presented itself at the orifice of that passage. She consulted a surgeon, who, it was believed, mistook it for an abscess, and plunged his bistouri into it. The incision was immediately followed by a protrusion of the cæcum, and of a great part of the colon, which were strongly propelled through the incised aperture by the involuntary efforts of the patient, increased no doubt at the moment by the pain from the infliction of the wound. The protruded bowels were not attempted to be reduced. Gangrene of course supervened, and the poor woman died.' Prof. Gunz. loco citato."

Several other cases of entero-vaginal hernia are related from the work of Sir Astley Cooper; we need not notice them. But this sort of hernia may occur in complication with pregnancy, and the practitioner may inquire how such a case is to be managed. The following instance which occurred to

Dr. Smellie, and is detailed by him, will shew the most successful mode of procedure.

*Case* 5. In the year 1731, Dr. S. was called to a woman who had felt a swelling on the left side of the anus, which had gradually increased. It disappeared in bed but returned when she rose. It continued down all the time of her first labour, inflammation and strangulation of the intestine having occurred, and prevented reduction. She had a large discharge of blood after delivery; and, with the assistance of fomentations, the hernia was now returned. In the next labour, the intestine was again forced down. The progress of the labour was early and rapid; but before the head of the child had descended into the pelvis, Dr. Smellie was enabled, by passing his hand into the vagina, and pushing the head above the os sacrum, to reduce the hernia. The membranes were ruptured by the operation, the waters discharged, the head forced rapidly down, so as to occupy the space before tenanted by the tumour, and the patient was safely delivered.

In a case which occurred to Dr. Ross, at St. George's Hospital, and was by him transferred to Dr. Smellie, the hernia inflamed, and ulceration, apparently into the intestine, took place. Immediately on this the gut went up, and for some time remained so, and the patient recovered. Subsequently it descended again, in consequence of over-exertion, and the patient again became pregnant. The tumour was reduced during gestation, and the patient was safely delivered at the full time. She died soon afterwards of small-pox, and no examination appears to have been made, a circumstance certainly to be regretted.

From the foregoing cases, our readers will readily perceive the general symptoms, characters, and course of utero-vaginal hernia, the dangers to be dreaded, and the most appropriate treatment, both at the time of its occurrence, and subsequently to prevent its re-appearance. There are some other complications of this affection, which we may notice on another occasion. We think that this work of Dr. Davis's is likely to prove very useful, not only to gentlemen engaged in obstetric practice, but also to those occupied with other divisions of professional pursuits. Yet we cannot refrain from objecting to the diffuseness of the style, the almost interminable length of many of the cases, and the tiresome introduction of circumstances, quite irrelevant to the subject, which defaces most of them. The work, in its present fashion, might be extended, for any thing that we can conceive to the contrary, to a goodly number of volumes. Dr. Davis's resources may be great; but he must not, therefore, imagine that a book is rendered more pleasing, or more instructive, from being unnecessarily crowded with a multitude of facts differing little from each other, and those facts enveloped in such a shell of verbiage, that it is equally difficult and laborious to crack it. The object of Dr. Davis should be to select, abridge, condense his matériel, not to expand it, nor suffer it to expand. We make these observations with good will and good intentions towards the able and most industrious author, and we trust they will be taken in equally good part. We are sure that it will be both for the advantage of his work and for his own, to attend to the suggestions we have made.

---

### LXXXII.

### Hackman on Softening of the Spleen.*

We know but little at present of diseases of the spleen; and what we do know relates rather to the changes observed in the bodies of the dead, than the symptoms of those changes in the living. Here is a pregnant negative instance of the value of physiology. Symptoms are merely alterations of functions or of structure; we recognize diseases of parts or of systems con-

---

* Hecker's Litterarische Annalen der Gesammten Heilkunde; Med. and Phys. Journal, 1832.

cealed from sight, by functional disturbances—we determine diseases of more external organs by aberrations from their healthy physical structure. But we know not the function of the spleen in health, and how should we recognize the alterations of that function in disease? In fact we cannot do so; and probably our impotence on this point will be perpetual. If the spleen be so altered in structure, enlarged for instance, as to afford physical signs of its disease, we can and frequently do detect it; but we neither do, nor do we believe that with our present amount of knowledge we possibly can, go much farther than this. We remember a remarkable case in point. A young man laboured under symptoms of a very dubious character. He had symptomatic fever, and yet it was difficult to determine of what the fever was symptomatic. Nothing amiss was discovered in the chest, nor in the head, nor in the abdomen; and yet the functions of the viscera of all these cavities were obviously disturbed. We believe the patient complained of pain in the region of the stomach and had vomiting. Many gentlemen saw this patient, but no two agreed in their opinions; and whilst the Doctors were differing, he died. On examining the body, the spleen was found full of tubercles, advancing or advanced to suppuration, and no other lesion was discovered.

But a good practitioner is not he who merely knows what is generally known, or remembers what has been written since the ancient Greeks, unlike the modern European descendants of the Goth and the Hun, made a deity of their physician. A good practitioner is he who reasons and compares, who remembers what he has seen, and calculates probabilities. By this process of reasoning and comparison new avenues of information are opened to us, and when we cannot get certain knowledge, we obtain inferential. If, for instance, a patient is presented to us with symptoms of a grave character, yet without those signs which are known, by observation, to indicate alterations of the more important organs, the judicious practitioner will be led to suspect affections of parts not so prominent in their actions, not so obviously essential to life, yet carrying destruction with their diseases. As a matter of experience, we know that long-continued intermittents are often accompanied with alterations of the spleen, and from this knowledge we derive much assistance. We also know that, in certain epidemic pernicious fevers, the spleen undergoes more perceptible alterations than other organs. From facts of this kind, no certain good appears at first sight to spring; and yet, when considered, they afford much useful knowledge. We see that in fevers, in stages of which the blood leaves the surface and collects at the centre, the spleen is liable to suffer; in fact, that abdominal congestions form, perhaps, the most prominent causes of splenic disease. The spleen is essentially a viscus of blood-vessels and blood, and accordingly we find its chief alterations in those states, in which the blood itself appears to undergo some alteration, as it does in the congestive fevers, whilst it suffers mechanically from the mere congestion, as in simple intermittents. In the former class of cases, we generally observe softening of the spleen, in the latter, unnatural growth. The diseases of the spleen, independent of these, are, on the whole and in comparison with those of other organs, few. This brings us, then, to the consideration of Dr. Hackman's observations on softening of the spleen.

"Affections of the spleen form a considerable proportion of the disorders peculiar to women; but in low and marshy countries, whether towards the tropics or the poles, they are much more common than with us, and are known as the sequelæ of ordinary intermittent and remittent, and also of yellow fever. The regions most remarkable in this respect are the borders of Denmark, East Friesland, the island of Walcheren, La Vendée, several districts of Hungary (especially Bannat), and many parts of upper and middle Italy, and, among others, the Pontine marshes and the Campagna near Rome: to these may be added Minorca, South Carolina, Bengal, &c. Of the diseases of the spleen common in the above-named countries, Ramollissement must not be overlooked. The generality of works, however, on the pathology of this organ scarcely mention such a state, and even HESSE himself dismisses it very briefly in his excellent treatise on Ramollissement.

Softening of the spleen is sometimes sporadic, but more frequently it is either epidemic or endemic : in the latter cases it is intimately connected with other diseases which are in themselves epidemic or endemic likewise. *Splenomalacie* is frequent, not only in the human subject, but is common also among animals, especially among the ruminantia. When sporadic, this affection follows sporadic intermittents ; when epidemic, it accompanies epidemic fevers in hot and marshy countries. In the last epidemic which ravaged the north of Germany, softening and hypertrophy of the spleen were phenomena so constant, that Dr. DOHRN even ventured to call the disease *splenitis epidemica contagiosa.* Fevers in Sardinia likewise often terminate in softening of the spleen ; this lesion, however, is always the effect of a more general disease ; at least the author has never met with an idiopathic case.

According to the opinion of Hachmann, softening of the spleen depends upon over-congestion, or venous inflammation, of which there are two stages, one of irritation or congestion, and one of true ramollissement. The symptoms of the first are, fever with gastric disorder predominating, but of which the type will vary, according to the season, the climate, or the constitution of the patient. In hot and marshy countries, or even in more temperate parallels during the summer, the access of fever is preceded by an ordinary shivering, but the intervals are so slight that it might perhaps be considered a remittent. The rigors, which vary in duration and intensity, are followed by a burning heat, after which a profuse perspiration prevails. At the beginning of the attack, the patient vomits a clear fluid, often mixed with bile ; which ejections continuing during the cold, cease on the access of the hot stage, and recur with the recurrence of each fit. In northern climates, these symptoms diminish as the disease advances ; while, in tropical countries, they continue unabated, as the black vomit of the yellow fever will witness. Hæmatemesis only occurs in chronic cases of spleno-malacie. Another essential symptom of this disease is precordial distress marked by similar exacerbations and remissions with the fever, and which probably depend upon the compression of the diaphragm by the enlarged spleen. This symptom is never entirely absent in any case, and sometimes it is deve-veloped in so great intensity as to become truly *orthopnœa.*

Of other diagnostic signs of this disease the principal are, great lassitude, pains in the limbs, vertigo, flushed face and eyes, and great thirst, (which, if satisfied, increases the præcordial pain ;) the tongue at first is of a pale red, and covered by a yellowish fur, by degrees it becomes of a bright red, and cracks ; but it remains generally moist, and is often studded with aphthous eruptions. The abdomen is swelled, soft in the umbilical region, but not tender, pain being only felt when the body is bent and pressure made towards the stomach or spleen. In the epigastrium, a very sensible pulsation may be perceived ; and as the disease advances, this pulsation extends also to the region of the spleen. Most frequently the fever is ushered in by diarrhœa, (ten or twelve evacutions in twenty-four hours ;) the matter passed being dark coloured, greenish, watery, and very fetid. It is during the febrile exacerbations that the stools are most frequent ; and this circumstance, joined to the vomitings, gives to the disease an appearance of cholera. The patients are excited, they sleep but little, wander much, and become delirious ; the pulse is variable, at first full and soft, afterwards becoming small and very frequent ; sometimes, however, it does not vary much, but remains slow, and occasionally intermitting.

The stage of ramollissement seems to be shewn by a great collapse, and the access of typhoid symptoms, and death ensues as in cases of fever, or follows a state of coma resembling apoplexy. This apoplectic state is a phenomenon that frequently occurs in ramollissement of the spleen.

The progress of *spleno-malacie* is, in general, rapid, like that of the fevers of which it is the result: thus, ramollissement may take place in the course of eleven or twelve days. Still the disease may assume a chronic form, as two cases recorded by BONET and PORTAL, and one observed by the author, have sufficiently shewn.

There are different degrees of ramollissement: in the first, the spleen is gorged with blood of a black or dirty brown colour, amongst which the reticular tissues cannot be distinguished; the structure is more friable than when in a healthy state, and pressure will discharge a great quantity of dark-coloured blood, so that there will remain nothing but a sac containing black blood, and a soft matter resembling chocolate.

In the more severe forms of ramollissement, the spleen bursts spontaneously, without any previous violence, during the life of the patient, and the matter escapes into the abdominal cavity, when death speedily ensues. The volume of the viscus thus softened is not always much increased, but most frequently the bulk is considerably augmented if the individual has suffered from endemic intermittent or remittent fever.

Of the mode in which ramollissement of the spleen is effected nothing more is known than of ramollissement in general; however, most pathologists agree that such morbid alterations result from sanguineous congestion and inflammation, disorganising the viscus, and ending in gangrene.

Towards the conclusion of his memoir, the author relates eight cases of acute spleno-malacie, extracted from the writings of HEUSINGER, GROTANELLI, MONTFALCON, and BAILLY, and one chronic case which fell under his own observation."

---

### LXXXIII.

### AUSTRIAN STRICTNESS IN DISCHARGING SOLDIERS.

IN Mr. Marshall's excellent work on the enlisting, discharging, and pensioning of soldiers, lately published, we are informed of the method adopted by the Austrian government in the discharging of their men. In countries like Austria, where the government is despotic and supported in a great degree by the bayonet, it becomes a matter of imperative necessity to reduce their armies to mere machines, powerful it is true, but flexible to the will or caprice of the despot of the day. For this purpose discipline must be carried to its utmost extent, and the soldier must be drilled into his military duties till he scarcely comprehends the existence of any others. We dare say that this discipline is sufficiently irksome, at least to many, and that attempts are consequently made by malingerers to retreat from the service. However this may be, it is certain that the utmost precautions are adopted to prevent the success of any such schemes, and the man who has sufficient wit to slip through the scrutiny which is adopted, must be a malingerer of the first water.

"In the Austrian Military Service, there is a medical practitioner attached to each troop or company of a regiment. This class of officers is not authorized to propose disabled soldiers to be discharged without the sanction of a "*field-surgeon*," who decides upon the cases of the men alleged to be disabled: in the performance of this duty he is, from his office, denominated the *Arbitrium*. For the purpose of dividing the responsibility in this important duty, the Arbitrium is associated with a military officer of a rank equal or superior to that of the persons about to be examined. When non-commissioned officers or privates are examined, the field surgeon is associated with an officer of the troop or company to which the men respectively belong; when a subaltern or a captain is to be examined, he is associated with an officer of equal rank with the individual alleged to be disabled. If a staff-officer is to be examined, the Arbitrium is associated with a general-officer. Officers, of whatever rank, who may be employed on this duty, are individually responsible for the accuracy of their reports of the cases of the men who are submitted to their examination. These cases are to give a detailed account of the origin and progress of a man's disease or disability, and the means which have been employed for his recovery. When an alleged disability is not obvious, such as in the case of disease of an internal organ, or when it is periodical, a great body of competent evidence is required to establish the allegation of a man's invalidity. After the men have passed the *Arbitrium,* or first Board, they are brought before a *Super-arbitrium,* or second Board, which is assembled by order of a general officer. The super-arbitrium is allowed three weeks to perform this duty, for the purpose of affording time to collect evidence, or otherwise to acquire satisfactory information regarding doubtful cases. The men who are examined by this Board, are divided into three classes, namely, *completely disabled, half disabled,* and *temporarily disabled;* in other words, unfit for service, fit for garrison or reserve duties, and cases who may recover by medical treatment. Malingerers are severely punished. Sometimes they receive corporal punishment, and at other times they are sentenced to serve for life. Invalids who have been discharged are not exempted from the surveillance of a general-officer, who may summon them before him, and if it be found that they have regained their health and efficiency, he may erase their names from the list of pensioners. The Arbitrium, and the Super-arbitrium, are both held responsible for whatever errors they may commit, and they may be called upon to refund the amount of any expenses that have thereby been incurred."

### Mortality of Young Recruits in Hot Countries.

It requires no great penetration to foresee that the young are not those who are likely to bear hardships best. Manhood is the period of vigour corporeal and constitutional, and this has been proved to a remarkable degree in our Indian armies. Dr. Burke, Inspector General of Hospitals dwells very strongly on this point in his Medical Report of the King's Army in Bengal.

"The period of life for the young soldier coming to India should be, for the youngest, the age of formed manhood, and when his habits have been fixed. He should, moreover, have been drilled before coming to this climate, in which so much is unavoidably suffered by the fatigue which the young soldier must undergo in learning the first parts of his exercise. In short, the soldier should arrive here at the age and period when he can be of the greatest use when called upon for active service. That youth should ever be the proper time, the records of every corps here disprove the assumption. The coming to India at the mature age of twenty-five or twenty-six, or full grown manhood, having been found the most favourable to health and less so to disease in India—that I conceive to be the period of life which should be fixed upon as the most proper for sending out young soldiers to this country."

A regiment arrived in India in 1823. It was then 653 strong, and had received in the course of six years 1525 recruits and volunteers from other regiments leaving India. During the years 1824 and 1825, the regiment was employed in the Burmese territory. By a table it appears that, in 1824, the ratio of mortality among the young men who went out with the corps was 38 per cent, or 1 in every $2\frac{1}{2}$; while among the volunteers who were considerably older, the mortality was 17 per cent, or 1 in 6. In 1825, it was 30. 5 per cent. or 1 in $3\frac{1}{3}$ among the former or younger class, and only 6 per cent. or 1 in 16 among the latter or older. In 1826, the total strength of his Majesty's Army in Bengal was 7712, of whom 15 per cent. were under 20 years of age. Since that period the age of recruits has been a subject of consideration with the government, and no regiments serving in tropical climates enlist recruits under the age of twenty, a very humane and wise regulation.

"The French admit healthy and robust volunteers under thirty years of age into the service, and soldiers are allowed to re-engage until they are thirty-five; should they, however, have passed the age of thirty, they will not be received into any other branch of the service than the one in which they had formerly served. The greatest duration of voluntary engagements in the French service is eight years. In services such as ours, where recruits engage for an unlimited period, it is of peculiar importance that men should not be enlisted at an advanced age. Experience tells us that an extremely small proportion of the men of the British army serve beyond the period of forty years of age, probably not above 4 per cent.; hence it would be a measure of economy, as well as a means of contributing to the efficiency of the army, not to enlist men above twenty-five or twenty-six years of age. But there is another consideration which ought not to pass unnoticed. A great number of the men who wish to enlist after twenty-six years of age are dissipated and profligate characters, or they are persons who have been unsuccessful in business, broken-down gentlemen, &c. &c., and, on account of these causes, are frequently disqualified for the army. It is of importance that the point of excellence, in regard to the age of recruits, should be appreciated and recognized; which being once carefully fixed, the limitation may be enlarged or contracted according to the wants of the service. A measure may be expedient, although it be not the best which might be devised."

### Malingering.

Many very curious examples of the ingenious knavery, and knavish constancy of malingerers have been at different times recorded. The game is probably more difficult now than it was formerly, and few are able to elude the well directed vigilance of our present medical officers of the army and navy. The following are amusing instances of the systematic attempts at deception practised by men anxious to obtain their discharge from the service.

Many have entirely destroyed one eye in order to obtain the pension of ninepence per day which government allotted to them. It was proved before a committee of the House of Commons, that in a barrack where several hundred cases of inflammation of the eyes occurred, a large proportion had been occasioned by the introduction of gonorrhœal matter into the eye. As soon as a regiment is ordered to the West Indies, disease of the eyes very frequently appears among the men, and continues till the corps has embarked. The number of cases in the depôt then decreases, and no more is heard of it till the period for embarkation comes round again.

"Among numerous instances of strong presumptive evidence of the existence of factitious disease of the eyes which have come to my knowledge, I shall adduce the following:—Early in the year 1827, several severe cases of inflammation of the eyes occurred in —— regiment, which was then employed in this country, and about the middle of July the disease became very prevalent. From the commencement of the affection, strong suspicions were entertained that it was artificially excited; none of the officers or children were affected with it, and but one woman. Only three non-commissioned officers were admitted into hospital, although men who had been reduced on account of misconduct, seemed to be particularly liable to the affection. Change of quarters had no influence in arresting its progress; various means were adopted with a view to discover whether or not the disease was factitious, and kept up by artificial means. The men were repeatedly removed, without warning, from their beds in one ward to another, and the first ward minutely examined for the purpose of discovering whether they had any irritating substances calculated to produce inflammation of the eyes concealed among the bed-clothes or, elsewhere. Small parcels of snuff, tobacco, and salt, were found in or close to the beds of several of the men, and it was presumed that the patients had been in the habit of introducing these substances between the eyelids. Low diet, confinement to bed, and similar measures, seemed to have no influence in facilitating recovery, or in diminishing the number of new admissions. With the view of preventing the patients from having access to their eyes, some head-pieces of tin plate were constructed, which, when put on, could be secured by means of a lock and key. These machines were procured during the Autumn of 1829, and, by their judicious use, the progress of the epidemic was arrested.

From the commencement of the epidemic, till the 24th of October, 218 men, (not admissions only,) were received into hospital on account of inflammation of the eyes. Thirteen lost each an eye, eight that of the right, and five that of the left. Four have been discharged from the service, and nine are doing duty. At present, (24th of October,) there are only four cases of eye diseases in hospital."

With the following rather ridiculous instances of the detection of impostors we conclude.

During an alleged paroxysm of epilepsy, where imposition was suspected, Mr. R. exhibited seven or eight drops of croton oil through an opening left by the loss of two teeth, and in a few minutes the man started on his feet and ran to the water-closet.

A soldier belonging to —— regiment said he had lost the power of his right arm, but, from the absence of any physical evidence of disease, the medical officer considered him a malingerer, and tried various means to induce him to return to his duty, but without success. With the view of intimidating him, a proposal was made to amputate the arm, and, in prosecution of this end, an unusual degree of solemnity was observed on the occasion. All the surgical instruments which could be mustered were displayed: but nothing daunted, he allowed himself to be conducted to the chair preparatory to the operation. The tourniquet was put on, and the amputating-knife placed under his arm ready to make the first incision. He sat unmoved. The surgeon was puzzled, and made the best excuse he could for postponing the operation. Being, however, still impressed with the opinion that the arm was not disabled, he resolved to attempt another means of conviction. He recommended change of air for some of his patients, and among others the alleged case of palsy. To reach the place announced for the accommodation of the sick, it was necessary to cross the —— river in a boat. The party embarked, accompanied by the surgeon, who, by a preconcerted signal, directed the boatman to throw the man in question, who was a good swimmer, into the river. After a little time he seemed to be much exhausted from the exertions necessary to keep him afloat by means of his left arm only. The surgeon became alarmed, and had resolved to take his patient on board, when he uttered an oath, and struck out vigorously with both arms. The conviction was conclusive.

---

### LXXXIV.

### Iodine for Secondary Eruptions of Syphilitic Origin.*

Mr. Mayo appears to be making some experiments with iodine. The form in which he exhibits it is the following. ℞. Tr. iodinæ, ʒj. Potassæ hydriodatis, ʒss. Syrupi papaveris, ℥ss. Aquæ menthæ viridis, ℥viij. ad ℥x. This quantity to be taken by adults during the twenty-four hours; a less quantity by children. If Mr. Mayo begins with this dose, we should imagine that at some time or other he will find it produce deleterious effects. We have seen very severe constitutional disturbance from less quantities, begun too with more caution than Mr. Mayo seems to employ. Mr. M. has only found iodine beneficial in cases of bronchocele and in some affections of the skin of syphilitic origin. This is no novelty. Mr. Brodie has employed it in the treatment of secondary symptoms for some time, and we saw two patients so treated in St.

---

* Med. and Phys. Journ. June, 1832.

George's Hospital last year. If our memory serves us they left the hospital too early to enable a conclusion to be drawn as to the result. We have seen iodine employed in one or two other cases of secondary symptoms, but we confess without much advantage. In the following cases more benefit appears to have been derived from it.

*Case* 1. John O'Shaughnessy, æt. 32, admitted in the Middlesex Hospital in January, 1831. There was a large superficial ulcer on the instep, in great part covered with healthy granulations, but with a yellow phagedenic edge and a narrow bright red line upon the skin around it; there was a similar ulcer on one shoulder, and several large leprous spots on the limbs and body. He stated that in the preceding August he had contracted a chancre with bubo, for which ptyalism had been procured by mercury, with the effect of healing both chancre and bubo. As far as his recollection served him the throat became ulcerated about five weeks after he commenced the mercury and while he was still taking it; on one occasion he lost by a sudden hæmorrhage nearly a quart of blood. Copper-coloured spots followed by ulcers afterwards appeared upon his body, and he believed that mercury was then discontinued, and that he took sarsaparilla, under which he mended.

Mr. Mayo put him on sarsaparilla with liquor potassæ. The ulcer on the instep healed, but he lost strength, and the skin became covered with blotches and ulcers. The blotches at first resembled lepra alphoides, but soon each spot became tubercular, and, in place of the thickened white patch of cuticle, a crust formed, below which was an ulcer. There were many of these on the limbs, body, face, alæ nasi and lips, where they occasioned a profuse ptyalism; there was a small depôt of serous fluid in the calf of the right leg; there was no affection of the bones. Many means were tried with temporary amendment. Blue pill and nitric acid did decided mischief. In December M. Magendie suggested to Mr. Mayo the use of iodine. Its effects, after a few days use, were striking; the skin was less red and heated, several of the crusts separated, and the ulcers looked healthier. The remedy has been persevered in to the present time with interruptions of a fortnight on four different occasions. The patient is "all but entirely" recovered.

*Case* 2. John Baxton, æt. 31, was admitted with ulcerated throat and face, and pains and tenderness of the head. These pains began in a venereal sore and course of mercury. It is now two years since the forehead became at two points puffy and tender, then red, and broke. Ulcers formed on the forehead, eye-brows, and bridge of the nose, round which the skin was red and thickened; they spread after healing where they began and extended over a fresh surface. Many plans were tried and all ultimately failed. The decoctum smilacis asperæ at one time, and a mild mercurial course at another, produced most benefit. The patient began the iodine at the same time with the subject of former case. He has entirely recovered, the ulcers having gradually healed, and the adjacent skin having lost in a great degree the redness and tumefaction.

These cases are calculated to encourage practitioners in making trials with iodine in that miserable class of secondary symptoms, where a definite character is lost, where, from mismanagement or some peculiarity in the original poison or in the system which received it, the case is neither strictly syphilitic, mercurial, nor cachectic, but a villainous compound of all. Every now and then we see a wretch in the General or Lock Hospitals, covered with blotches, frequently in combination with destruction of the nose or of the throat, perhaps with carious vertebræ, or with laryngeal affection, and his limbs distorted by chronic thickenings of the periosteum. Such a miserable creature is driven from pillar to post, from sarsaparilla to the oxymuriate, from the oxymuriate to the smilax aspera, from this to inunction, and too often the effect of all this treatment is to place him in a more pitiable condition than before, to make the case such a composite thing that no art can unravel it, and death from phthisis, serous inflammation, or some casual attack of erysipelas, destroys at last the unhappy victim. If iodine should prove of benefit in such cases, it will be well; but it will need many trials and much discrimination to determine its real powers, and to ascertain the circumstances favourable to the development of its good effects.

---

